# Molecular Characterization and Expression Analysis of *YABBY* Genes in *Chenopodium quinoa*

**DOI:** 10.3390/genes14112103

**Published:** 2023-11-19

**Authors:** Tingting Li, Mian Zhang, Mengyao Li, Xinxin Wang, Shuping Xing

**Affiliations:** 1 College of Life Science, Shanxi University, Taiyuan 030006, China; ltt100518@163.com (T.L.); 13753723169@163.com (M.L.); w2233914175@163.com (X.W.); 2 Institute of Applied Biology, Shanxi University, Taiyuan 030006, China; zhangmian7285@sxu.edu.cn; 3 Shanxi Key Laboratory of Nucleic Acid Biopesticides, Taiyuan 030600, China

**Keywords:** quinoa, *YABBY* genes, *cis*-elements, gene expression, abiotic stress

## Abstract

Plant-specific YABBY transcription factors play an important role in lateral organ development and abiotic stress responses. However, the functions of the *YABBY* genes in quinoa remain elusive. In this study, twelve *YABBY* (*CqYAB*) genes were identified in the quinoa genome, and they were distributed on nine chromosomes. They were classified into FIL/YAB3, YAB2, YAB5, INO, and CRC clades. All *CqYAB* genes consist of six or seven exons, and their proteins contain both N-terminal C2C2 zinc finger motifs and C-terminal YABBY domains. Ninety-three *cis*-regulatory elements were revealed in *CqYAB* gene promoters, and they were divided into six groups, such as *cis*-elements involved in light response, hormone response, development, and stress response. Six *CqYAB* genes were significantly upregulated by salt stress, while one was downregulated. Nine *CqYAB* genes were upregulated under drought stress, whereas six *CqYAB* genes were downregulated under cadmium treatment. Tissue expression profiles showed that nine *CqYAB* genes were expressed in seedlings, leaves, and flowers, seven in seeds, and two specifically in flowers, but no *CqYAB* expression was detected in roots. Furthermore, *CqYAB4* could rescue the *ino* mutant phenotype in *Arabidopsis* but not *CqYAB10*, a paralog of *CqYAB4*, indicative of functional conservation and divergence among these *YABBY* genes. Taken together, these results lay a foundation for further functional analysis of *CqYAB* genes in quinoa growth, development, and abiotic stress responses.

## 1. Introduction

Quinoa (*Chenopodium quinoa* Willd.), a pseudocereal crop belonging to the Amaranthaceae family, was originally cultivated in the Andean region of South America [1,2,3]. People of that region consume quinoa seeds as food and quinoa greens (leaves, sprouts, and microgreens/seedlings) as salad [4]. Quinoa seeds contain essential amino acids, minerals, vitamins, carbohydrates, and lipids required by the human body, and they are more nutritious than other staple grains [5,6,7]. Quinoa greens also contain high nutritional and functional compounds with beneficial anticancer, antioxidant, and anti-obesity properties [4]. Furthermore, quinoa seeds are gluten free, and they have a low glycemic index. They may thus constitute a large part of the diet for people suffering from coeliac disease, wheat allergy, diabetes, or diseases of the heart or blood vessels [8,9]. In addition, quinoa plants are tolerant to many stresses, including drought, low temperature, ultraviolet irradiance, and salinization, enabling them to grow in adverse conditions, such as arid soil, highlands, and soil with high salinity [8,10,11,12,13,14]. Given the characteristics of high nutritional value and stress tolerance, quinoa was rediscovered in the eighties and has since been cultivated in more than 100 countries [4]. In particular, quinoa is a climate-change-resilient plant with potential to meet the global food demand for the constantly growing population [15]. To emphasize the importance of this crop, the Food and Agriculture Organization of the United Nations declared 2013 as “The International Year of the Quinoa” [6]. The research on quinoa has also been increasing in many different aspects. A high-quality reference genome of quinoa was published in 2017 [16], thus providing a fundamental paradigm for understanding the molecular mechanism underlying these advantageous agronomic traits of quinoa and finding key genes in gene regulatory networks, which could be useful for performing molecular and genetic breeding in quinoa and other major crops [7]. 

Transcription factors (TFs) are key regulators of gene expression, either alone or combined with other factors, by binding to the *cis*-elements of their target genes. One gene can control an important agronomic trait; for example, *OsTCP19* regulates the rice tillering response to nitrogen [17]. More than 60 TF families are found in plants, and some of them are plant specific [18]. Eleven TF families have been investigated genome-wide in quinoa, including NAC [19], WRKY [20], SPL [21,22], bZIP [5], bHLH [23], trihelix [24], R2R3-MYB [25], BBX [26], ARF [27], VOZ [28], and GRAS [29]. They play vital roles in developmental processes and diverse stress responses. *YABBY* genes constitute a small TF family and appear to exist only in seed plants [30]. A feature of the members of this family is that they contain two DNA binding domains, which are the N-terminal C2C2 zinc finger motif and the C-terminal high mobility group (HMG)-box, termed the “YABBY” domain. YABBY TFs can regulate gene expression as both activators and suppressors, and the functions of *YABBY*s correlate with their expression profiles directly or indirectly [31,32,33].

The *Arabidopsis* genome has six *YABBY* family members, in which the founding member *CRABS CLAW* (*CRC*) is specifically expressed in the carpel and nectary of the flower [34]. Analysis of the *crc* mutant phenotypes indicates that this gene is involved in early radial gynoecium growth and in promoting its later elongation, as well as the initiation and development of the nectary [34]. Besides *CRC*, *INNER NO OUTER* (*INO*) is exclusively expressed in the outer integument of the ovule, and it is essential for its formation and asymmetric growth [35]. *CRC* and *INO* are designated as “reproductive *YABBY* genes.” In contrast, the other four *YABBY* genes, which are *FILAMENTOUS FLOWER* (*FIL*), *YABBY3* (*YAB3*), *YABBY2* (*YAB2*), and *YABBY5* (*YAB5*), are referred to as “vegetative *YABBY* genes.” They are expressed in the abaxial domains of all leaf-derived organs, including cotyledons, leaves, and floral organs. The triple and quadruple mutants of these genes exhibit a small and bushy plant phenotype in which all lateral organs have lost polarity identity, indicating that these *YABBY* genes redundantly control lateral organ identity. In addition, these genes are responsible for maintaining the proper activity of the meristem [31,36]. *YABBY*s were also found in rice, maize, and wheat [37,38,39,40] and found functioning in carpel specification and leaf midrib formation [41], the development of vasculature [42], the determination of the fate of abaxial cells in leaf development and architecture [43,44,45], lateral organ development and meristem maintenance in spikelet [46,47,48], seed shattering [49,50], and the regulation of gibberellin metabolism [51]. Recently, a few studies have shown that *YABBY* genes are involved in abiotic stress responses, such as salt and drought stresses [52,53], cadmium (Cd) stress [54], and cold and heat stresses [55].

To explore the functions of the *YABBY* family genes in quinoa, we isolated the *YABBY* genes in the quinoa genome, mapped them on chromosomes, characterized their phylogenetic relationship, gene structures, and protein motifs, and analyzed their expression profiles (in particular, their expression responses under salt, drought, and Cd stresses). Moreover, the capability of *CqYAB4* and *CqYAB10* in rescuing the *ino* mutant phenotype was assessed in *Arabidopsis*. The results indicate that the quinoa *YABBY* genes may play a role in leaf and flower development as well as in salt, drought, and Cd stress responses.

## 2. Materials and Methods

### 2.1. Plant Materials, Growth Conditions, and Stress Treatments

Quinoa plants were grown in a growth chamber at 23 ± 1 °C, 60% relative humidity, and a 16 h/8 h light/dark cycle. The plant samples were harvested from two-week-old seedlings, four-week-old plants, and seeds produced after flowering.

To examine the expression of *CqYABBY* (*CqYAB*) genes under stress conditions, quinoa seeds were first incubated in a Petri dish with moist filter paper. Three-day-old seedlings were then transplanted into the holes of a 96-hole plate, placed in a 2 L container filled with ½ Hoagland nutrient solution, and grown in the growth chamber. On the seventh day after transplanting, the seedlings were handled with different treatments: (1) control seedlings were kept in the nutrient solution throughout the experiment; (2) salt stress seedlings were planted in nutrient solution with 200 mM NaCl; (3) drought stress seedlings were planted in nutrient solution containing 15% polyethylene glycol (PEG6000); and (4) Cd stress seedlings were planted in nutrient solution with 100 μM CdCl_2_. The seedlings were sampled 2 h after each treatment and stored at −80 °C until analysis.

### 2.2. Identification of *YABBY* Genes

The YABBY protein sequences from *Arabidopsis thaliana* were downloaded from The Arabidopsis Information Resource (TAIR; http://www.arabidopsis.org, accessed on 11 July 2023). These YABBY sequences were used as input sequences to perform BLAST searches in Phytozome v13 (https://phytozome-next.jgi.doe.gov, accessed on 15 July 2023) and the National Center for Biotechnology Information (NCBI; https://www.ncbi.nlm.nih.gov, accessed on 15 July 2023) to obtain the protein, coding, and genomic sequences of the *YABBY* genes from *C. quinoa*, *Spinacia oleracea*, *Beta vulgaris*, and *Oryza sativa* (Table S1 and Supplementary File S1). 

The physicochemical properties of CqYABs, including molecular weight and theoretical isoelectric point (pI), were predicted using the ExPASy web tool (https://www.expasy.org, accessed on 20 July 2023). The subcellular localization of CqYABs was predicted using the Plant-mPLoc server (www.csbio.sjtu.edu.cn/bioinf/plant-multi/, accessed on 22 July 2023), CELLO (www.cello.life.nctu.edu.tw, accessed on 22 July 2023), and WoLF PSORT (http://wolfpsort.hgc.jp, accessed on 23 July 2023).

### 2.3. Chromosomal Location

The position of each *CqYAB* gene in the chromosome and the chromosome size were obtained from Chenopodium DB (www.cbrc.kaust.edu.sa, accessed on 25 July 2023). Gene locations were visualized with MG2C (http://mg2c.iask.in/mg2c_v2.1, accessed on 28 July 2023).

### 2.4. Sequence Alignment and Phylogenetic Tree Construction

Amino acid sequences from the YABBY family members of *A. thaliana*, *C. quinoa*, *S. oleracea*, *B. vulgaris*, and *O. sativa* were aligned using ClustalW. A phylogenetic tree was generated through the neighbor-joining method with a bootstrap value of 1000 in MEGA v11 (https://www.megasoftware.net/, accessed on 29 July 2023). The produced phylogenetic tree data were exported as a Newick file and further imported into iTOL (https://itol.embl.de, accessed on 29 July 2023) for visualization and modification.

### 2.5. Gene Structure and Conserved Motif Prediction

The genomic and coding sequences of *CqYAB* genes were directly input into Gene Structure Display Server 2.0 for gene structure analysis. Ten motifs were predicted in MEME (https://meme-suite.org/meme/tools/meme, accessed on 30 July 2023) using a zero-order model of sequences and a width from 6 to 50 amino acids. The N-terminal and C-terminal sequences of CqYABs were aligned in T-Coffee (tcoffee.crg.eu/apps/tcoffee/do:regular, accessed on 30 July 2023) and further displayed and viewed in NCBI Multiple Sequence Alignment Viewer for the conserved C2C2 zinc finger and YABBY domains.

### 2.6. Promoter Analysis

The 3 kb region upstream of each *CqYAB* gene was obtained from Chenopodium DB (www.cbrc.kaust.edu.sa, accessed on 27 July 2023). *Cis*-regulatory elements were analyzed using PlantCARE (https://bioinformatics.psb.ugent.be/webtools/plantcare/html/, accessed on 30 July 2023), and the number of elements was counted manually and represented in Microsoft Excel (Microsoft Corp., Redmond, WA, USA) with conditional formatting and color scale.

### 2.7. Isolation of RNA and qRT-PCR

The total RNA was isolated from collected samples using RNAiso Plus reagent (TaKaRa, Tokyo, Japan), and 1.0 μg of total RNA was used for first-strand cDNA synthesis with RNA HiScript^®^ III RT SuperMix for qPCR (+gDNA wiper) Kit (Vazyme, Nanjing, China). Quantitative real-time polymerase chain reaction (qRT-PCR) was performed using a Roche LightCycler 480 II real-time PCR system with ChamQ^TM^ Universal SYBR qPCR Master Mix (Promega, Beijing, China). The total volume of qRT-PCR was 20 µL, and it contained 10 µL of 2× SYBR qPCR Master Mix, 0.4 µL of primers (10 µM), 8 µL of cDNA templates (50 ng/µL), and 1.2 µL of ddH_2_O. The reaction was conducted under the following thermal cycling conditions: 94 °C for 2 min, followed by 40 cycles of 94 °C for 15 s, and 60 °C for 31 s. *CqACT2* was used as an internal control. The experiments were performed with three biological replicates. The relative expression levels of genes were calculated using the 2^−∆∆CT^ method. The primer sequences used are listed in Table S2.

### 2.8. Vector Construction, Plant Transformation, and Imaging

A 2022 bp *INO* promoter region from *A. thaliana* was amplified using PCR and inserted into pFGC5941. The coding region sequences of *INO*, *CqYAB4*, and *CqYAB10* were PCR amplified and inserted into pFGC5941 after the *INO* promoter for sequentially generating *pINO:INO*, *pINO:CqYAB4*, and *pINO:CqYAB10* constructs. The sequencing-confirmed constructs were transformed into *Agrobacterium* strain GV3101. In total, 3–4 solid plates were prepared for the transformed *Agrobacterium* and placed in a 28 °C incubator for two days. All clones on the plates were scraped into 100 mL infiltration medium and shaken for 20–30 min. Five-week-old *ino* mutants (SALK_026925) were used for dipping.

Images were taken using a stereomicroscope (Leica M205C; Leica Microsystems, Mannheim, Germany). Silique lengths were measured in Image J. The column charts were generated in GraphPad Prism 8 (GraphPad Software, San Diego, CA, USA). The number of siliques counted in each genotype was 24–30 (from six plants).

### 2.9. Statistical Analysis of Data

Statistical data were analyzed through one-way analysis of variance followed by Tukey’s honestly significant difference test with SPSS v16.0 (SPSS Inc., Chicago, IL, USA). All data are presented as mean ± standard error, and statistical significance was considered at *p* < 0.05 (* *p* < 0.05, ** *p* < 0.01, *** *p* < 0.001). 

## 3. Results

### 3.1. Chromosomal Location and Molecular Features of Quinoa *YABBY* Genes

**Figure 1:**
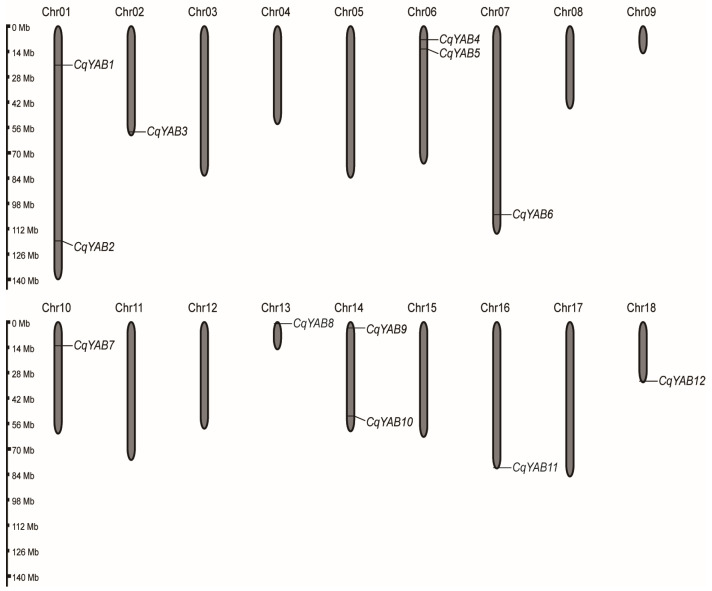
Chromosomal distribution of *CqYAB* genes. Chromosome numbers are shown at the top of each chromosome. The scale bar represents chromosome size.

A total of 12 *YABBY* genes were identified in the quinoa genome, and they were unevenly distributed on 9 of 18 chromosomes. These *YABBY* genes were designated as *CqYAB1* to *CqYAB12* (short for *CqYABBY1* to *CqYABBY12*) based on the order of appearance in chromosomes (Figure 1). *CqYAB1* and *CqYAB2* were located separately on the upper and lower arms of chromosome 1. *CqYAB3* appeared at the lower end of chromosome 2. *CqYAB4* and *CqYAB5* were mapped to the upper part of chromosome 6 and *CqYAB6* to the lower part of chromosome 7. *CqYAB7* was present on the upper arm of chromosome 10, and *CqYAB8* was located at the upper end of chromosome 13. *CqYAB9* and *CqYAB10* were found on the upper and lower arms of chromosome 14, respectively, and *CqYAB11* and *CqYAB12* were located separately on the lower ends of chromosomes 16 and 18 (Figure 1). *CqYAB* genes had six or seven exons with coding DNA sequences from 558 bp (*CqYAB5*) to 810 bp (*CqYAB2*), encoding proteins containing 185–269 amino acids (Table 1). The molecular weights of these proteins ranged from 20.51 to 29.53 kDa, and their pI values were from 4.90 (CqYAB10) to 9.40 (CqYAB8). The subcellular localization of all CqYAB proteins was predicted to be nuclear, with a probability in extracellular regions for CqYAB1 and CqYAB3 and in chloroplasts for CqYAB4 and CqYAB10 (Table 1).

### 3.2. Phylogenetic Analysis of *YABBY* Family Genes

**Figure 2:**
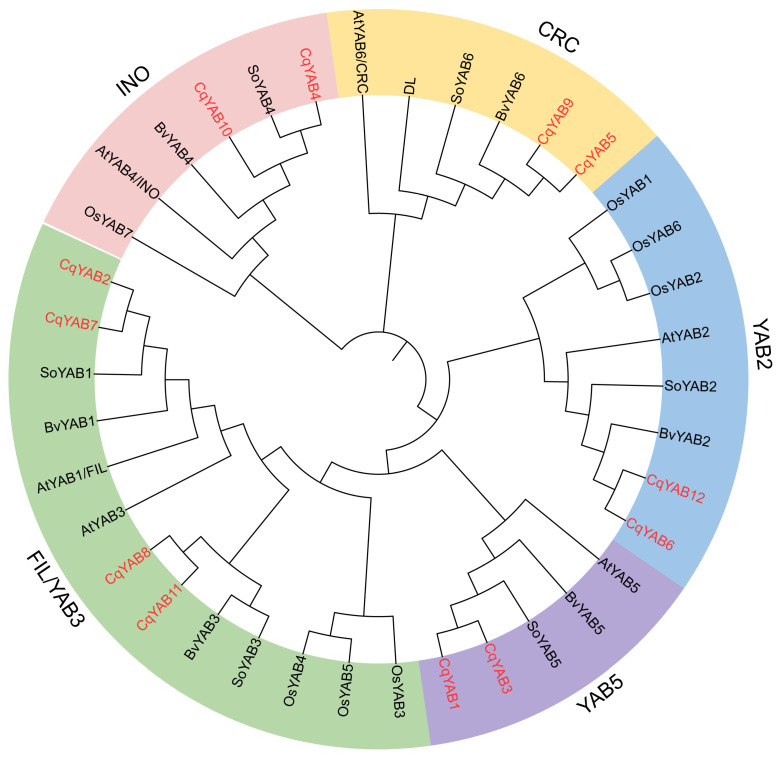
Phylogenetic tree of the YABBY family proteins from *Arabidopsis*, quinoa, spinach, beet, and rice. Gene names in red represent YABBYs from the quinoa genome. The five clades are indicated by differently colored backgrounds. At, *Arabidopsis* (*Arabidopsis thaliana*); Cq, quinoa (*Chenopodium quinoa*); So, spinach (*Spinacia oleracea*); Bv, beetroot (*Beta vulgaris*); and Os, rice (*Oryza sativa*).

To provide an overview of the phylogenetic relationships between *YABBY* genes, a phylogenetic tree was constructed for these *YABBY* genes from five species, including *C. quinoa*, *S. oleracea*, *B. vulgaris*, *A. thaliana*, and *O. sativa* (Table 1 and Table S1, and Supplementary File S1). The tree was divided into five clades, and at least two *CqYAB* genes were found in each clade (Figure 2). The largest group was the FIL/YAB3 clade, consisting of four CqYABs (CqYAB2, CqYAB7, CqYAB8, and CqYAB11), three rice YABs (OsYAB3, OsYAB4, and OsYAB5), and two YABs from *A. thaliana* (AtFIL/YAB1 and AtYAB3), *S. oleracea* (SoYAB1 and SoYAB3), and *B. vulgaris* (BvYAB1 and BvYAB3). In the YAB2 clade, there were three rice YABs (OsYAB1, OsYAB2, and OsYAB6), two CqYABs (CqYAB6 and CqYAB12), and one YAB in *A. thaliana* (AtYAB2), *S. oleracea* (SoYAB2), and *B. vulgaris* (BvYAB2). The INO clade contained two CqYABs from quinoa (CqYAB4 and CqYAB10) and one YAB from each of the remaining four species (AtINO, SoYAB4, BvYAB4, and OsYAB7). The CRC clade comprised two CqYABs (CqYAB5 and CqYAB9) and one from each of the other species (AtCRC, SoYAB6, BvYAB6, and DL). The YAB5 clade was the smallest, in which two CqYABs (CqYAB1 and CqYAB3) and one YAB each from *A. thaliana* (AtYAB5), *S. oleracea* (SoYAB5), and *B. vulgaris* (BvYAB5) appeared, but no rice ortholog was present (Figure 2). These data indicate that *YAB* genes in *C. quinoa* are conserved with those in other eudicots.

There were at least two paralogs from the quinoa genome in each clade of the tree, but the orthology in the clade FIL/YAB3 was not clear. 

### 3.3. Gene Structure, Protein Motif, and Domain Analyses

**Figure 3:**
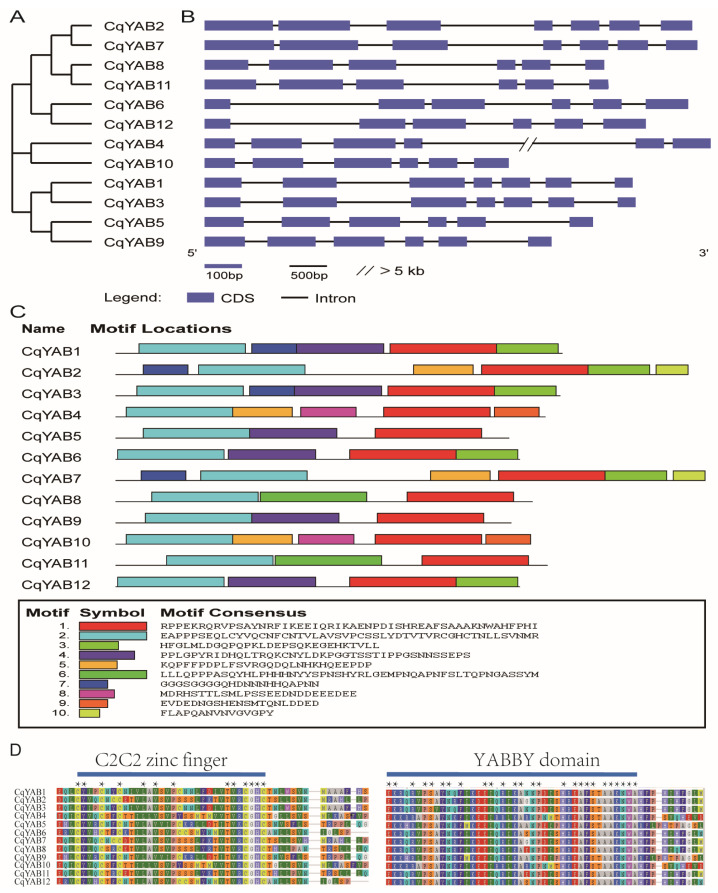
Phylogenetic relationships, gene structures, protein motifs, and domains of CqYABs. (**A**) Phylogenetic tree constructed through the neighbor-joining method. (**B**) Gene structures of *CqYAB* genes. Blue boxes indicate exons, and black lines indicate introns. The scale bar represents the length of exon or intron. The double slash depicts an intron length longer than 5 kb. (**C**) Motif composition of CqYAB proteins. Ten motifs were organized, each with a different color. (**D**) Conserved N-terminal C2C2 zinc finger and C-terminal YABBY domains of CqYAB proteins. Stars indicate the conserved amino acids in the domains.

The phylogenetic tree of the 12 *CqYAB* genes contained six paralogous pairs (Figure 3A). Each paralogous pair showed a similar gene structure with the same number of exons or introns. For example, *CqYAB2* and *CqYAB7* consisted of seven exons and six introns, while the *CqYAB8* and *CqYAB11* pair had six exons and five introns. In particular, the length of exon or intron in the same position within the gene pair was comparable, with the exception of the *CqYAB4* and *CqYAB10* pair, in which *CqYAB4* had a large fourth intron of more than 10 kb, although both genes contained six exons and five introns (Figure 3B). In addition, the protein motif prediction revealed that the same pair of CqYABs showed a similar number and organization of motifs (Figure 3C). For example, five motifs were found in the CqYAB1 and CqYAB3 pair, and the arrangement or order of the motifs in both proteins was the same. In the CqYAB8 and CqYAB11 pair and the CqYAB5 and CqYAB9 pair, only three motifs were found in each protein. The maximum number of motifs was found in the CqYAB2 and CqYAB7 pair, with six motifs in each protein. Motifs 1 and 2 were conserved in all 12 CqYAB proteins, representing more or less the C-terminal YABBY domain and the N-terminal C2C2 zinc finger motif, a feature of the YABBY family proteins (Figure 3D). 

### 3.4. Analysis of Cis-Regulatory Elements in *CqYAB* Promoters

**Figure 4:**
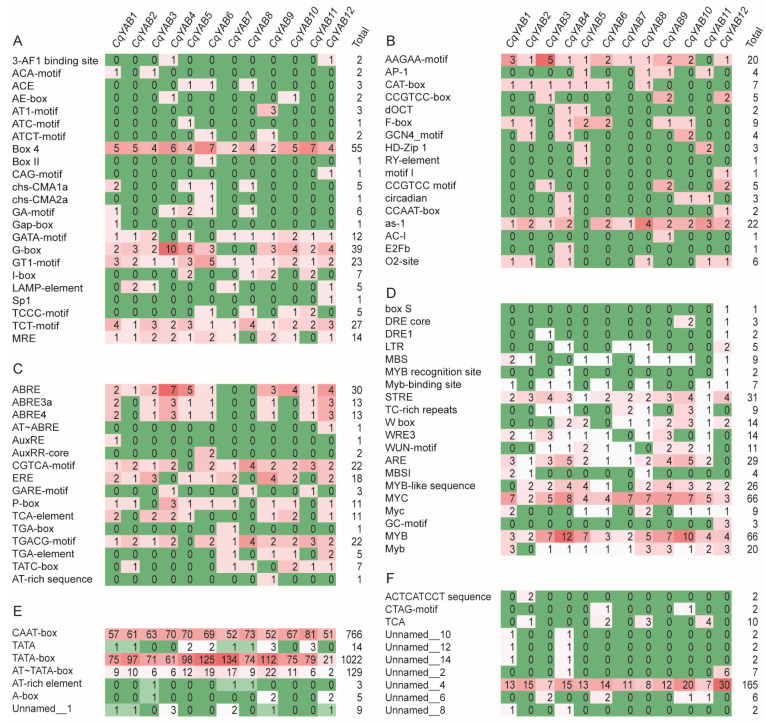
*Cis*-regulatory elements in the 3 kb promoter regions of *CqYAB* genes. The identified *cis*-elements were classified into six types. (**A**) Light-response elements. (**B**) Elements related to plant growth and development. (**C**) Phytohormone-response elements. (**D**) Stress-response elements. (**E**) Promoter-related elements. (**F**) Elements with unknown function. The names of *cis*-elements are listed on the left, and the numbers in different colors indicate the number of elements.

To reveal the potential functions of *CqYAB* genes, *cis*-regulatory elements of a 3 kb promoter from each *CqYAB* gene were investigated. A total of 93 *cis*-regulatory elements were identified in the 12 *CqYAB* genes, which could be classified into 6 types (Figure 4). Type 1 had 23 light-response elements, including box 4, GATA-motif, G-box, GT1-motif, TCT-motif, and MRE. Box 4 appeared in all 12 *CqYAB* promoters, with copies ranging from 2 (*CqYAB7* and *CqYAB9*) to 7 (*CqYAB6* and *CqYAB11*) in each promoter. The TCT-motif was also found in all 12 *CqYAB* promoters with at least 1 copy (*CqYAB2*, *CqYAB6*, *CqYAB7*, and *CqYAB9*) to 4 copies (*CqYAB1* and *CqYAB8*). Similarly, for the GT1-motif, half of the 12 *CqYABs* had only 1 copy, and the other half had 2–5 copies. The G-box was found in most *CqYAB* promoters, with the exception of *CqYAB7* and *CqYAB8*. In particular, *CqYAB4* contained 10 copies of the G-box, while *CqYAB5* had 6 copies. In addition, some light-response elements appeared only in one or two *CqYAB* genes, such as Sp1, which was specific to *CqYAB12*, and ACA-motifs were only found in *CqYAB1* and *CqYAB3* (Figure 4A). Type 2 included 17 development-related elements, such as AAGAA-motif, as-1, CAT-box (meristem), F-box and AP-1, GCN4_motif (endosperm), and RY-element (seed-specific). The AAGAA-motif was found in all but *CqYAB11* promoters. As-1 was also found in all but *CqYAB5*. The GCN4_motif was only detected in *CqYAB2*, *CqYAB4*, and *CqYAB10*, and the RY-element was detected only in *CqYAB5* (Figure 4B). Type 3 comprised phytohormone-response elements and consisted of 16 elements. It mainly included the ABA-response elements (ABRE, ABRE3a, ABRE4, and AT~ABRE), auxin-response elements (AuxRE, AuxRR-core, TGA-box, and TGA-element), gibberellin-response elements (GARE-motif, P-box, and TATC-box), MeJA- and salicylic-acid-response elements (CGTCA-motif, TGACG-motif, and TCA-element), and ethylene-response element (ERE). The TGACG-motif was found in all but *CqYAB5* promoters. ABRE was also detected in all *CqYABs*, with the exception of *CqYAB7* and *CqYAB8*. These hormone-response elements appeared in one or a few promoters; for instance, the TGA-box was only found in *CqYAB7* (Figure 4C). Type 4 contained 20 different stress-response elements, including drought-response element (MYB), dehydration-response element (MYC), and their related elements. MYB and MYC appeared in all 12 *CqYAB* promoters, with 66 copies in each, representing 39.9% of the total number of element copies detected in this group. The stress-related element (STRE) and antioxidant-response element (ARE) followed, with 31 and 29 copies, respectively. The former was found in all *CqYAB* promoters, but the latter was not detected in *CqYAB12*. The low-temperature-response element (LTR) was discovered in four *CqYABs* (*CqYAB4*, *CqYAB7*, *CqYAB8*, and *CqYAB12*), and WRKY TF binding sequence (W-box), WRE3, and WUN-motifs were found in most *CqYAB* promoters (Figure 4D). Type 5 included seven promoter-related elements, and the CAAT-box and TATA-box were the ones with the highest number in each *CqYAB* gene (Figure 4E). The last type consisted of 10 elements with unknown functions. Intriguingly, unnamed_4 appeared in all *CqYABs* with a copy number from 7 (*CqYAB3* and *CqYAB11*) to 30 (*CqYAB12*) in each promoter (Figure 4F). Taken together, these results indicate possible roles of *CqYABs* in light and hormone response, plant growth and development, and stress response.

### 3.5. Expression Responses of *CqYAB* Genes under Salt, Drought, and Cd Stresses

**Figure 5:**
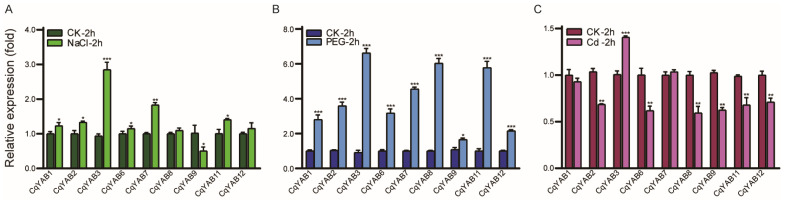
Expression responses of *CqYAB* genes under salt, drought, and Cd treatments. Two-week-old seedlings were treated with three stresses for 2 h and sampled for qRT-PCR. (**A**) Treatment with 200 mM NaCl. (**B**) Treatment with 15% PEG6000. (**C**) Treatment with 100 µM CdCl_2_ (Cd). The relative expression levels of genes were calculated using the 2^−∆∆CT^ method. The expression level of the control (CK) was arbitrarily set to 1. Error bars indicate standard deviations of the mean value from three biological replicates and three technical replicates. Asterisks indicate significant differences in the expression between control and stress samples (* *p* < 0.05; ** *p* < 0.01; and *** *p* < 0.001).

Following the promoter analysis, the expression responses of all *CqYAB* genes with treatments of salt (NaCl), drought (PEG), and Cd were investigated. Three *CqYAB* genes (*CqYAB4*, *CqYAB5*, and *CqYAB10*) had no detectable signal under these treatments. The remaining nine *CqYAB* genes showed various responses (Figure 5). In the salt treatment, the expression of six *CqYAB* genes was significantly upregulated. Among these, *CqYAB3* and *CqYAB7* showed a large change in expression, which increased by 2.7- and 1.6-fold, respectively. While *CqYAB9* expression was downregulated 1.0-fold, *CqYAB8* and *CqYAB12* displayed no significant change (Figure 5A). Under drought treatment, the expression of all nine *CqYAB* genes was significantly upregulated (Figure 5B). The first three *CqYABs* with a large change in expression were *CqYAB3*, *CqYAB8*, and *CqYAB11*, which increased by 4.0-, 5.0-, and 6.0-fold, respectively. The followers were *CqYAB7*, *CqYAB2*, *CqYAB6*, and *CqYAB1*, which were upregulated by 4.0-, 3.5-, 3.3-, and 3.0-fold, respectively. The last two were *CqYAB9* and *CqYAB12*, which increased only by 1.4- and 1.9-fold (Figure 5B). In contrast, the expression of six *CqYAB* genes was significantly downregulated under Cd treatment. The expression changes of these *CqYAB* genes ranged from 1.0-fold to 1.2-fold. *CqYAB3* was the only one with upregulated expression (1.4-fold) under Cd stress. The remaining two, *CqYAB1* and *CqYAB7*, showed no significant expression change under Cd treatment. In comparison, *CqYAB3* was the only one with a large increase in expression in response to the three stress treatments. These findings indicate that these *CqYAB* genes respond to two or three types of stresses, which is consistent with the promoter analysis data for these *CqYAB* genes (Figure 4D).

### 3.6. Tissue Expression Profiles of *CqYAB* Genes

**Figure 6:**
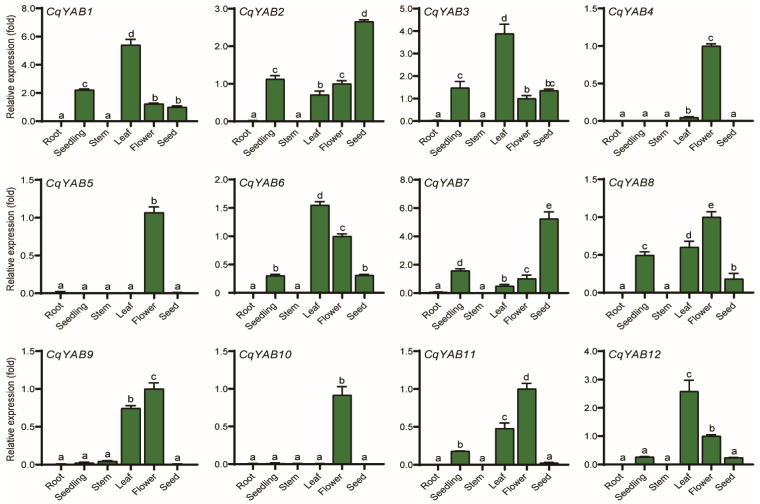
Expression patterns of *CqYAB* genes under normal growth conditions. The expression levels of the 12 *CqYAB* genes were determined in different tissues at different developmental stages through qRT-PCR, and a fold change in the expression level was shown for each *CqYAB*. The expression level in flowers was arbitrarily set to 1. *CqACTIN2* was used as an internal control. Different letters indicate significant differences in expression levels.

The expression of 12 *CqYAB* genes in several tissues or organs was determined by qRT-PCR. No detectable signal was found in the roots for any of the 12 *CqYAB* genes. Most paralogous pairs of *CqYAB* genes exhibited a similar expression pattern, such as the pair of *CqYAB1* and *CqYAB3* (belonging to the YAB5 clade), which displayed the highest expression level in leaves, followed by seedlings, with a significant and comparable expression level in both flowers and seeds (Figure 6). A high expression level in leaves was also found in *CqYAB6* and *CqYAB12*, two genes in the YAB2 clade (Figure 2 and Figure 5), with a moderate expression level in flowers for these two genes. *CqYAB2* and *CqYAB7* (FIL/YAB3 clade) revealed a high expression level in seeds and a moderate to low expression level in seedlings, leaves, and flowers. Another pair of this clade, *CqYAB8* and *CqYAB11*, had a high expression level in flowers, a moderate level in leaves, and a low level in seedlings (Figure 6). These eight *CqYABs* were vegetative genes primarily expressed in the leaf, seedling, flower, and seed. The remaining four *CqYAB* genes were reproductive ones; those, such as *CqYAB4* and *CqYAB10* (INO clade), were especially expressed in flowers, with a low expression level in leaves for *CqYAB4*. In the case of *CqYAB5* and *CqYAB9* (CRC clade), a flower-specific expression pattern was revealed for *CqYAB5*, but *CqYAB9* showed a moderate expression level in leaves, a detectable level in seedlings and stems, and a high expression level in flowers (Figure 6). Based on these expression data, the functions of these *CqYAB* genes in the seedling, leaf, flower, and seed/embryo were inferred. 

### 3.7. Functional Analysis of CqYAB4 and CqYAB10 in Arabidopsis

**Figure 7:**
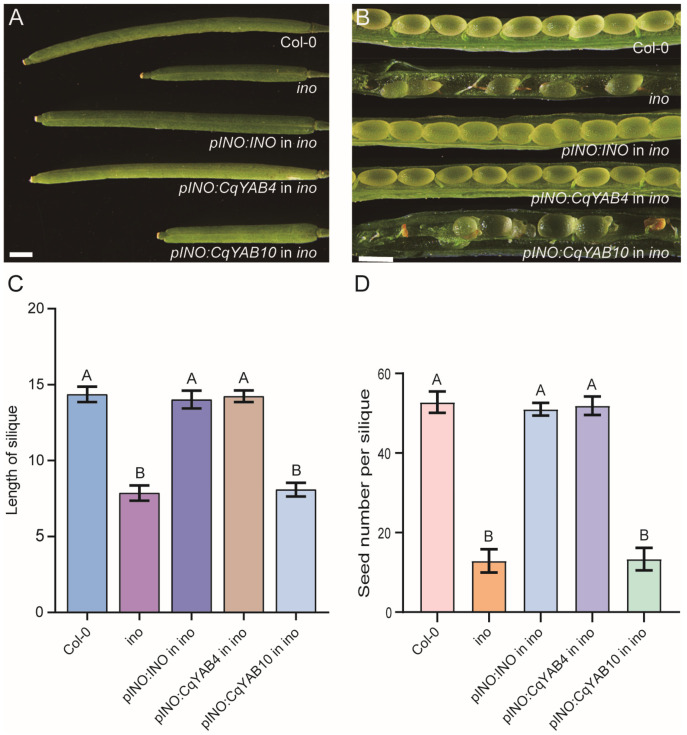
Complementation of the *ino* mutant phenotype by *CqYAB4*. (**A**) Silique morphology of different genotypes. (**B**) Dissected siliques in different genotypes, showing the developing seeds in siliques. (**C**) Silique length of different genotypes. (**D**) Seed number per silique of different genotypes. Capital letters indicate a significant difference (*p* < 0.01). The scale bar in the top left panel is 1 mm, whereas that in the top right panel is 0.5 mm.

In the phylogenetic tree, CqYAB4 and CqYAB10 were classified in the INO clade (Figure 2). To investigate whether CqYAB4 or CqYAB10 has the INO role in *Arabidopsis*, we generated transgenic plants and expressed the coding sequences of *INO*, *CqYAB4*, and *CqYAB10* under the *INO* promoter in the *ino* mutant background. *INO* and *CqYAB4* transgenic plants could fully rescue the *ino* mutant phenotype. Upon comparing the silique length and seed number of these transgenic plants with those of Col-0 and *ino* mutant, the *ino* mutant produced 8 mm siliques with 13 seeds per silique, whereas the *INO* and *CqYAB4* transgenic plants, as well as the Col-0 plants, produced 14 mm siliques with over 51 seeds per silique. However, this was not the case for *CqYAB10*, which could not rescue the *ino* mutant phenotype. The *pINO:CqYAB10* transgenic plants continued to form 8 mm siliques with 13 seeds (Figure 7A–D). The data suggest that CqYAB4 is the INO ortholog in quinoa, possibly controlling the patterning of the outer integument of the ovule.

## 4. Discussion

As members of a TF family, *YABBY* genes have been investigated in many species; for example, six members have been found in *A. thaliana* [56,57], eight in rice [37,40], nine in tomato [58], thirteen in maize [59], twenty-one in wheat [38,39], seventeen in soybean [52], seven in grapevine (*Vitis vinifera*) [60], twelve in *Gossypium arboreum* [55], and ten in *Juglans regia* [61]. In this study, we identified 12 *CqYAB* genes in quinoa. The varied number of *YABBY* genes in different species might be related to gene deletion, duplication, or genome hybridization. Quinoa is an allotetraploid (AABB, 2n = 4x = 36) likely derived from a cross between two diploids (AA and BB). The twelve *CqYABs* were distributed on nine chromosomes, with six on five A-chromosomes and six on four B-chromosomes (Figure 1) [16]. However, only three *YABBY* genes were discovered in the A-genome diploid *C. pallidicaule* (2n = 2x = 18) and the B-genome diploid *C. suecicum* (2n = 2x = 18) (Supplementary File S4). Thus, some of the twelve *CqYAB* genes might have been generated by gene duplication or another A- or B-genome diploid containing six *YABBY*s.

These YABBYs were usually divided into five clades—FIL/YAB3, YAB2, YAB5, INO, and CRC (Figure 2). The members of the five groups have already been found in *Cabomba caroliniana* and *Amborella trichopoda*, two members of the earliest diverging angiosperms [30,62]. However, the YAB5 group was missed in the evolutionary history of monocotyledons (Figure 2) [55]. The *YABBY* genes are also present in gymnosperms, in which they are divided into four clades [63]. Analysis of the phylogenetic relationships of the *YABBY* genes from angiosperms and gymnosperms suggested that the *YABBY* genes were already present in one common ancestor of the extant seed plants [63]. Based on current data, the origin of *YABBY* genes appears to coincide with leaf evolution in seed plants, which are important regulators for transforming the shoot meristem into a flat leaf [36,64]. However, tracing the origin of the *YABBY* genes in non-seed plants is difficult. Although we found a few *YABBY* sequences in clubmosses (*Huperzia selago* and *Diphasiastrum complanatum* in Lycopodiaceae) and in unicellular green algae *Micromonas commoda*, *Micromonas pusilla* CCMP1545, and *Chloropicon primus* (Supplementary File S5) by searching available databases (NCBI), they were not found in other mosses or lycophytes [63]. Intriguingly, the *YABBY* sequences from clubmosses contained seven exons and six introns, which are similar to the *YABBY* genes from *Arabidopsis* and other eudicots [65], while this was not the case for *Micromonas YABBY*s, in which only one exon is present (NCBI). These data imply that the *YABBY* genes occurred in a green picoplankton but were lost in other green algae, were later regained in some vascular plants, like clubmosses, with a changed gene structure, and were eventually maintained in seed plants. The functions of *YABBY* genes need to be investigated in non-seed plants using a state-of-the-art gene editing tool to determine whether they play roles in the development and defense processes or stress responses.

*YABBY* genes exert multiple functions in eudicots. One of the most important is to regulate leaf formation, which includes promoting lamina expansion, adaxial–abaxial polarity specification, marginal growth, and, finally, leaf shape determination [56,57,66]. The vegetative *YABBY* genes contribute much to leaf growth and development [36]. In quinoa, four pairs of *CqYAB* genes belong to the vegetative group genes. *CqYAB1/3* (YAB5 clade) and *CqYAB6/12* (YAB2 clade) showed the highest expression levels in leaves, whereas *CqYAB8/11* and *CqYAB2/7* (FIL/YAB3 clade) displayed moderate and low expression levels in leaves, respectively, suggesting that these *CqYAB* genes play a role in leaf formation (Figure 2 and Figure 6). Strikingly, *CqYAB8/11* and *CqYAB2/7*, however, exhibited the highest expression levels in flowers and seeds, respectively, suggesting that they play a major role in flower development and seed maturation (Figure 6). It is plausible that *YABBY* genes retain a role in flower development because floral organs are modified leaves [67]. The reproductive *YABBY* genes are specifically involved in flower organ formation; for example, *CRC* is involved in controlling carpel development [34] and *INO* is involved in ovule outer integument formation [35]. Consistent with these roles, *CqYAB5/9* and *CqYAB4/10*, the putative orthologs of *CRC* and *INO* in quinoa, displayed the highest expression level in flowers, and, in particular, the expression of *CqYAB5* and *CqYAB10* was restricted to flowers. To assess the function of *CqYAB4/10* in *Arabidopsis*, a T-DNA mutant *ino* was used to perform a complementation experiment. Surprisingly, only *CqYAB4* could rescue the *ino* mutant phenotype, but not *CqYAB10*, although their encoded proteins possess 96% similarity. This result shows that *CqYAB4* and *INO* were functionally conserved, whereas *CqYAB4/10* have diversified functions, at least in ovule growth and development, and future studies could be performed to find which amino acid is essential for INO function.

In addition to the functions in leaves and flowers, *YABBY* genes have an essential role in abiotic stress responses. For example, overexpression of soybean *GmYABBY10* and pineapple *AcYABBY4* in *Arabidopsis* led to sensitivity to salt stress [52,53], indicating a negative regulatory role for *GmYABBY10* and *AcYABBY4* in plant tolerance to salt stress. In addition, expression profile analyses showed that the *YABBY* genes responded to cold, heat, salt, and drought stresses in stiff brome (*Brachypodium distachyon*) [68], wheat [39], pak-choi (*Brassica rapa ssp. chinensis*) [69], common bean (*Phaseolus vulgaris*) [70], lotus (*Nelumbo nucifera*) [71], upland cotton (*Gossypium hirsutum*) [55], twelve stamen Melastoma (*Melastoma dodecandrum*) [72], and balloon flower (*Platycodon grandiflorus*) [73]. We assessed the 12 *CqYAB* genes under salt, drought, and Cd stresses, and found that most *CqYAB* genes were upregulated under salt and drought stresses but were downregulated under Cd stress. This was further supported by their promoter analysis, in which many stress-response elements were revealed, such as MYB, MYC, STRE, ARE, LTR, and W-box (Figure 4D). Same or similar *cis*-regulatory elements were also discovered in the *YABBY* genes from other species [68,72,74], suggesting that these *YABBY* genes have a conserved function in stress response; however, the underlying molecular mechanism remains elusive. Therefore, the determination of the *YABBY* downstream targets or interactors in the stress response pathway could help improve the defensive traits in quinoa and other crops.

## 5. Conclusions

Twelve *CqYAB* genes were identified in the quinoa genome, and they were divided into five groups. Tissue expression profiles showed that these *CqYAB* genes were primarily expressed in leaves, flowers, and seeds, and most *CqYAB* genes responded to salt, drought, and Cd stresses, suggesting that the *CqYAB* genes play roles in both development and abiotic stress responses. *CqYAB4* could rescue the *ino* mutant phenotype but not *CqYAB10*, another member in the INO group, which is indicative of functional conservation and divergence among these *YABBY* genes. The results from this study provide a fundamental basis for further analysis of the functions of *CqYAB* genes and the underlying molecular mechanisms and add new resources for agronomic trait breeding.in quinoa.

## Figures and Tables

**Table 1:** Molecular features of identified *YABBY* genes in the quinoa genome.

Gene Name	Gene ID	CDS (bp)	Exon	Protein (aa)	MW (kDa)	pI	Subcellular Location
*CqYAB1*	XP_021760782.1	633	7	210	23.53	8.44	Nucleus/extracellular
*CqYAB2*	XP_021748608.1	810	7	269	29.53	7.78	Nucleus
*CqYAB3*	XP_021741749.1	630	7	209	23.51	8.44	Nucleus/extracellular
*CqYAB4*	XP_021727415.1	609	6	202	23.15	4.67	Nucleus/chloroplast
*CqYAB5*	XP_021729181.1	558	6	185	20.51	8.76	Nucleus
*CqYAB6*	XP_021720585.1	573	6	190	21.23	7.64	Nucleus
*CqYAB7*	XP_021756313.1	834	7	277	30.44	7.39	Nucleus
*CqYAB8*	AUR62007472	591	6	196	21.94	9.40	Nucleus
*CqYAB9*	XP_021717873.1	561	6	186	20.73	8.76	Nucleus
*CqYAB10*	XP_021742940.1	588	6	195	22.25	4.90	Nucleus/chloroplast
*CqYAB11*	AUR62039930	612	6	203	22.71	9.37	Nucleus
*CqYAB12*	XP_021715646.1	573	6	190	21.21	7.66	Nucleus

pI: Theoretical isoelectric point; MW: Molecular weight.

## Data Availability

Data are contained within the article and Supplementary Materials.

## Supplementary Materials

The following supporting information can be downloaded at: https://www.mdpi.com/article/10.3390/genes14112103/s1. Supplementary File S1: Protein sequences used in generating the phylogenetic tree in Figure 2; Supplementary File S2: Promoter sequences; Supplementary File S3: Genomic sequences and coding region sequences of *CqYAB* genes; Supplementary File S4: YABBY protein sequences in *C. pallidicaule* (A-genome) and in *C. suecicum* (B-genome); Supplementary File S5: YABBY-related sequences from clubmosses and *Micromonas* species; Table S1: List of *YABBY* genes in *Spinacia oleracea, Beta vulgaris, Arabidopsis thaliana,* and *Oryza sativa*; Table S2: List of primers used in this study.
