# Supplementary material for: Molecular Characterization and Expression Analysis of YABBY Genes in Chenopodium quinoa
Source: Genes (Basel). 2023 Nov 19;14(11):2103. doi: 10.3390/genes14112103 (PMC10671189; doi:10.3390/genes14112103)
Supplement: Supplementary file 1 [file genes-14-02103-s001.zip › genes-2679335-SI.pdf]

Supplementary File S1
Protein sequences used in generating phylogenetic tree in Figure 2

>AtYAB1/FIL
MSMSSMSSPSSAVCSPDHFSPSDHLCYVQCNFCQTILAVNVPYTSLFKTVTVRCGCCTNLLSVNMRSY
VLPASNQLQLQLGPHSYFNPQDILEELRDAPSNMNMMMMNQHPTMNDIPSFMDLHQQHEIPKAPP
VNRPPEKRQRVPSAYNRFIKEEIQRIKAGNPDISHREAFSAAAKNWAHFPHIHFGLVPDNQPVKKTNM
PQQEGEDNMVMKEGFYAPAAANVGVTPY

>AtYAB2
MSVDFSSERVCYVHCSFCTTILAVSVPYASLFTLVTVRCGHCTNLLSLNIGVSLHQTSAPPIHQDLQPHR
QHTTSLVTRKDCASSSRSTNNLSENIDREAPRMPPIRPPEKRQRVPSAYNRFIKEEIQRIKACNPEISHRE
AFSTAAKNWAHFPHIHFGLKLDGNKKGKQLDQSVAGQKSNGYY

>AtYAB3
MSSMSMSSSSAPAFPPDHFSSTDQLCYVHCSFCDTVLAVSVPPSSLFKTVTVRCGHCSNLLSVTVSMRA
LLLPSVSNLGHSFLPPPPPPPPPNLLEEMRSGGQNINMNMMMSHHASAHHPNEHLVMATRNGRSVD
HLQEMPRPPPANRPPEKRQRVPSAYNRFIKEEIQRIKAGNPDISHREAFSAAAKNWAHFPHIHFGLMA
DHPPTKKANVRQQEGEDGMMGREGFYGSAANVGVAHN

>AtYAB4/INO
MTKLPNMTTTLNHLFDLPGQICHVQCGFCTTILLVSVPFTSLSMVVTVRCGHCTSLLSVNLMKASFIPL
HLLASLSHLDETGKEEVAATDGVEEEAWKVNQEKENSPTTLVSSSDNEDEDVSRVYQVVNKPPEKRQ
RAPSAYNCFIKEEIRRLKAQNPSMAHKEAFSLAAKNWAHFPPAHNKRAASDQCFCEEDNNAILPCN
VFEDHEESNNGFRERKAQRHSIWGKSPFE

>AtYAB5
MANSVMATEQLCYIPCNFCNIILAVNVPCSSLFDIVTVRCGHCTNLWSVNMAAALQSLSRPNFQATN
YAVPEYGSSSRSHTKIPSRISTRTITEQRIVNRPPEKRQRVPSAYNQFIKEEIQRIKANNPDISHREAFSTAA
KNWAHFPHIHFGLMLESNKQAKIA

>AtYAB6/CRC
MNLEEKPTMTASRASPQAEHLYYVRCSICNTILAVGIPLKRMLDTVTVKCGHCGNLSFLTTTPPLQGH
VSLTLQMQSFGGSDYKKGSSSSSSSSTSSDQPPSPSPPFVVKPPEKKQRLPSAYNRFMRDEIQRIKSANPEI
PHREAFSAAAKNWAKYIPNSPTSITSGGHNMIHGLGFGEKK

>CqYAB1 (C2C2 zinc finger underlined with black color; YABBY domain underlined with green color)
MSSTSCVDQAAVAPPSSEQLCYIPCNYCNIVLAVSVPCNNLFDIVTVRCGHCTNLWSVNMAAAFHSL
SASWQQHQQQNFHQAPNNGNMGEYRIDNLGSSSKCNYTSKAATTMRISPPISNNSSEERVINRPPEK
RQRVPSAYNQFIKEEIQRIKANNPDISHREAFSTAAKNWAHFPHIHFGLMLETNNQPKLDEGSQKHL
MPRTALLNN

>CqYAB2

MSSSSSSTTTGSGGGGCGGGGSLDNNYHHQTPNNNNNVEHVISPSDQLCYVQCNCCETVLAVSVPSS
SLFKTVTVRCGHCTSLLSVNMRAHLLPSPSPPSPLPPPSPNHLHLSPPSFFTSPHNLLEEIRSSTPNILINN
HHQPMFNDPMMSVRGVDHLHHHHQEIPKPPPVNRPPEKRQRVPSAYNRFIKDEIQRIKAGNPDISHR
EAFSAAAKNWAHFPHIHFGLMPDHQPVKKANVRQQQEGEHDQVMMKEGFLAPQANVNVGVGPY

>CqYAB3
MSSSCMDQAAVAPPSSEQLCYIPCNYCNIVLAVSVPCNNLFDIVTVRCGHCTNLWSVNMAAAFHSLS
ASWQQHQQQNFHQAPNNGNMGEYRIDNLGSSSKCNYTNKAATTMRISPPISNNSAEERIINRPPEKR
QRVPSVYNQFIKEEIQRIKANNPDISHREAFSTAAKNWAHFPHIHFGLMLETNNQPKLDEGSQKHLM
PRTALLNN

>CqYAB4 (Predicted NLS in red color)
MATLNRLFDTQEQICYVQCSFCTTILLVSVPYSSMTMVVTVRCGHCTGLLSVSMLKASFVPLHLFSALN
QDQVNFKHQEEVDTSKAHMDRHSTTLSMLPSSEEDNDDEEEDEEEDEDNIALEQIVNKPPEKKRRAP
SAYNKFIKEEIRRLKARNPNMTHKEAFSTAAKNWAHFPSVQHEVDEDNDSYENSMTQNLDDEDEAN

>CqYAB5
MNTNTMEDKVGSELAPPPPEHLCYVRCNFCNTVLAVVIPCKRLLDTITVKCGHCSNVSFLSTRPPLQG
QCLDHQITLQGFNFLEKPGGFCSTIDHHSNKKSEPSPSSSTLTEPVSPRPFVCKPPEKKHRLPSAYNRF
MKEEIQRIKAANPEIPHREAFSTAAKNWARFLPHTPAGSLAESSNPN

>CqYAB6
MEFSTSAERVCYVHCTFCKTILAVSVPCCSMYNMVTVRCGHCANLLSVNIGLSPPSMPHQDNFQLLR
QHCNYQDVSKDSSSTITGGSSSSTITTMASDDHDVQQTRPPPIRPPEKRQRVPSAYNKFIKEEIQRIKASN
PEISHREAFSAAAKNWAHFPHIHFGLNLDGQRQERLDQPVSGEGTNKSDGFY

>CqYAB7
MSSSSSSTTTGSGGGCGGGGSLDNNYHHQTPNYNNNDNVEHVISPSDQLCYVQCNCCDTVLAVSVP
SSSLFKTVTVRCGHCTSLLSVHMRAHLLPAVPSPSPPSPLPPPPPLSPNHHHLSPPSFFTSPHSLLEEIRSS
APNILINNHHHQPMFNDPMMSVRGVDHLHHHHQEIPKPPPVNRPPEKRQRVPSAYNRFIKDEIQRIK
AGNPDISHREAFSAAAKNWAHFPHIHFGLMPDHQPVKKANVRQQQEGEHDQVMMKEGFLAPQA
NVNVGVGPY

>CqYAB8
MSSSNTAASSTTTSLSLDHFSPSEQLCYLQCSRCETVLAVSVPSSSLYRTVTVRCGHCTHLLPANTRSLLL
QPPPASQYHLPHHHNYYSPNSHYRLGEMPNQAPNFSLTQPNGASSYMNPSSSRGGPNELPRAPTTNR
PPEKRQRVPSAYNRFIKEEIQRIKAENPDISHREAFSAAAKNWAHFPHIQFGLMPERR

>CqYAB9
MMNTNMMEDKVGSELAPPPPEHLCYVRCNFCNTVLAVVIPCKRLLDTITVKCGHCSNVSFLSTRPPL
QGQCLDHQITLQGFNFLEKPGGFCSTIDHHSNKKSEPSPSMSSTLTEPVSPRPFVCKPPEKKHRLPSAY
NRFMKEEIQRIKAANPEIPHREAFSTAAKNWARFLPHTPAGSLSESSNTN

>CqYAB10 (Predicted NLS in red color)

MATLNRLFDTQEQICYVQCSFCTTILLVSVPYSSMTMVVTVRCGHCTGLLSVNMLKASFVPLHLFSAL
NQDQVNFKHQEEVDTSKAMDRHSTTLSMLPSSEEDNDDEEEDDEDNIAIEQIVNKPPEKKRRAPSAY
NKFIKEEIRRLKARNPNMTHKEAFSTAAKNWAHFPSVQHEVDEDNGSHENSMTQNLDDED

>CqYAB11
MSSSNIAASSTTTTTTTTTTSLSLDHFHPSEQLCYLQCTRCETVLAVSVPSSSLYKTVTVRCGHCTHLLPV
NTRSLLLQPPPASQYHLPHHHNYYSPNSHYRLGEMPNQAPNFSLTQSNGASSYMNPSSSRGGSNELPR
APTTNRPPEKRQRVPSAYNRFIKEEIQRIKAENPDISHREAFSAAAKNWAHFPHIHFGLMPERR

>CqYAB12
MEFSTSAERVCYVHCTFCNTILAVSVPCCSMYNMVTVRCGHCANLLSVNIGLSPPSMPYQDNFQLLR
QHYNYQDVSRDSSSTITGGSSSSTITTIASDDHDVQQTRPPPIRPPEKRQRVPSAYNKFIKEEIQRIKASNP
EISHREAFSAAAKNWAHFPHIHFGLNLDGQSQARLDQPVSGEGTNKSHGFY

>BvYAB1
MSSSSSSTTTTGSAGGGGSLDTTTNHSHNTNVENHLLSPSDQLCYVHCNFCDTVLAVSVPSSSLFKTVT
VRCGHCTNLLSVQMWTPSTPPPPLPTNQLHHHLPPPSFFTSPHNLLEEIRSSTPNFINNHHQSVFNEP
MMPVRGIDHHHHQELPKPPPVNRPPEKRQRVPSAYNRFIKEEIQRIKAGNPDISHREAFSAAAKNWA
HFPHIHFGLMPDHQPVKKANVRQQQEGEHDVLMKEGFLAPQANCNVNVGVGPY

>BvYAB2
MDFSTSERVCYVHCTFCNTILAVSVPCCSTYNLVTVRCGHCANLLSVNIGLSPPSVPHQDNFQLVRQH
YNHQDESKDSSNITAGSSSSTITTVGSDQHDDHQQLRPPPIRPPEKRQRVPSAYNKFIKEEIQRIKASNP
EISHREAFSAAAKNWAHFPHIHFGLNLDGQRQARLDQAVAGEGANKSHGFY

>BvYAB3
MSSSTTTTNLSLDHFPPSEQLCYLQCTRCETVLAVSVPSCSLFKTVTVRCGHCTHLQPANTRSLLVQPPP
NQFHLAHNYYSPPTSHYRLGEMANPSPTYSVTQPNATSSYMTPTLSRGGSNELPKPPTTNRPPEKRQR
VPSAYNRFIKEEIQRIKAENPDISHREAFSAAAKNEAGEDNVRMRSGFYSAQTNVGVSPY

>BvYAB4
MATLNRLFDAQEQICYVQCNFCTTILLVSVPYNSMSMVVTVRCGHCTGLLSVNMLKASFVPLHLFSAL
NQDQLNKHQEEVDTSKVIMDRHSTTLSMLPSSEEDNDDEDDEEEEVDEDIIALEHTVNKPPEKKRRA
PSAYNRFIKEEIRRLKARYPNMSHKEAFSTAAKNWAHFPSVQHGVDEENGSHGNRHMTHNFEEEAG

>BvYAB5
MTNCVDQAVPSSEQLCYIPCNFCNIVLAVSVPCNNMFDIVTVRCGHCTNLWSVDMAAAFRSFASSW
QHHQQQNFHQGPNCGNTGEYKIDNLGSSSKCNYTNKATTMRISPPMNNNTSEERIINRPPEKRQRVP
SAYNQFIKEEIQRIKANNPDISHREAFSTAAKNWAHFPHIHFGLMLETNNQAKLDEGSQKHLMPRTA
LLNN

>BvYAB6
MSNNMEDKVSMEMAPPPEHLCYVRCNFCNTVLAVVIPCKRLLETVTVKCGHCGNVSFLSTRPPLQG
QCLDHQITLQGFNFMEKPGGFCSTVDHHSKKGQSSSSSSSTLTEPVSPRPYVVKPPEKKHRLPSAYNRF
MKEEIQRIKTANPEIPHREAFSTAAKNE

>SoYAB1
MSSSSSSTTGSGGGGSGFDTTPQTPTEHVVSDQLCYVHCEFCDTVLAVSVPSSSLFKTVTVRCGHCTNL
LSVNMMVHLLQAAAPSPPLLPPPPPLPHHLSHPSFFNPPHNNLLEEIRSSTPNILMNNHHQPMFNDQ
MMSIRGGDHLHHHHQEIPKPPPPVNRPPEKRQRVPSAYNRFIKDEIQRIKAGNPDISHREAFSAAAKN
WAHFPHIHFGLMPDHQPVKKANVRQQQEGEHDQVLMKEGGFLAPQANVNVGIGPY

>SoYAB2
MEFSTSERVCYVHCTFCNTILAVSVPCCSIYNLVTVRCGHCANLLSVNIGVSPPSVPNQENSFQHYSNY
QDVSKDSSTMTGGSSSSTITTMTSDQHDDQQSRLSPIRPPEKRQRVPSAYNKFIKEEIQRIKASNPEISHR
EAFSAAAKNWAHFPHIHFGLNLDLDGQRQARLDQAVAGIEGTNKSHGFY

>SoYAB3
MSSSTSSTITTNLSLDPFPSSEQLCYLQCSRCDTVLAVSVPSSSLYKTVTVRCGHCTHLLPVNTRSLLPPPP
PPNQLHLSHNFFSPTSHYRLGEMPNQSPNFSLTQPGGASNYMTPTLSRGGANELPRVPTTNRPPEKRQ
RVPSAYNRFIKDEIQRIKAQNPDITHREAFSAAAKNWAHFPHIHFGLMPDQTVKRTSNMRPHHPQEA
GEDVRMRTGYYGAQANVGVSPY

>SoYAB4
MAASLDRFFDTQEQICYVQCTFCTTILLVSVPTCSMSMVVTVRCGHCNGLLSVNMLKASFVPLHLFSS
LNQDQENNKHQEEVNMSRVTADRHSTTLSMLPSSEEDNDDGDDDEEEDDDDDDEDNIVLEQVVNK
QEIRRLKSRNSNMSHKEAFSTAAKNWAHFPRVQNEVEEQNDSHDNVKMTRHFEEETR

>SoYAB5
MSSCVDQAAAAVPSSEQLCYIPCNFCNIVLAVSVPCNNLFDIVTVRCGHCTNLWSVNMAAAFQSLTSS
WQQHHQQNFHQAPNNGNMGEYRIDNLGSSSSKCNYNHKAATTMRISPPLNNHLAEERIINRPPEKR
QRVPSAYNQFIKEEIQRIKANNPDISHREAFSTAAKNWAHFPHIHFGLMLETNNNQAKLDEGSQKHL
MPRTTALLNN

>SoYAB6
MMNRKMMEGKMSMELNAGPDHVCYVRCNLCNTVLAVVIPCKRLLETITVKCGHCGNVSFLSTRPLP
PLTATATATATATATATPTPTPLQPQGFNFLEKTCSSDANHSSSSNSTLTNDPHSPSPFVCKPPEKKHRLP
SAYNRFMKEEIQRIKAANPEIPHREAFSTAAKNWARFLPHSPSAGTNNTN

>OsYAB1
MSVQFTSEHVCYVNCNYCNTILVVNVPNNCSYNIVTVRCGHCTMVLSMDLAPFHQARTVQDHQVQ
NRGFQGNNFGSYDIASRNQRTSTAMYPMPTSQQQVSPIRPPEKRQRVPSAYNRFIKEEIQRIKTSNPEIS
HREAFSAAAKNWAHLPRLHFGLSVADGGGGGGSN

>OsYAB2
MSAQIVPAPEHVCYVHCNFCNTIFAVSVPSNSMLNIVTVRCGHCTSLLSVNLRGLVQALPAEDHLQD
NLKMHNMSFRENYSEYGSSSRYGRVPMMFSKNDTEHMLHVRPPEKRQRVPSAYNRFIKEEIRRIKAN
NPDISHREAFSTAAKNWAHFPNIHFGLGSHESSKKLDEAIGAPSPQKVQRLY

>OsYAB3
MSSSSSSSASSAAAAAFRPAVVQREQQVVEEKFPAAAAAMREMVLPPVAAAAADSEQEQLCYVHCH
YCDTVLVVSVPSSSLFETVTVRCGHCSSLLTVNMRGLLLPTTAAAAPPPPPPPPPPPPPPAAHFPHSLNL
APANPPHHHSLLDEISTANSPTQLLLEQHGLGGLMASAASCRNNNSPAAAAAPPPPTSQGKAAAKEP
SPRTNTAVINRPPEKRQRVPSAYNRFIKDEIQRIKAGNPDISHREAFSAAAKNWAHFPHIHFGLMPDH
QGLKKTSLLPQDHQRKDGLLKEGLYAAAAAAAAAAANMGVAPY

>OsYAB4
MSSSSSSSAVFPLDHLAAPSPTEQLCYVHCNCCDTILAVGVPCSSLFKTVTVRCGHCANLLSVNLRGLL
LPAPAPAPANQLHFGPSLLSPTSPHGLLDEVAFQTPSLLMEQAASASLSSITGRSSSSCASNAPAMQMPP
AKPVQQEPELPKNAPASANRPPEKRQRVPSAYNRFIKDEIQRIKAGNPDISHREAFSAAAKNWAHFPH
IHFGLMPDQGFKKTFKPQDGSEDILLKDSLYAAAAAAAAAAAANMGVTPF

>OsYAB5
MMSSAPETFSLDHLSQHQQQQPPPLAEQEQLCYVHCNFCDTILAVGVPCSSLFKTVTVRCGHCANLL
SVNLRGLLLPAAASTANQLPFGQALLSPTSPHGLLDEVPSFQAPASLMTEQASPNVSSITSSNSSCANN
APATSMASAANKATQREPQQPKNAPSANRTSEKRQRVPSAYNRFIKDEIQRIKASNPDITHREAFSAA
AKNWAHFPHIHFGLMPDQGLKKTGIQSQDGAGECMLFKDGLYAAAAAAAAATAASSMGVTPF

>OsYAB6
MSAQIAPAEQVCYVHCNFCNTILAVSVPGNSMLNIVTVRCGHCTNLLSVNLRGLMHSAPALQDHHH
HHLQESGLSGCFRDQSGYPEFGFSAASSSSKLRLPPAAAAMVSYSQQNQQLEQALHARPPEKRQRVPS
AYNRFIKEEIRRIKANNPDISHREAFSTAAKNWAHYPNIHFGLSPGHEGGKKLVDVDPIPTAPSSKKIQG
FYS

>OsYAB7
MSSAARHHCSGLRERLGCVQCSFCATVLLVSVPCSSVLRVVAVQCGHCSGILSAVNLPPSPVSASIELTP
QELDAGPPPGEYSDESSGDDREGRDAEDDAPAPAAAAVANKPPGRKQRTPSAYNCFVKEEIKRIKSME
PNITHKQAFSTAAKNWAHLPRIQQKRGRDSC

>DL
MDLVSPSEHLCYVRCTYCNTVLAVGVPCKRLMDTVTVKCGHCNNLSFLSPRPPMVQPLSPTDHPLGP
FQGPCTDCRRNQPLPLVSPTSNEGSPRAPFVVKPPEKKHRLPSAYNRFMREEIQRIKAAKPDIPHREAFS
MAAKNWAKCDPRCSSTVSTSNSNPEPRVVAAPIPHQERANEQVVESFDIFKQMERSG

Supplementary File S2
Promoter sequences

>*CqYAB1*
CTCTTTTAGTCTCTTATCTCCAAGAGAAATAGATAAGAGTAAGAAAAAAAGAATAAATTAGATCA
ATAGTTAGAATTCGGGTAATTAATGGAGTATAAATCACTTTTGGTAGGTACGTAAAACTTTTTATTA
ATCCAATTTGTTAATAAAATTATAAATTATAAATTGATACGAAATATAACTGATTTTTTTAAGTAAA
AAGGGTATCCCCACTTGTTGACATGACTAAACATAAACTTTCCAAATAACATAAGATCAAATCAC

AGTAAATTATAGCTCAATGTTATGCTAAAAATAGTATTCTTAGTTGTAAGATGGATTGCCTTTTACA
ATAATGCTACGTGAAAATTAAGTAAAATTTATTTCATTGGCTACTTATCACAACCATAATATATACG
GAGTACTAATGTTTGTGAACTGTCTCCATCACAATGGTAAAACTAAAGTTTTATTTTAAATTTTTTA
TTAATAATATTTTTTTTATTTATTTTTTAACGTTTATTTAATTTTTTTATTTTCTATTTTTTTTGGGGGGG
TGGGAGTGTTACGGACACAAACATGAAAACTAAAACTCAAATGAAAAAGTCAAGCTTTATTTTT
TCATGATCTGTTCTTTACATAAATTACAGAGTAGTGATATATGCATGTCTAATGGTTTTTTTTTGATA
CTTTAAAATTGTCTTATTCTGCTTATTCTCTAGAAAGTTTATGTTTTTTCAAAGGTGACTTGTTATAA
TTATATATTTATTGATTCAAAATTTAAATGTAGAAGCATCTATTATTAAACATTGAATAATATTTTTT
GTTGAGCTTGAAACACACTAACAATTAAGAAATTCAGCATATTACAAGTATATATGAATAATTTGT
TGTTGATTAATTGGAAGCCTTAAGCAAATTTTAGGGTACGGTATGATGACCTAGTTCTTCTTTTAA
ATAATTTAAACTAAGGGTCTGTTCTCATACGTTGAAATTCAATTCAGTTCAGTTCAGCTCCTAATTT
CCTTCTCTTATAAAATATTCAGTACAGTTCAGCTCAGTTCAGTTCAGTTCAGTTCAGTTCAGCTCA
ATTTAGTTCAGTTCAGCTCAGTTCAGTTCAGCTCAGTTCAGCTCAGTTCAGTTCAGTTAAGTTCAG
TTCAATTCAGCTGAGTTCAGTTAATGAGAACAGACCCTAATTTCTTCAAAAACAAACAAAAAAA
AATTAAACTAATTTAATGCTCTCAAAGTAGGAAAATAAGTATATATACGTACAATTAGTATCTCAT
CACTTCATTCCATAAATACACATTATTAGATTTCACTTTTTGTGAATGACTGTATTATTAATCCGTAA
AATCATCAACACTACATGAAAAAAAATAAAAAATAAAAAATTGTACTCTAACGAGGTATTTGTG
TTCCACCTAAAAATCGACCATAAACTTGAAGAAGCTTAAAGAGAAGAGACAATTCTTTATTGTA
ATTGGACTTGAAGTCTACAATGATAACATTATTAGACATAAATCATTACTCATTACACTGTTTAGTC
CCTCTAATGATTTCTTATTAAGACAAATGAGTCGATAACAATAATGATGTATGTGAAAGAAATGAT
GATCAATTGGTTCTTTCATTATGACATACATCTGCTCTAAAAAGATTTTGGACTTTTCTCCCATTCA
TATGGAACGAACTTAATTAAGGATCCAACTTCATGCTTTAGGTTAAAGGGATACATCTATTATTCA
TTTTTTTTGTTGATTATTTATTGATTAAAATGTGTGATCACATTTGTAATAAGGAGTAATGTGTTAAT
CAACACTTGTTTTTTCACCAAATTGTCCTATATATGGTATTAGGGTGGTGGGAGATACGCCTATTAC
ACACGGAAGCAATTGGGAAAATAAAAATTGAAAGTCCAAGCAATTCAATTAACCTCTACCATCG
AGTTCTTACTTGATTCTCTACGTGCACCTGCATTTGATGAGTGACGTATACGCAGTCATCATTATCA
TATATATTCATCTTTTCTTGCTTCACGAATATGTAATTAGCGCTCACACATACAAATCCCTTACATTA
CACAATGGTAGAATTTGATGAGAGATACATCTCATAAGTAACAACCACACCCAAGCAAATGACT
AAGCTAGAGCTAAAGTTTATACTATACTTGTATATAACATATCATTTTCGTTAAATCCATAAAGAA
AAGGAAGAAAAAAAAAGGAGAAAACATCCACAAAAATTCATAAACCTCATGATCTCATCCACCT
ATTGTATAGTGGCAAGGAAAGGGGTGAGAGGGACTACACACACAACCTTGTTTCATAATAATAG
CCAGCCATACTAACCTGTCCAATTTTATTAGTCCTGCCAAAATCATTCATATTTATGATTTACCCGT
TTTTTCTAACTCTTCAATTCACGATAATTTTATTTTCTCTAAACCATCCGGTCTGAACCAGCCTAAA
TATTCAAATCTCGAAAGAATTTCCTTTCTCAACCCCACCATTTTCTTGTTTGTAGCCTTCAACACC
ACTTCTTTTTTTTTATAACTTTGATTTTCATTTAAAAAAAAAATCACCCAACTTTTTTTTTTTTTTTTAA
ATAATTTTATTTTCTTCCCATCTTTATAGACATAAATAATTGAAGTTCATTTAACATTATTAGCCCTT
CCCTTCCCCTACCTAGTCTTTTCCTATCAATATTTCTCTCTCTTCCTCATCTTCCGGGCTATATTTCTC
CTTTTATTCAGAGAATTTCTGTTATAAATACAATTATATAAGGGCAGAACAATTTTACTGTTGGAGT
TATCACCAAGCTAAGGTTTTTTTTTCCTTCTTCTTACAAACCAAAAAAAAAAAAGAGAATAACCTA
GTTAGTTTGCTTCTTCACTCATTATTCTTTAGCAAGCTTTTCTCCTTCGCAAGTTGGGCATTTTCTCG
TTCGATTATTCGATATCCTTCTATTTATACCATCAGGGCTCGTTTCGTTTCGAATCGACGAAAAAAA
GAGAGAGAG

>*CqYAB2*

ATCAAATAAATACCCACGACTCATTCTACGGCAATCACAACTCTCATACTTCACTCTCAATACTCT
CATTCACTCTCATTTTATCTCTCTAAAAAAAAAAAAAGTAAAATAAGGAAATATTTGACTACCTCC
ATCTCATGTTTTTTTGCAACTTAGGGTTATTATTTGTGAGACATAAAATATTCCAAAGTTGTAAAAA
ATATGAAACAGAGGTAGTAGTTAAAATTATATGAAAATATCTTCCTAGTTTTTTTTAATAGGAAAC
TCTTCCTAGGTTGAACCTATGTTAATAATTGTGTTCTTTTATACTCTACACCATAAACAACAAAATA
AACTTATATTGATATTCCTTTTATTTGTTTTAGTCATAAATTTAATTTTCAGTCAATATGACGATTAA
ACTATTTTGCACTCACTTCGTCCAAATTTTGTAGAAATATCAAAATCATCATATTATTTTTTGCTCC
AATGTTATTCCATTCAAGGGGGAAAAAAAGCAAGCATATATAATACGGAGTATAACATTTGCTAT
CCCTAAAGATGAACAAAAACAATAAGATTGAAGCATTATATGTAAAAACGATGATGAATAAATT
GGGAGCTCGTTGTAATTGATTAGTGAAGGAAGACATTAGAAGGTGAGATACACATCATATAAAA
AAGAAATTTACTCGTATAACACAAGAAAAAACTCAATTTGTTGTTGTAAAAAAAAAAAAAAAA
AAAAAAAAGGTTAAGAAGGAAAGGTTGAAAAGAACAGATGAATGAAGCAAAGTTAATAAGAA
CATGCACACGTCCCTATCCGGTTCTCCCCCCCACCTCTGCCGCCTCTGCCCTTCAATTCTCTTTCT
CTCTCATCATCGGGAATGCCATCCATTCATTCACATTTTCATTTTCTCTCTCTTCTTTCAACTACAA
CAATAACATTCTTCCTTTTTTTATTTTATTTTCTCTCTCTAAATATATAATAAAACCCTAAATATATGT
AACAAATAAATACAACCTAGCTAGCCTAATTAATTACTACTGTAGTAGCTATGTTTAGAGGTCTGT
CAATCTATTTCTGTAAATCTATGCTTTCTTCCACCCAGCTCAACCCTACTTACCCTCTAAGCTTTCT
TATAGATCCACTTGTCACTTCTCTTTTCATGCCTTCAATTTTCATTTCTTCATTACTCCCAATTTAAA
TGGAGTACATCATATATAATATTATATGACGATTTTCAACTTTAATAATAATCTTCATTTTACAATCG
GTTGTATCTTACATTGCATTCTCCAAACACGCAAAATCGTATATATGGAGTAAATAACTTTGGTTG
TACTGTACATTACAAGTATAATATTCATAATCAATTAAATTAATTTGTTGGTTATAACGTTGTATTTT
TATCGTTCTTTAGTTTAAGACTCCGATAAAGCTGTTATGAATTACATATCTTGTTCAAATTACACTA
TCAAGTATCAACGAATGTATGTATAGTATGAGCTGAAGTTACTCATCTATAAGAAATTATTACAAG
TGAGTACCACACTTTTATCCTTGTTGATTTAGCACAATATACAATTAAAAAATAAATGAGTAATTC
TTGGCGAATTTAAAATTATGTTTTAAATGCATATCATCCTTAATATTTGAAGACAATTATTCAAGTT
TGACTTGGCCATGTATGGTCACACTTTTGGAGCATAAATTTTTGGGTGAATTTCAAGAAAATATCA
AACATTCATTTAGCTAGCATGCAAAATGCAATGACATTCTAGATTTGTGGTAGGATGAGTTTTAAG
TACATATTTGATTCTATTCTATTCATTGGGGATGAGCTAAATAGCACTGTATTGTGGAGTGTGGAA
AACGTAACAAAGGGACAAAAAGTGATTTTGGTGTGTAAATTTGGGTCCCAAATTATACATGGTCC
TATAATAAGTTGGGTTATTATTAGGTCATCATTGATCAGAGGCTCAGGATATATATTTGAGTCTCAA
GTGTATCTAGCTTGAAGAAAATTGTATGATCGAATGGTTTCTATTTTACAATCATGAATTAAAAGA
TGATTTTTAAGAATTCATCACAAGACATAGTACTATAAATAGGGTCTCACCATTTGAGTTTCGAGT
CCCACATTCGAGTTTTCAAAACTAAAATTGATGTTTTTGGGACCAAAAAAGAGTCCCCCCAAAG
TAGAATCCCTAAATTAGGAGGATGTTATTGTAGATACATGAACACTTCACATTAATTTGCATTGCC
TATATTTTTAAGGTTGATAAAGAAGTGGATGCCCCTACTAAGATAAGAACCATATCCATGACTAAC
CTATAGCAAGGGACCCTAACACATTAAAGGAGAGGGAATTTATCTTTTTTTTTTTTTTTTTTTTGAA
ACAGAATTTATCTAATTATTATTATATTATAAAATTAGGTTTTACATAGTACCTATGTAGTAATTAAA
GACTTTACCTCAACAAAAAAAAATAAAAAATAAAATAAAGACTATTCTTATTATAGCTTGTCTTTAT
CATTAAACTTGTTAATTAAGCATATATAGTGACAAAAGGAAAGGAACAAAAAAATGTAGTCTCA
CAGAATAAGTGTGAAAATTAATAAGAAGAAAAAAAAAAAGTGTAGCAAAAAAGCAGTACGTTTT
TATAAATAGAGCTTTCAAGAAGTGTTTATATATAATTGGTATCATCATAACACAATTTACACACATT
CACACAACAAATATTAATTCTTCACCCTATTCTCTGTCTTTCTCTCAACCCTCATTTCAAATCCCTA
AAAAGGAAGGCGGTTAAAAATATTAAATAAACTATATAATAGTATAATTTTAATTTAATATATTAA
AAAAAAAAGGGGGAGAGTGGCATGGGCTACTACAAATAAAAACATGAGTCAGTAAATCAATCC

ATCCATCCATTCATATATCCCATCAAAAAGATTATATCATCTCAACAACAAAAAAAATACACTTTG
AGTGAGAGTGAGAAATCTATAACACCAATA

>*CqYAB3*
ACCTTACAAGTTTTAAACATGCTTGCAAAGTTATTTATAAATATATGTTGTCGATTATTGGTTGCTT
AGTAGGTAAACAACTATTTTCTTTTAATAATTAGGTTTTTAACTTATTATTTTAAACATATTCAATCA
AATTTGCATATGATATGACATCTTTATATACATGTTGGTAGAATTTTAATTACAATTTTACGATTCGA
TTCTAAAAGTCCCTTCCGATTCAAAGTGGAATCTTGATTTTGGTAACTTTGAATACAATATATATAT
TAAATCATTATGTGATGGTTTTACATAGATTCAAAGGTGGCATCTAAGTCATTCAATTTGGATTATA
CTTGACTGATCAATTATTTAACTCACTTTAATTTATATCATATTTGACTCTTTAATCAGTATCGTATTA
TGATTAATCAATCAAAATCAGACAAAAATAACATTTTGTTTAGTTAAGTCAACCGCACCCAAATT
AAAATCAAAATTTGACATGTAATTATAAGCATAAATAGAATCTAGCTTAATCAATAGTTTGAAATG
GAATAATTAAGTACGAATTACTTTTGATAGGTACGTAAAACTTTCCATTAATCTAATTTGTTAATAA
AATTATAGATTGATATGAAACATAACTGAATTTTCTAAGTGAAAAGGGGATCCCCACTTCTTGAC
ATGACTAAACATAAACTTTCCAAATAACATACGATCAAATCACAGTAAATTTGAGCTCAATGTTA
TGCTAAAAATAGTATTCTTAGTTGTAAGATGGATTGCCTTTTACAATAATGCTACGTAAAATTTATT
TCATTGGCTACTTATCACAACCATAATATATACGGAGTACTAATGTTTGTGAACAGTCACCATCAC
AATGGTAAAACCAAAGGTTTTTTTTTTTTTATAATTTATTTATTTTAATTTTAATTATTATTATTATTAT
TAATATTCTAATTTTCTAATATTTTTATTATTATCTAGGAAGTTTATGTTTTTTCAAAGGTGACTTGTT
GAAATTATATTTTTTTTATTCAAATTTAAATGTAGAACTATCTATCTAACAATGAATAATATTTTTGT
TGAGATTAAAACACACTAACAATTAAGAAGATTTAGTTCATATATATGAATAATTTGTTGTCGATT
AAAGCCATAAGCAAATTTTAGGATATGATCTAGTTCTTATTTTAAATAATTTAAATAATAATGCTTC
ATAATGTAGGAAAATAACCACACGTACAATTAGTATTCCACTTCATTTCATAAACAATATACTCCG
TATTAGAATTGAATTAGGTATCACTATTTTTGAATTACTTTATTATTAATCCATAAAATCATCAACAC
ATGAAAAAATATTGTACTTTACCGAGATGTTTGTTTTCCACCTTAAAATCGGCCACAAACTTGAA
CAAGCTTAAAGAGAAGAGACAATTCTTGATTGTAATTAAACTTTAAGTCTACAATCATTATTAGA
CATAAATCATTACTCATTACACTGTTTAGTCCCCCTAATGATTTCTTAAAACAAACGAATCGATAA
CAATAATGATGTGAATTGAAAGAAATGATGAATTGGCTGTTTCATTATGACACCTGCTCTAAAAA
GATTTTGGACTTTTCTCCCATTCATATGGAACTTAATAAAGGATCCAACTTCATGCTTTAGGTTAA
AGGGACACATCTATTATTTATTTTGGTTATCTCGCGTTGATTTATTGGTTGCAATTTAACATTTGTAA
TAAGTGTTAAGTGTTAATCAATACAATACATGTTTTTTTCACCAAATTGTCCTATAAAGTATTAAGG
TGACGGGAGATACGAGAATCCTATCACATACGGAAGCAATTGGGGAAAACAAAATAATCGAAA
TTCCAAACAATCAACCTCATAATAGAGAGGAGCATACAACCTTTATCATTGAGCTCTTACTTGATT
GTCTACATGCACATGTCTATGAGTGGCGTATACGTAGTCATTATAATATACATTCATCTTTTCTTGCT
TCACGGTTATGTAATTAGCGCTCACACACATACAAATCCCTTACATTACACAATGGTAGAATTTTA
TGAGAGATACATCTCATAAGTAACAACCATCCAAGCAAATGACTAGAGCTAAAGCTAGTTTATAC
TACACTTGTATGTAACATATCATTTTCGTTAAATCCATAAAGAAAAGGAAGGAAAAAAAGGGGA
AAAACATCCACAAAAATTCATAAACCTCATGATCTCATCCACCTATTGTATAGTGGTAAGGAAAG
GGGTGAGAGGGACTACACATACACACAACCTTGTTTCATAATAATAGCCAGCAATACTAACCTGT
CCAATTTTATTAGTCCTGCCAAAATCATTCATATTTATTATTTACCCGTTTTCTCTAACTCTTCAATT
CACGTTATTTTTTTTCTTCTCTAAACCGTCCGGAACCAGCCTAAATATCCAAATCTCGAAAGAATT
CCCTTTCTCAACCCCACCATTTTCTTGTTTGTAGCCTTCAACACCACTTCTTTCTTTTTTTTTAATAA
CTTTAATTTTCATTAAAAAAAAATCACCCAACTTTTTTTTTTTTTTTTTATAATTTATTTTCTTTCCA
TCTTTATAGAAATAAATAATTGAAGTTCATTTAACATTATTAGCCCTTCCCTTCCCTAGCTAGTCTTT

TCCTATCAATATTTCTCTCTCTTCCTCATCTTCCGGGCTAAATTTCTCCTTTTATTCAGAGAATTTCT
GTTATAAATACAATAATAAGGGCAGAAGAATTTTACTGTTGAAGTTATCACAAAGCTAAGTTTTTT
CTTCTTCTTCTTACAAACCAAAAAAAAAAAGAGAATAACCTAGTTAGCTTGCTTCCTCACTCATTC
TTTAGCGAGTTTTTCTCCTTTGCAAGTTGGGCATTTTTTCATTCGATTATTCGATATCCTTCTATCTAT
ACCATCAGGGTTCGTTTCGTTTCGAATCGACAGAAAAAAAAAAAAAAAAAAAAAAAAGAGAGAG
AGAGAG

>*CqYAB4*
ACAGAACATGCAATGGATACTAAATATACAATCACCTTTTGCAAAGAGATGGAGAATGACGACC
TACAATAGTCCACGACAACCTAACATCTAATTGAATATACGAACCTTATTATTATTCTCTTCTTTTC
GTTGTCTTTCGCCACATTTCCAGCCAACTACAGAATTTGTTCATTCACCAATACTCAAGATGGCGT
TACTGATTTAGGGCTTAATTACGTGGATTTAAAAAGGCCCAAGCTCTTCAAATACCTCATAGGGA
AAAATGATATGCAAGTATTGTTTGTTAATAACAACCTAAACAAGTTATCGACATTATATAATCATT
GTTCAATTGCATATGCTTCAATGCTCCATAAAAAATGCTAAAAGATCTTAGTAGTTCTCCTAACCC
AAAGACCCTACACTCCGAAGATGAGACCCTATTCATTGATAAAAAGACCCTACTCTTAGTCAAG
GTAACCGTACTCTCATGAAGGCCCTGTTTGGTTTGATGTAAAAGGTTTTCATGGAAAATGAATTTT
CCATTTTTCCCCATTTTCCTTTGTTTGTTTTGTTAAGGGGTGGAAAACAATTTTTCCAAGGGTGGA
AAATTCCATCCTTAATGATGGAAAACATTTTCCTTCCCAAAACAAGGGAAGCTACATTTTCCTTTC
TCCTCCTTACCTCTTTTTTTACTTCCCTCTCATTCATCTTTACATTTCCACTCTTATTTTTTATTTTTCT
TGTATGCAACCAAACAAAGGAAAACTAATTTGCAATTGTGTTTTTCCTTGAAAATATGTTTTCCAT
GAAAACCAATTTTCAATTGAAAATGTTTTACATTAAACCAAACGGGGCCGAAATGAGACTCAGA
TTTTCAAGAATATGGGGATGATTTGGTATAGCTTGTTTATGGTTGGAAAAATAGGTATTTTGTTCGT
CATAGCCGTTGCATTAGCCACTTGCTCTTGAGGATTCCGAACTGAAAATCTCTTGATGAGAAACT
GTAGGCCAAATGTATTTCCAATAATGTGTGGTTGTTGCACCCGGTTGCACTTCCAACCAATGTTGG
TCTATTAAAATTCACAACTAGACCAATTCTCTTCCTTTTGTTGTCGATCTTTTGCCGCGTTTCCAAC
CAACTACAGAGCTTGTTCATTCACCAATACTCAAAATGGCCTAACTGGTTTAAGGCTTATTTACGT
GGATTTAAAAATCTCTAAGTTTTTCAAATACCTCATAAGGCAAAATAATGAGAAAAGAACAAATT
ATGTGTCCACAAAATACTAGTCCAAAAAATTGTCTCACGTGAGATGCACGTGCAATGATAGACTT
GTTTTTTCCTAGCAAAAGTTGTAGGTGGTTGGGAATTAAATTTGGGGTAAATTTCAAAAATAGATA
ATAGAAAGAAACACAAAGAACAAGACATAAAACAAGGTAAAAATAGAAGGTTAAAAATCAGC
ATGTTTTAAGTTTTCAACTCATTCATAGACTATGGTCAATTATGCAGGCATAACCGTATAAGTATAA
TATTTCTTTGTTCCATGTAAAGACAAATCCTAAGATACCTCCTTTCTATTTTTGTACACATTTCTAG
AAACTTCATCCCCCACTAAAAAAGGTAAAAAATCATCTCACTAAACCCTAATTTAGAAATAATG
ACACAAAGATATAGGAGGCAGAAAACAACACTTAACAAAATAAGACATTGGTCCCATTTTTCCT
TTAATTTCCTACCATCAAACCAACAACATTTTGGAAATCTCTCTTTTTTATGTGATGCCTACCAAGT
GTGTGTCAATGTTTGGAAGTAAGTGTGTGACTTGAATTCAATGCATTTATCAGAACTCTAAGTGA
GGTCAAAATTCCCATTTCGAGAGTACGTGTACTTGCTTTTCAATCTAAACAAGACTTCCAAAAAT
TTATGTGTTAGTTGAGTAAGAAATCTATGAATATAATGAAGTTCTCCAATAATTTGGTGTAATGGTT
AGGAATGCAAGGCCGATCATTAATGTTACCTATGATGATTATTTATAATAATTCTAGAAAAATTATA
TATTTTGATGAGTAACAATATAATCTTACGCACGGATCTAGATTTCTTGAAATAGATCCTCCCCTCC
TCCTTTTTCATAAGTCATTCAAGTATACTGTTATCCCCTCTTTTTAGAGGGTTTGTACCTAGGTTTAA
TGTTCAAATGTTCAATGACATATATAATAACTAATTTGCTAGTATAGCTTGGTATTTTACATGTCAA
AGACTAATCGACTAGAGTCATTTCTTCATTAATAGGATAAGTTTTTGCTTGTTGAATGTGGAACAT
TGATCAACCACTATCACTGCGAGTCTATTTTGGACATTCAGATCAGGGACATACAATCATATTAAA

TAAGGCACATTTATGTATCAATATATGTACCAATTAAATTTACGATGCTAGTGATTAAACTAGCTAG
AGTATAAAAGGCCTCTCAAATAATCGATTGAGATTTTTGTGGTTATTTGTGGTTCAAAGAAGTGTA
TGTGTATGTACATGTCCAAATGATGAAAGGATGACAATTCGATTATCCTAATACAAAGTATAACC
ATCAATTAATAATCCGCTCCTCTTATTAATTACATCAACTAACCAAAAGGAAAGTTAGATTGCCTA
TAGATACAATCTAACAAAAATTTGATAAAAGCTACCATATACATATAAGAATTATCACTAACAATT
CACTGTCCTCCCTAGCTAAATTCTAAAAACACAAATTTTGGTCATATAATAAATATTATGAAGGAT
TCATTTATATCCTAGGAATAAATTAAATTAATATGCATTATTAATTAGCTACATATATACTGTTATGTT
ATAAGTCTTATAACACATGACTCACTTAAATGAAGTCTGTTGTTTTTTCTATATATATTATGCATATA
CTTGCTCCTACTCTTAGAACCCTAAAAACCTACAATTGCACAATTACATCCAACCCCATTTTCTCT
CTCTAAATTCTTGGCCTTCATCAAGAATA

>*CqYAB5*
TGTTTCAATTCATCTAATTGATCCGAGTTAGCTATCCAGTTCCATGTTACGGTTTTATTAAAGATTAT
TTAGTGAACGTTTTTTTTTTCTTTAATACTCCATATAATCATAAAAATTTAGTAAATTACTATTATTG
AAATATTATTGGATTATTATTCATCATCAAGTCATTGTTATCCAAGTAAATTACTATATAATTCAATT
TGGTTGATTGTGTTGGATCAAATAATTGATGATGAAATTAGATAAAAAAAATTAAAAAAAAAAA
AGAAAAGAAAAAGAACATTATAAAACCATCATATTGTAGAATGTTCATATATTGATATATTGTGCT
TTTGAAAATCAAATAAGAATGATAATTAATATGCTATTATTTAAAAAAAAATTATCATTGTTTTGAC
CTACTAAAAAGTACATTAGAGATTTAAAATGATTCTACTAAATTAACAACTACTATGGACAACAA
TCATTGAAATTCGAGTAACTTTTATTGTAAAATACGTTATGTACCGTTTCTTCAAAAATTTAATTAC
ACAATTATAGCTTTAAATCCTTAACATTAATTATAAGCATTTGGAATTGACAATAGTTGGCCGGTA
GCTCTTTTTTTTTCATGAATGAATACATAAAATCACTTATCAACTTATCGCTAATTTTGCCAAACAT
GCACGCCAAGTATTTATTTATTTTTCCTTATATACTCCAATATAGTTCCAATGTGTTATCTCTCAAGT
CTCAACTACATACAAGTAGAATTACCTAACAAACCTCTCATATTAATGGTAAGATTTTATACAAAC
AAGGAAAGTTTGGGAAATCAAAGTGCAAAAGTAGGCTAAATCTCCAATTTGGTAATCTAGGAGT
TGAATCGTCTCATTTCAAACAGCTCTTCCCAAATTCATTAGTGATGGTGACTAGTGATTTCAGCAC
TTGTCTAAATCAATGCTTTTGTTTTTTCTTAACATGGTACTACTCAGTACTCACTAACAACATCAAT
TAGATTTTCTGTCTTTTCCTCACCCTCTTTCTTACTTTGCCCTATTCTAATTAAACACTAAAAAAGT
GAAGTGAATTTCTAATCTAAGAAATTCAAATTTTATAGATACATAATTTAGGAAATTGATCACACT
TTTGTTACAAAGTTGCAAAGGAGTTGCTCTTAGTTTGGACTAGTCTTTTGATGCCACCTTCACGTG
TACGTGAACGAATTGATATTGCTACTCCATAACTTCCTCAATATTTATACACATCCCCGCATCTTGG
TCAATTTTATTTTGACTATGATTTTGAAGTGGCAAAAATTTTTATTTAAAGCTAGTCTGTAATGACA
ATAGTCTTATCACATAAATCAATTAAGTTTGGCTAAAGTCTTAAACGTGTTATCCAAAACTTGAAA
TCATATATATCTTGTCTATATCACTGTTATTAGTAACTCTCTCCACACACACAAAAAAAAAAAAAA
AAACAACCAATAAATTCAATATGTAAAAATCATAGTACCGTTATCTATTGTGTCTACTATGTGATTT
AGAGTAGTTTTATATTATTGTAATTCTTTCATTGCCTTAATTAATAGAACATTAAACATTTCTTTGAG
CTACAACTACAAAAAAAATTGTACTCCAGTTGTTTATGAGAATGAATTATGATTACTTTGTAAACC
CTTGGGCAAGTATTTTGAATAGCATAAGGTGAACATAAATAATCACCACCCTACTCAAGTCAAAC
ATATGAGATTATGAGGGCAATTTTTAAATTCTAACTCATACAAATCCTAGAGGATCATTATTATTAG
GGTTTATATATGTATTGAAGGATTTGAAGCGGAGAAAGTATATAATAATTTTATTTATTTATTTTGAC
ATGTAATATGAATTTTACTTATCAACAACCATTCTCAAAAGGGAGTCCTTTGTACATGGAAATTTT
GAGAATTTATCACTTTCCTGTTACAAACAATGATGTTCAACTACCCTACTCCAAGTAAAAGTATGT
ACATTCAAATCATAAATAACCTTTGATAATTACTTAATTATAATGATAATGTGCCCTCCTTCTTAAA
ACTAACATTAGGTCATGAATTCATGACCTGCTTTCACCATACCATGCAATACAACGTGCATACTAT

GTAGAAAAATGGGTAATTACAAGCCTTTAACAATAGGTGATTGTGATCTAATTAACCAACTTAGT
CACTTAGTCGACGAGTATGTGTCGAATGTCGTATAATCGGTTGCACCATGCACAGGCACACCATT
ACTCATTTTTTGCTCATGCGGAGCTATAAGAAAGATATAGGCAGGCATGCATGGTTATATAACTTG
TATGATACAATTGGTTCAAATATAAATTCATACTGTCTTTTAAGTTGGTTTGTATTCTAATTTATAAA
CAATTCATACATGTATGTTCTTATACACACACAACTTGTATCATTTAATTAAGCACACATGCCAATA
CATATCTTTAACCATTTATCACTAATTTTGCATTCGTATGTAAGAAATATATAATTATATATAAATAA
AGTACACATTAAGACAAATTTAACAAGATCACAAATGACAATAATTTACCTTACATATAAATCAC
AAAAGACCATTAGAATAACTTAATGCATAAATTGTGAAAAAAACAAACAAACGAATCATCTAAT
AAATGATCGAGCTAGCTTATCATAATACAAAGCCTTGCATTAGTTGCATAGTTGTACGTTAAAAA
ACTCACAATTTGCAAAAATGAGCACAAAAAGAATAACCAAATTAGAAACCCTAAAAATCACTTT
TTACGATATCCAACACCATAGACATTTAGGCAACTATATATAGCTTTCCCCCTCTACTATATGTATA
CCTTAAAACCTAAACACATTTCCTTTTATCTCTACCCTATCATCTTTTTAACTATTTCTTCCCTTTCA
TTCTCAATCACCAACATTATCTATCTCTTATCCTCTCTTGTTTAATTAGCTAGCTTCGATCATTCTTC
TTCTACGATACG

>*CqYAB6*
AAAGAGATAGAGTTTATATATGTTACATGAAATAAATGTTTTTCTCTCAGTTTAGATACTAGTTTCT
TTACTTTAATTACTGTATTGTACAATGTACATTATTATATAATGAAGGTTGTTTCATCATCTATTACTA
TTATATATATTACCATAATATCAAAGTCTCTCATAATTAAGTTTTATGTAGGAATATATATTTACATA
GTCTAATATTGTATACTTAGATTGAAATTTTATATTGATTGATACTATGTATATAGTGACATTATATAG
CTATATAGGGAGATGTATGTCATGTGGATAATTGCTATAGGTTGATAGATTTTATACTTGAATATTAT
AGATTTAAAGAGCTTAATTTATGATAAGTTTAGGATCAAAGGGTTAATAGCATTACTTGTCATTTTC
ATCAATAGGTAGAGTATTTATACAAAATTATAAGATAACATAAAATAGAACACCGTCTTCCTAGA
GCTATCCCTCACTCTCTAGTATCTCTAACAACTTTATATAATTAATTTTAATAAGAATGATTGAACC
ACCGCACATAGTTTATTTGTCATATGTCCTAACAGACTTTATATTTTTATACTAATAAAGACTCTGC
AACCCAATATATATGCAAATAATCAATTAATACTCCGTACAAACTTTATAATTAGTAAACAAGCAT
TATACATGAGTGCTTCGTTAATTTATGCACATACAGAAACTATACAAAGGTTATGAATGGCCTCAA
CACACCAAACAAAATAAACATATACAAATTCAATTAACTTATGACCAACTATTTCTAGTAATAATT
GAATAAATATCTTCATATATCAAAGTAATTTCCAACCTCTAAAATTCATGGTAATAATTGAATAAAT
ATCTTGAACAAAAATTAAAGAAAATTGCTAGATTATTACAAATTAAATTTGCCATTGATAAGTAC
ATATATATAATGGGCTAGTGGCATAGGGCCATTTTTTCCTTTACTTGTTGACTATGACAATTACAAT
AAAGGTGGAGATAGTAAACCTGAAAATTTTCACTAAAACCAAATTAAAAGACCCAATTTATGTT
GGCGTCACACTAGCATTAGTTGCCAATTGTCAATAAGCCCTTGATGATCATATCCTCTAGTTAAGG
CCTAAGGGTCCATTTTATTTGGCCATCATACCAAATTAAAATGACCCCATTTTTTCAGCTAGTCTTA
ACACGCTACCTTCACTTTACTAATTCCTTCTGTAGTTTCATACCTTTTGCTTATTATTATTCCACCCC
CACCAACTTCTATTTCTCATTGTACAAATATTCAAGGGCGAGATAGTTATTGATAAATCGCGTAAT
CTAATACAGAGATGTCTTAGACGACACAGACTTACAATAATTTATATATTGTATTGAAACATTAAC
GTTAGAAATTATCATAACAATATATCAGAGTGAATTCTACTTGTTAACCCAAGTTTCATTTATCTAT
TCTTATTAAAACTTAACTTGTACATTGTAAATTGAACTTGATCAGGTAACTAGCTTCTTATTAATAA
CCCAGGTTTTAGCTTATATTTCTTGAACTTGTCTGATCTTGACTTATTAACCCTACTCTATTAATTTT
AATAAAATAAGTAAAAATAAGGTGAACAAATTAAAACAAATGTTCAACAAAGACTAGAGTCAT
CAACCCTTAATATTAAAAGTTACTTTATTAATTATAACATACTCCCTTTGTCCCTATTTGTTTGCCTC
ACTTTCCAAAAAAGGGTGTCCCCTTAAATATTGCCCCGTACCATATATGTTACTGGAACCACAA
TTTTACCCTTACAAAATCATTTTAGGTTCCACCATTTTTCTCTCTCATCAATTCCACTCAACCAAAA

ATTAAATAAGCAAAATATGAGCATGGGACAAACAATTAGGGATAGAGGGAGTACTTAATTATTTG
TGCTAGAATGGTGGAGCTCTAATTGGTGCGGTAAGTAGGCTTCAAGTGAGCTTCACTAATTTGAG
CAATGTAGGGGATGAAGACGATGTTACTCCGTACATTGTAATGGACCTTCAAAGTTAATTTTCATA
TGCATCAAAGAGCTAGATGTAAACAATTTTAAGTAAACAAACTAAACTAGTTAAGTGAAAATAT
AAGGAACTATATAATGAAAAAATGTAAACTTAAAACCTAAATATGGTATAAATTGCGGCAATAAC
TTTTGTTTCCCATGTTAAATACAGAGATTCAGAGAGCGATGCAACATTGACTATAAGAGAGAACA
TTTTGAAGATATAACCTATTGTAAGTTTGTAACATAGAGTAGTAATCATCGTTAGAACATGGACAA
TATTCACAATAGTTAAGAAAAGAGATAAAAATGCTCCTTTTAACTACAAAACATACGTGTTTTTG
TCAACAAATGTGATTTTATTAAATAATCAATTTTTAGTTTAACCATCTATATTAATGTGAATAGTCTG
GAGTAATAATATTTTAAGTTGTGATAGCTTAATAATAGTAACCCTAATGAGATGGTATACTCTACTA
ATTTTCTTTTGCTTCAGTATATATCCATCTTCACATTAGAACACCTAATTAATCTCGCGAAAATAAG
ATCGCTCCTACGTCATTGTCCCCGTATGCATCCAACTGTTGTTTTAAGAACAGGAGAGGACCCGA
CCCGGCCCTCGAAAAACAAGGGCTTGACTAGGTTCCTATATGGCTAAACATCAAATCCCTTATCA
CAAACATAAGTTGATCGTACGACTCCATCCCACTTTGTAAGATCCTTTCCCAACCGACCAGAGTC
TTATGTATGGACTATAGTCTGTTACAGCTTATTTTACTTCTTTTCTCTTTTTTTCATTACATTATCTTTC
TCTCTCCTCCTCTTTTTTTTCCCTTTCTTTCTCTATTTTAGATTGATCAAATAAAATTGAAGTAATAG
CACAAAATAGTGAATTTTCTTGTCTATTATATACTTGAAATTTTAAAAAAAAATCAAAAAAAAAA
TCAGAGATAATCG

>*CqYAB7*

TATTTTACAACCGGTTGTATCTTGCAGTATACGGAGTACATACATGTAAAGGATCAAATAACTAAC
TTCGGTTTATACATTATAACTTTGGTTGTACACTATAATATAATATTCACTAGATTAGGTCACATGTC
ATACACGGTAATTTTCACTATTTATTATTAGTTTAACAATATATTTAATAATAAAAATTATCTAATTG
CATGCGAAATAAAATGAAAAATTCGTAATTACAAAAATAAAAATGAAACTATATCATACTATAGA
AAATCGTGGGAGTATTACATGCAGATTACGTCACAAAGTTATATGTAAGAAAATATTATTGCATAG
TATTAAATAAAGAATAATATTTTTGTTGAATGTGTATAAATGGAATATAATTACCAAATAGTAAAG
GAAATATAATTTCATAGTATTAAATATTCTCTAATAAGCTTTGGAAAAAAAAAAAATTATCTTGGCG
GAAATTTTTTTTAAAAATAAAGAAGATAACACTAAATTTTTGTATTAAAGTCAATATGATGCATTA
TAACGTAAAAATGTTTTTATTATTTCATACTATAAATTAAATCCATTTGAGTGTAATTGATCAAAAA
AAAAAATCAATTTAAGCAAATAATATTAATATTAGTAATAAGAATCTTTAATTGGTATTATAATATA
GAAATTTATTGTTTTAATAAGTTGTCAGTTAAAAAGATATAAAAAGAAATATAAAGTTAGAATAA
CATTTATTTTTGGAAAATGGTTTTTAGAGGGAAAAAACGACATCAGAAAGTGACATTTGTCATTC
CCGGTTCTGTTTTAGTATTATGTTATTGATAGGGTTCAATCAATTATACCTAAAATATTACCTTCAAA
ATCAATTAAATTAGTTGCTACTCCGTATAATGTAATACTCATTCCTTAGCTTAATGCTCCGTAGTTAT
TAAGTACCTAAAAAGTTGTTAAGGATTACATAATTTATTCAAATTACTCTATCAACGAAGTTATGT
ATAGTACTCCTTTTTTTTATTTTTTTTTATTTTATTTAAAGACTAGAGTTGGAAAATTCCACTATATAT
AGGTCATTATTGACTTAATTTTTTTTACCAATATATAATATATAAATATGATCATATAAAATGTTGTT
GGATTTGTCATAATGTCTACTTTTATAAATTATTTTTTTCATAATTTTTACTAACCTCTAATTAAAAG
ATATTAATGGTCAAAATTGTGAAAACAAAGGGAGTATGAGGATACTCATCTATATATAAGAAATT
ATTACAAGTGCATATCACACTTTTATTCTTGTTGATTTAGCACAGTATAATCAAATGCATATTATGA
TATTATCCTTAAACTTATTATGTTTTAAATAAATATTGTCCTTAATATTTGAAGACAATTCAAGTTTG
ACTTGGGCATGGCCATTCTTTTGGAGCATAAATTTTTGGGTGAAATTCAAGAAAATATCAAACAT
TCATTTAGCTCGCATGCAATGACATTCTAGATTTGTGGTGGGATGAGTTTTAAGTACATATTTGATT
CTATTCATTGGGGATAAGCTAAATAACACTGTATTGTGGAGTGTGGAAAACGTAACAAAGGGAC

AAAAAGAGATGTTGGTGTGTAAACTTGGGTCTCAATTATACATGGTCCTATAAGTTGGGTTATTAT
TAGGTCATCATTCATCAGAGGCTCAGTATATATATTTAAGTCTCAAGTGTATCTAGCTTGAAGAAA
ATTGTATGATCGAGTGATTTCTATTTTACAATTGTGAATTAAAAGATGAATTTTAAGAATTCACAA
GACATATTACTATAGGGTCTCACCATTTGAGTTTAGAGTCCCACAAACAAAAATTGATGTTTTCGG
GACCAAAAAAGAGTCCCACCAAAGTAGAATCCCTAAATTAGGAGGATGTTATTGTAGATACATG
AACACTTCACATTTGCATTGCCTTTATTTTTAAGGTTGATAAAGAGGATGCCCCTACTAAGATAAG
AACCATATCCATGACTAACCTATAGCAAGGGACCATAACACATAAAACTATAAAAGGAGTTGGA
ATTTATCGAATTATTATATTACAAACTTAGGTTTTATAGAGTACGTAGTAATTAAAGACTATCCTTAT
TAAACTTACTCTCTCTATATGTTTCATATGTAGTACTGTAAATTTGCAACCAAATCTTTGACAGTAC
TCTTTGAGCAAGCGATTTGCTGAAAGAGCAAACGAACTAATTAGTGTAAAGACCTTAAACACTG
ATAAGAGCTGAGGATGTAGTATAGTAAGTTGAGCTAAGACCATTAAGTTCTCATCTCTATAAGAA
ATACGGAGTACTTCATTCAACACAAACTTATTACTTATAAAACTTGTTTTTAAATAAATTCCTTTAT
GCATGAGATGTAAATGACAATGTATATAAATTTTATTTTGTTAAGCATATATAGTGACAAAAGGAA
AAGAACAAAAAATGTACAGTCACAAAATTAGTGTGAAAAATAATAAGAAGAAAATAAAAGTGT
AGCAAAAAAGCACTTTTTATAAATAGAGCTTTCAACTTGTACAAAAACTAAGTGTTTATATATAAT
TGGTATCATCATAACACAATTTACACACACTCACACATTCACACAACAATTCTTCATCCTATTCTC
TGTCTTTCTCTCAACCCTCATTTCAAATCCCTAAAAAGGAAGGCGGTTAAATATCACTCACTACTA
TATACCCCTATATAATTAAAAATATTAGATAAACTATATAGTATAGTACATCAATATAAAAACTATA
TAGTATAATTTTAATTTTATTTACATTAAAAAAAAAGGTTGGACTGGCATGGGCTACTACAAATAA
AACATCACCGATCAGTCAGTCAGTCAGTAAATCAATCCATCCATCCATTCATATATCCCATCAAA
AAGATTATATCATCTCACCAAGAAAAATACTCACTTCCTTACAAAAACTTTGAGTGAGAAAATAA
ACTATAACACCAAGAAAAAAATA

>*CqYAB8*

GATGGTTTCTTCTCTACTTGCTTGATATTTTTCAAATGGTTATACAAAAAATTATACTATACAAAAT
TTAATGAAACCTAGCTATATATAGCATATAGGACCAATTCAAGATTATGAGAAGTTCCATTGTTAC
CACATCTGCTACCCTTGCTATTTAATTGGCTGTAGCTACTAATATCTATATCTGCCAAGTAAATAAT
ATTTGAGCATTTCTTCAACTGCTAGTTAATGGTTTTAGATGTTAGTCTTTCCTCTCGAATCTCCTTA
ACTCCCTTTGCTTGCTGAGCTTAGCTTGATGCATATGAATCAGCAATAAAATACACTATCTTGCAA
TTATATATGAATCCGAATGGCACCTTCATACATGCTTATAGATAAAATAGCAATCTTAGAGTGTGA
TACGAACACTTATTTAAGAATTTAAGATGATAAAATGTGACATATATGCATATAATCATAAGACTT
TAGAGGTACAGTTTCACGCTAGTTAACTTAATCATAGTCAGACACTTCATAACTACTTCATACGTA
GTATTTAAGTTACATATACACTTTCAAAGAAGCATGAGCAATAAACTTTTAATTCACTAATGATAG
GGTGATTTGATTTCTACATAAAGTCACACATGGTACCACAATAGGATCAACCAAAAAAAAAAAAA
AAAAAGAAAAAAGAAGAGGCCTCTCCCTCGATATTTATCAACTTAGGTGTTAATATTTCTTGACT
AATGTAACTATCTACAATCGCACATACCAATGAAATGCAAAGAAAGTGGTAAGACTAATTAATTT
AAGTAAGAAATGCTTTAATTGTTTGAAGTTTGGAGCTATAGTGTTACTACAGTACTTTACAAGTCG
TCAGGCAAAGAATAGTAATAGTGTAAACTTGCCCATATTATAGAAATTACAGCGGGTTGACCTAT
TGAAAAAAAGAGCCATACCATACATTGTTTTATCATCCCTAAGATTAATAAATTAATAAATAAAG
GACAAAAAAAAGGGGGGGGGGGAGAGGAAAATAAGAACCACATAGAATTGAAGAAGCAAA
TTAAAGAAAGGTGGGGTGGTAGCCCCTAATTAAAAAGTATGAAAGCAACAAGTGAATGATCAA
AACAATATGCAAATGTCACTTTCTTAGTCTTCTCCCATATCTGGCGCTTCTGCTTCATCTTCATTTT
CTTCATCTCTCAATTCACACAAATACATGCTATTATCTACACTATACTTGGATACTTTGGGAATACC
GCTAACCTTCACATACTTCTATGGATTGAATTTCTTACTTGTTATAATCCCTATATATGTATTCACCT

ACTCTCTATCCCATCAATCTTCTGTTGTTTAGGGATCCCATGTAATCATGTGTCTTCTCTTGCAATCT
TCACTTTTTCTTAGTGCGTAAGTTTTTGTAATGTGTTGTTTCCTACCATTTCCCAATTTGTTAATGGT
GCATTCACTTTAATTGACGTCTTATGAACTTATCTGAAGTTATTTGATATGGTTGAAACTTATTGAA
CCTAGCTGATTGGATCTTATTATTAGATCGACCTAATTGCAAGGGAGCAAACTTATTAGGCGTAAT
TGAACCTTACAACTTATTAGAATTGAATGAACCCATTTGCACTTATATATTGGACTTGTTACTTATT
TTTTAAGGTTAAGAGAATGAAGCCTTAAGATATATACACAATTTAATTTAGCAAATAATCAACATA
AAACTATTTGGAATGTTGAAAAAGGTTATTATATACAGAGTATTCTGAATAGCATGCACTAGATAT
GATTGGATTCCAAGATATTATGTAAGACAATGATTATGTTTAAAAATCTATATTGTTAATTAATGAG
TTAGGACAATGTATACAGTGGCATTAAGGATTCACTAGAAAATTTGGCATACAAAGGAAAGTAG
AAGATCGTCATTACTCTTGTACATAAATCCATAATAAGTGTCTTCTCTGACTATACACAACTAGCA
CATCAGTTATAGACACATCTCTAACACTAATATAAATTATGAGTAAAAGGTACAGTTAAAACAAT
TGTTACAAATCATTAAATTTGGAATCAATCGGATAGTAGTCCAAAAAGGTAGAGGTCATTTATGG
TGAGACTGAATGAAGTATCTCACACACATAATGTTAGACAAACTAAAGTAGTATACATCATATAC
ATAACTACCATTGGAAAAATGAGTGAAGTAGTATACACCACATACATAACGTTACATTGAATGAA
GAGTACATCACATACACAATCTCAGACAGATTGAGGTGCATCACATACATAGCTGTTAAGCAGA
ATGAAGTGTACATCACATACGAAACATAGCTTTCGGCTGAATGAAGTGTTCGTCACATACACAGT
ACAACATTAGATAGAATGAAGTGTTCATCACATACACAACATTAGACAGAATGAAGCTAGTGTA
CATCACATACACAATTTTATATAAATAATATAACATACATAATGTTAGGCATAATGAAGTGCACATT
ACATACATACATAACGTTTAACATAATGAATCAAAGTACATGACATGTATGCATAGTGGTAGACT
GACATGCATTAAGATGATATAGGTGTAGAGGCAAATGGGTTATAAAAGGAAACATAAAGGAACA
AATTAGAGAATAGGGATGAATGAAAATGAAGGAGAGATAACCAACTAAGGACACTCTTTATCTC
TCTCTCTCTCTCTCTCTCTCTCTCTTATAATTAACTAGTAGATCAAGAACAGCTCTTCAATTCCCAC
TATCTACTTCTCTCTCTTCCCACTCACCTGTTTTTCATTCTGAGCAGAAAATTAAAACAACACAAA
GCTGAAAAATAAGCTAAAAAAAAAAAAAAAGGCAAATTAAATAGAAAGGATCCAAGGTGTTGG
GGGTTTCATCTATCAAATCAAATCAAATCAAATCAAATCAAATCAAATCTGAGAAAATCAAAGG
ATTGATCAAATTTCAAATCAATTTAAAAAGAAAGAAAGAGATCCAAGGAA

>*CqYAB9*

ACAAGGAAAGTTTGGGAAATCAAAGTGCAAAAGTAGGCTAAATCTCCAATTTGGTAATCTAGGA
GTTGAATCGTCTCTTTTCACATAGCTCTCCCCAAATTCATTAGTGATGGTGACTAGTGATTTCAGC
ACTTGTCCAAATCAATGCTTTTGTTTTTTCTTAACATGGTACTAACACACCAATTAGATTTTCTGTC
TTTTCCTCCCCCTCTTTCGTACTTTGCCCTATTCTAATTAAGCACTAAAAAAAGTGAAGTAAATTTCT
AATCTAGAAATTCAAATTTTATAGATACATAATTTAGGACATTCATCACACTTTTGTTACAAAGTT
GCAAAGGAGTTGCTCTTAGTTTGGACTAGTCTTTTGATGCCACCTTCACATGTGAATTGATATTAC
TACTCCGTAACTTCCTCAATATATATACACATCCCTGTGTCTTGGTCAATTTTATTTTGACAATGATT
TTGAAGCGGTAAAGAATTTTATTTAAAGCTAATCGGTAATGACAATCTTATCACACGACTCAATTA
AGTTTGGTTAAAGTCTTAAATGTGTTATCAAAAAATTGAGATTATATATATCCCGTCTACATCACTG
TCATTAGTAACTCTCTCCACACAAAAAAATAACAAACCAATAAATTCAATGTGAAAATCATAGTG
TCGTTATTTAATTTATTGTGCCTACTATGACGATTTAGGTAGAGTAGTTTTATATTGTAATTCTTTCAT
TGCCTTAATTAATAGAACATTTCTTTGAGCTACATCTACCAAAAATTTGTAACTGTTTATGAGAATT
ATGATTACTTTGTAAACCCTTGGGCAAGTATTTTAAACAGCATAAGGTGAACATGTTTAATCACCC
TACTCAAGTCAAATATACGAGGGCATTATTAAATTCTAACTCATACAAATCCTAGAGAATCATTAT
TATTAGGGTTTATATATATTAGAACGGAGAAAGTATATAATTCGTTGTAATCTTCTTTATTTATTTTTG
ATATGCATATGAATTTTACTTATCAACAACCATTCTCAAAAGGGAGTCTTTGTACTTGAAATTTTTG

AAAATTTATCGCTTTCTTGGTACAAACAATGATGTTGAACCACCCTACTCCCAGTAAAAGTATGT
ACGTTCAAATCATAATAACTAACCTTTGATAATTACTAATGATAATGGGCGCGCCCTCCTTAAAAC
TAATATTAGGTCATGATCTGCTTTCGTACCATGTATTACAACATGCATATTATTTAGAAAAAAGGGT
AATTACACGTACGCCTTTAACAATGGGTGATGTGATCTAATTAACGGACTTAGTCACTTAGTCGA
AGAGTATGTGTCGAATGTCCTATATCGGTTGCACCATACACAAGGCACACCATTACTCATTTTTTG
CTCATGTGTAGCTCTTCAAATAAGAAAGACATAGGCATGGTTATATAACTTGCATGATACAATTGG
TTGTATAAATTATAATTAGGGATGTTAAAGGGCAGGACGGGGCAGATGTTAGCCTTTTCATCCCCA
CAGTGGGTCCAAAATTCATCCCCATATCCGCCCCGCGGGTATAAGTTCAAAACCACCCCGCCCC
ACCCCCCACCTAATCCCCACCCAAGACCGGCCTTCCAAATAGAACAATATTAATATTACACATTT
TTTGGTTATAATATAAGTCTATTACGGTGTACAAAGTATTATATTTACTATTTTTACTTTATTTTTTCA
TATAAAATAATATAAAAGTATATAATTGTATAATTAAAATTATATAGATGCATGTGGGTATGAGGTG
GGGACGAAGTGGGGCAGGTGAGGCCGGGTGGGTAAGAAGTAGGGCAGGGAGGGGATGTGGCG
GGTACAACCTAAAATCCAACCCCGCCCCATCACCCATGACGTGTATGGTTTTTATCCCCATCCCC
GCGCCGCGCCACCCACCAAATTGGGACCCATAACTACATCACTTGGGCAGGATGGGGCGGGTCT
TCTACTTGACCCGCCCCACTGACATCCCTATGTATAATATAATCCGTGTCATATGTGGCTCAAATTT
CAATTCATGCTATCTTTTAAGTTGGTTTGCATCCTAATTTATAAACACTTCATACATATATGTTCTTA
TATATAGACAACTGTACTTGTATCATTTAAGCACACATGCCAATACATATCTTTAACTATTTATCAC
TAATTTTGCATTTGTATGTGAGAAATATTTAACAAAATCTAACATGACAATATTATTTTACCTTACA
TAAAATCACAAGAGACCATTAGAGTAACTTATGCGATAAATTGTGTAAAAATCAAACGAATCATA
ATAAATTATCGAGCTAGCTTATCATAATACAAAGCCTTCATTAGTTGAAAATTCTAATGTAGTCTTA
TGATTTTTGACTACCACAATTTTTTAAAATGGACGGCTAAGATTGTTTGAATTTTGATTTATCTTTA
ATGAGTAATTTTAATTAAAAATTATTTTTTATAATTATTTTATATATATATATTTTAGTTCTAGAATTTC
AAAAATATTAAAAAATAATTTCTCGAAGATAAAAATATATTTAAAATAATTAAAAAATTATTTTTA
ATTAAAATTACTAATTAAAATAAATTAAATAAATAAATAGTCTCAACCATCCATTTTAAAAAAATT
ATGGCAGTCAAAAATCATAAGACTACATAAAAAGCATCCTGCATTAGTTGCATACTTGTACGTTA
AAAAACACACAATTTGCAATATGAGCAAAAATACAATAACCAAATTAGAAACCCTAAAAATCA
CTTTAATTCCACGATATCCAACACCATAAACATTTAGGCAACTATATATATATAGCTGTCCCCCTCT
ACTATATGTATACCTTAAAACCTAAACACATTTCCTTTTATCTCTACCCTATCTTTTTAACTGTTTCT
TCCCTTTCTCAATCACCAACATTCAGCAAGCAACTAACATAATCTATCTTATCCTCTCTTGTTTAAT
TAGCTTCTTCTTCTTCT

>CqYAB10

GAATTGGGGTTTTTGCTTTGGAGGGTATTTTGGGATTGGAATTGGTGGAGAGAAAGATGGCGGTG
GTTGTGCGGTGGTTGGAAGAGGGGAGAAGTGGTTGTGGTTGGGAAGTGAAGCTAAGATTGCCAT
TGAAGATAGGATATTTTTAGCGCCTTTTTTGTGGATTTGGGTAGGAGGAGAGTGGTTTATAAGTTT
TGGGTTTGTAATGTAACTAATGTTGGGCTATTGTCCTAAACTTTTGGGTCTTTTAGGCCCCTTTATTT
CTAGATGAGAAGAATTGTCGTATTGGACGTTTTTGTATGAAGATTCGTGTTAGCCGACCGTCTTTG
TTGTTGTCATCGTCGTCTTACTGGAATCTTGGAATCTCGCATTGGCAAACTAGACATGTTGAAAGT
GGGGGGAGGATAATATAGATTTTCAAACCTCAAAGGTCTAATAGGGATTGAACTTGCATCCTGAA
GGTTAAAAGGTACGATTTTTACTACAAGTATTAGTAATTGGGTGTTATGATAATAGAAATTGGATG
CACCTTTCATTATATGATGTTTATGGGATATTATTTAATATACTTTGTGACTTCTTTAGGTACATTATT
TGAGTAATTTGTGTTCGTTAATTAGGTTTGTGGCGCAACTGTTCTTGACAAAGAAAGCAAAGGAA
CATACCATATAACCATGCGTAGTGTTTTTTTGTTTGTTTATATATATAGAATTCACACCTACAAACC
AAGTTTGTAGTGCAGTCAGCAGTGTGCATTACAAATAGGTATTTATACCTCCATAACTATATTTTGC

CAAACACATTCTATGTGTATGTAGCGTATCATAATCCTTGCGTTGTTGTGCATATGAGATTTTATCC
AATCTTAGAGATTGGTATCATCTCTCTTGTCAGATACATTTGTGGTCAGCGACAAACTTGATTGCA
AATATTTTAAGTGCTCCATTATATATATTATACTAATATCTACATGAGAATACAAACTAGCAAAGCT
ACAATGCTAGTAGAGGCAATTGATTGGTCTAGTGAAATGAGAGCACCTTTGAATCATGAGGTTGG
AGGTTTAATTCCTACTAGTTAGAGAAAATTTGAGGGAAGGAGGTTCCCTTACCTTTGAATCATGA
GGTTGGAGGTTTAATTCCTACTAGTTAGAGAAAATTTGAGGGAAGGAGGTTCCCTTACCTTTTCTC
ACTCTCTTAAATTGTCTAGTCCGTCAACTTGGATCGAGTTGACCAGTGAGGGAGAACTGGCTCGA
CAGGAACTTTTTCTTTTTTATCTTATATACCCAAAAAAAAAAAAAAAAAAGTGACAAAATAATGAG
AAAAGAACAAATGATGTGACCACAAAATACAAGTCCAAAAAATTTTCTCACGTGAGATACACG
TGCAACAATAGACTTGGTTTTGCCTAGCAGAAATTGTAGGTAGTTGAGAATTAAATTTGGGGTAA
ATTTCAAAAACAGATAATAGAAAGAAACACAAAGAACAAGACATAAACCAATTAAGGTAAATA
TAGAAGGTAAAAAAATCAGCATTTTTCAAGTTTTCAACTCATTCATATATTTCATAGACTATGGTC
AATTATGCAGGTATAAGTATAACATTTCTTTGTTTCATGTAAAGACAAATCCTAAGATACCTCTTTT
CTATTTTTGTACACATTTCTAGAAACTTCATCCCCTCCTAAATAAGGTAAAAAATCATCTCACTAA
ACCCTAATTTAGAAATAATGACACAAAGATATAGGGAGGCAGAAAATAACACTTAACAAAATAA
GACATTGGTCCCCTTTTTCCTTTAATTTCCTACCATCAAACCAACAACATTTTGGAAATCTCTCTTT
TTTATGTGATGCCTACCAAGTGTGTCAATGTTTGGAACTTTGGAAGTAAGTGTGTGACTTGAATTC
AATGCATTTATCAGAACTCTAAGTGAGGTCGAAATTCTCATTTCTCAATTTTGAGAGTGTACTTGC
TTTTCAATCCAAGCAAGACTTCCAAAAATTTATGTGTTGGTTGAGTAAGAAATCTATGAATATAAT
GAAGAAGTGCTCCAATATTTTGGTGTGATGGTTAGAATTGCAAGGCCGACTAATAATGTTACCTAT
GACGCTAGAAAAATCATATGTTTTGATGAGTCATAAATCATTACAAATGTCGTTATCCCATCTTTTT
AAAGGGTTTATAGCTTCAATGAAATTTTTATGCCAATTTGGCATTTTACATATGTCAAAGAGTATTA
GACTAGAGTCATTCTTCATTTTCTTCATTAGTAATATACATTTTTGCTCGTTGAATGTGGAATTTTGA
TCAACCATTATCACTGCAAGTCTATTTTAAACATTTAGAGAGGCACGCTGATAAATTCAAATAAA
ATAAGGCACATGCATATATATACCAATCAATTTTGCGATGCTAGTAATTAAACTAGAGTACAAGGC
TTCTCAAATATTTGATCAAGATAGTTTTTAGTAGTATATGTGTGTTGTCCAAATGATGAAATAAAG
GTTAAGAGTTCGATTCTAATACAAAGTATAACCATTAATTAATCTGCTCCTCTTATTAATTAATTAG
ATCAACCAAAAGGAAAATTGGATTGCCTATAGATGACAATCAAACAAAAAATTGATTAAAGTAC
ATGATAAATTGGTGATAGCCAAATCTAACGTACCATATACATATAAGAATCATTACTTACAATTCA
CTGTCGTCCCTAGCTAAATTCTAAAAACACAGATTTTCATCATATAATTCATAATAATGTTGGATAC
ATTTATATCCTAGGAATAAATTAATATACATTATTATTAGCTACATAGATACTGGTATGTTAAAACA
CATGACTCACTTAAATGAAGTCTGATTTTTTTTTTCTATATATATTGTGCATACTTGCTCCTACTCTT
AGAACCCTAAAACCTACAATTACACAATTACATACTCTCACCTCCTCCAACCCCATTTACTCTCTC
TAAATTCTTGACCTTAGCTTCATCAAGAATA

>*CqYAB11*

AAATTATTATAGATTTCCTCAACTTGACTATCTTATAATCAGACATTTTTCTTTAAAAAAAAATTTGT
TAAAAGAACATTAAAACTCCAATAACTTAAAAGGTTTTTGTTCCTCATTTTGTTTTATTCCAATAAT
TGATTATGGATCGTTCGTGATATAAAATTAATCAGGTTTTCCATGATCCAAAATAATGAATATACA
ATCGAGTCTATTAAAATGAGATCGGATATTTGGATCCAATACATTTATGTCTCTTGAGAGGATAAG
GCATGTGAATGTGATCCCAAGACAAATAAGTCTTGCGAGGGAATGTGATGTATTGATATTGCTTAT
TAATGGTTATGAATTGATTAGTAACTTTATGGATTTGGTGGTTTCTTCTCTACTTGCATGATATTTTT
CAAATGGTTATACAAAACATTATATACTATACAAAACTTAATGAAACCTAGCTAACATAGCATATA
GGACCAATTTAAGATTACGAGAAGTTCCATTGTTACCACATCTGCAACTAATATCTATATCTGCCA

AGTAAATAATATTTGATTATTTCTTCAATTATTTGTTAATCGTTTAGATGTTAGTCTTTTCTCCATTAT
ATTGTTACCACATCGAGCAACTACTGCGCATGTAAGAGGCCTCTCCCTCGATATTTATCAACTTAG
GTGTTTCTTGACTAATATAACTATCTACAAATAATATACAATCGTACATACCAATGAAATCAAAAG
AAAGTGGTAAGGCTAATTATTTATTGTTTGTAGTTTGGAGCTATAGTGTTACTACTTTACAAGTCGT
CAGGTAAGGTATTAAAGTAATAGTGTAAACTTGCCCATATTATAGAAATTACAGCGGGTTGACCT
ATTGAAAAAAAGAGCCATATATTGTTTTATCATCCCTAAGATTAATAAATTAATAAATAAAGGACA
AAAAATAAAAAAGGGAGAAGAAAATAAGAACCACATAGAATTGAAGAAGCAAATAAAAGTAA
GGTGGGGTGGTAGCCCCTAATTAATAAGTATGAAAGCAACAAGTGAATGATGAAAACATTATGC
AAATGTCACTTTCTAAGTCTTCTCCCATATCTGCCGCTTCTGCTTCTTCTTCTTCATTTTCTTCA
TCTCTCAATTCACACAAATACATGCTATTCTATAATCTATGACTACTATACTTGGATACTTTGGGAA
TACCGCTCTAACCTTCACATACTTCTATGGATCCAACTTCTTACTTGCTTGTTACAATCCCTACATG
TTTTCACATACTCTCTATCCCATCCTTCTTCTGTTGTTTAGGGATCCCATGTAATCATGTAGTGTCTT
CTCTTCCAATCTTCACTTTTTCTTATGTTCTTGTCTAATTTCTCACCATTTCCCAATTTCTTAATTGTG
CGTTCTTTTCAATTGACGTCTTATGAACTTATCTGAAGTTATTAGATATGGTTGAAGCTTATTGAAC
CTAGCCGATTGGATCTTGTTAGATCGACCTAATTGCACCTTATAGTTTATTAGAATTGAATGAATTG
AACCCATTTACACATATATATTGGACTTTTTGCTTATTTTATAAGGTTAAAAGAGTGACACCTGGA
GATATATACACAATTTAATTTGAAATGGCAACATTTTTTTTTGTGGTCAGTTATCCCTTTAGTTAGG
CATAAGACTATTTGGAATGTTGAAAAAGGTTATCATGTATTCTGAATAGCATGCACTAGTTATGAT
TGGATTCAAAGATATTATGCAGAACAATGATTGTGTTAAAAAATCAATATTGTTAATTAATGAGTT
AGGACAATTTATATAATGGCATTAAGGATTCGCTAGAAATTTTGGCATACAAAGGAAAGTAGAAG
ATCGTATATGTCATTACTATTGTCCATAAAACCATAATAAGTGTCTTCTCTGACTATAAGCAACTAG
CACATCAGTTATAGACACATCTCTAACACTAATATAAATGAGTAAAAGGTACTGTTAAAACAATT
GTTACAAATCATGCATTAAATTTGGGATCAATCGGATAGTAGTCCAAAAACGTAGAGGTCATTTT
ATAGTGAGATTGAGTAAAGTATCTCGCATACATAATGTTAGATAAACCGAAGTATACACCATATGC
ATAACATTAGACCGAATGAATGAAGTAGTATACACCAGGGCCAAGCCATATACATAATGTTAATG
AACACAATGGAAGAATACATCACAATATCACATACTTCGTACTGGACAAGTGAATAATACGTCA
CATACATAATGTTTAGATCAATTAAAATTACGTGCTCATCACATACACAATCTTTGATTGAATGAA
GTGTACATTACATTCATAGCTTTAGACAGAATGAAGTGTACATCACATACATAGCTTTAGGCTGAA
TGAAGTGTTTGTCACATACATAACATTAGATAGAATAAAACATACATATATAAGTAAAAAAAATG
AGGTTTAATTTAATTTACATGACACATATAATGTTAGATAGAATGAGGTGCACACCACATACATAA
GGTACGTTTAAGAAATGAATCAAAGTACATGACATGTATAGTGGTAGACATGACATGCATTAAGA
TGATATACAAGTAGAGGGAAATGGGTTATAAAAGGAAACATAAAGGAACAAATTAGAGAATAG
GGATGAATGAAAATGAAGAAGAGATAAGCAAGTATCTACTTCTCTCTCTTATAATTAAGTAGTAG
ATCAAGAACAGCCCTTGAATACCCACTATCTAGTATCTACATACTTCTCTCTCTTCCCACTCAGAT
CACCTGTATTTGATTCTGAGAAGAAAATTAAAAACAAACACAAAGCTGAAAAACAGTACTGGT
AGTACTGTAATAATAAGCTGAAAAGAAAAAGGAAATTAATAAATAGAAAGGATCCAAGGTGTTG
GGGTTTCATCTGTCAAATCAAATCAAATCAAATCTGAGAAAATCAAAGGATTGATCAAATTTCAA
ATCAATTTAAAAAGAAAGAGATCTATCCAAGGAA

>*CqYAB12*

AGAAGAAAAGAGATAATTAAAAGGCCTCGGCCCCAGCCGAAGAAGATTACCATCCCTAAACTT
TCGGCTGAGGCCGACAGCTGCCAGCATATTCTCTTGGAAAACGTAGTGCGTATCGGGGAGCCAA
GTCTCCGGGATATGCTGCATTCGGCCGCTGCCTCCCCGGTTCGGCAGCTTGCCGAAGATGTTCTG
CTGATTAAGGTTCGGGTCTGGCCCGCTATCTTCGGGAGGACCTAAACTTCCCATATCAGGATCCG

GCTCGGCAAGATACCGGGGAGCGCTGAAAACGTAAGGGTGGCTCGGCTCCGTGGGTGCCCTAA
CCCAAATCCATTGACCTCTCCAGTCCTTGTCTGAAGAGTCGTTCGGCCACATGGTTAGGAACGGG
GGCCGAGACTCAATCCGATACCACCCAGAGCCATGGCATCCGTGCCCACTTTGAATGTTTGGCA
CTAGCCGATGGAGCCTTTTGAAGCAATGCAGGTTCGGCACGTACCTGAAGAATTTGCAGACCCA
TAAGTACGTCACTACCCTTCGGATGCTGGTGGGGGTCAGCTGGCACAGGGAAATGTTGTAATGG
ATCAGCAAGTCTCGGACGAAAACGTCCAAAGGGAACCTCAGCCCATACTGAAGCTGTCTCGTGT
ACACCCCCACATACCCCGGGGGTGGACGATGGATTCTATCGGATGCTGAGGGCATTCGGCACCA
ATATCCATCAGCTTCAAAAATGCCGTAGTGCAACTCAATTTGCTCGGCAAATCGATATTGATCGG
CGTTCTCTACCCACCATGAGTAGGGGCGGTTCTTTATCATGGCCGAAGCGACTTCGGCATTCTCC
CACTCTGAAAACCCAGGGAGGATATCCCAAAGGACAGCGGGAATTTCATCGTCCCGTCCAGAA
GCACTCGGCTCATCCGTTCTTTGAGGAGGAGCCGAGGAAAGGGGAGTCGCCCTAATCCCATCAG
CAGGGTCAGACTCCGGCCTTCGACCATTCCAGGTCCCAATCCTATTCCTAGGATCGGGAATCGGC
CTTGCTCTTCTTGGAGCACGGGCTTTACCTCTAACTCCTCTTGCCATGGACTATTTCGAAGGAAAA
AGTCGAAAAAGGGGAAAGAGTGCAAAGTCGAAGAAAAATGAAATAAAATCAAAGGAATTTAC
CTTGATCTGAAGCAAAAGTCGCCGATTTACGAATTGGTAGTGACAAGTCTCAGCTTCTAAGCTTA
ATGCTCGGGGAATTTGCGAAGTTTTGATGCGGGCTCTTGAGCGTTCTTGAGAATTCTGGGTTTGTG
TTGATGAATGAGTGGGGAGAGAAGACGCGCCTCTATTTATATCTCCAGAAATGGAAATTCAAGG
AGTCTGCGTTTCAATTTCTCTCAAGCACCCCACTAAGTAACCGTTAAACACACGACCTTGCAGTT
AAATCGCCCACTACCCGTCCAACTTCCGCAATTATCCACATTAACTGTTGTTATCTCATTAATCAT
GGGTTAATTACGGTTTAAATTCACATTTTTGGATTAACAAATCATTCACTTCCCTCCTTTTCTGATT
AAATCAAACCCGACCCGTCATCCTTCCAAATCGGGTCTGGGGGGCAAGTTGTTTGGGTCCAATTT
AGTCCAATTGGGCCTGGTCACTGAATGATCCAATTAAAAGCCCAAGGTTAAGGTTCAACTCTCC
AAATGTGCTAAAGTCAAAAATCAACATCCTCAAAGCCATGTCAAGGTCAGTCAACGGCCCAATC
AGCTTGGCCCAGCCCAACTGACTTCCGGGCCCAATCCGCCTCCTTCATTAGCCAAGGTATAAAA
GCCAACAATTTGGTCAATTCTTGGTAAGACAATAAAGCCTCAAGCAACTAAACTCTTGCACAGC
TTTCTCTCTCTAATATACTGACTTAAGCATCGGAGGGGCCTTCCTCGGCGTCCCCCCCGAGGCTAG
TTTACGTGTATTTGTTGTGCAGGAAACCCTCGGCTCCTGCGTCGTTCGGACCGAGACCAAGTTCC
CTCAAGATAAAAGGATCTTCCGAGATTCGATTGTTTTCATCCGAAACAACTTTTACTTTGTATTTC
TCATGTAAGAACCATTAGAACACGGACAATAAGAGAGGAGTTTTTGAACTTACGCAGTAATAAC
CATTAGAACATGGACAATAGTTAAGAAAAGTGAAATAGAAATACACTCATTTTAATGGAAAAAT
GTACATGTTTTTGTCAATAAATGTGATTTTATTAACTAATTAGTCTTCGATATTAATTTAAAAGGCA
AAACCTAACGAACACCCCGGAGGCGTTAGATAAGCTAGTTAAAAGTCTAAAGATTATTAAATTA
AAATAAACATTAATTATACCGATTGACACGTAATGTTGTCATGCTTTCCCAAATTAATACAGCTTT
AGAACAACTAATTTTAAAATATATAGTTCAAAGTAACAATAATTTAAATTTTGATAGTTTAATGAT
GTAACCCTAATGTGTTGGTATACTTTACCATGTAATTTCTTTTGCTTAAATACCTATCTTCACATTAG
AACACCTAATAATCAATCTGGGAAAAATACGACTAATCCTTAGTTATTGTCCCTCGTGTGCATCCA
ACCATTGTTTTGAGAACAGGAGAGGACCCGACCCCGGCCCCAAAAAAACAAGGCCTTGACTAG
GCACTAGGTTCCTATATGGCTAAACATCAATTAAAATCCCTTATCACATCCATAAGCTAATTAAAT
TGTACGACTCCATCCCACTTTGTAAGATCCTTTCCCAACCGACCAGAGTCTTATGTATGGACTATA
GTCTGTTACAGCTTATTTTACTTCTTTTTTTTTCTTCTCTTTTTTCATTACATTATCTTTCTCTCTCCTC
CTCTTTTTTCCCTTTCTTTCTCTATCTTAGATTGATCAAATAAATTTCAAGTAATAGCACAAAATAG
TGAATTTTCTTGTCTATTATATACTTGAAATTTTAATAACTTTTCAAAAAAAAAAGTAAAAAAATC
AGAGATAATCG

Supplementary File S3

Genomic sequences and coding region sequences of *CqYAB* genes

CDS

>*CqYAB1*

ATGTCATCAACAAGCTGTGTGGATCAAGCTGCTGTTGCTCCTCCTTCATCTGAGCAACTTTGCTA
CATACCTTGCAATTATTGCAATATTGTTCTTGCGGTGAGTGTTCCATGCAACAACTTGTTCGATAT
AGTAACCGTCCGTTGCGGGCACTGCACTAATCTATGGTCAGTTAACATGGCCGCGGCCTTCCAT
TCGCTCTCCGCATCCTGGCAGCAACACCAACAACAAAACTTTCATCAGGCACCAAACAATGGC
AATATGGGTGAATATAGGATTGACAATTTGGGTTCATCCTCCAAGTGCAACTACACTAGCAAAG
CAGCAACAACTATGCGAATTTCGCCTCCTATTAGCAATAATTCTTCTGAGGAAAGGGTTATTAA
TCGCCCACCTGAGAAGAGGCAACGCGTACCATCTGCCTACAACCAGTTCATAAAAGAAGAAAT
TCAGAGGATCAAGGCTAATAATCCTGATATTAGTCATAGGGAAGCATTCAGTACTGCTGCCAAA
AATTGGGCACATTTCCCTCACATTCATTTTGGGCTGATGCTGGAGACCAACAATCAACCTAAGC
TAGATGAGGGCTCGCAAAAGCATCTCATGCCAAGGACTGCTTTACTAAACAATTGA

>*CqYAB2*

ATGTCATCCTCTTCCTCGTCAACTACGACAGGAAGTGGTGGTGGTGGTTGCGGCGGAGGTGGTAGCT
TAGATAATAACTACCATCATCAAACACCTAATAACAACAATAATGTAGAACATGTTATTTCGCCGTC
GGATCAACTTTGTTATGTTCAATGCAATTGCTGTGAGACTGTTCTTGCTGTGAGTGTGCCGAGTAGCA
GCTTGTTTAAGACGGTGACGGTGAGATGTGGACACTGTACTAGCTTGTTGTCTGTCAATATGAGGGCT
CATCTTTTGCCTTCGCCTTCACCTCCGTCTCCGCTTCCTCCGCCTTCGCCTAATCACCTTCATCTTTCTC
CTCCCTCTTTCTTTACCTCTCCTCACAATCTTCTGGAGGAGATTCGGAGCTCAACACCAAATATACTT
ATCAACAATCATCATCAGCCTATGTTCAATGACCCGATGATGTCGGTTCGAGGTGTTGATCATCTTCA
TCACCACCATCAAGAGATCCCTAAACCCCCTCCTGTCAATCGCCCTCCAGAGAAGAGACAGAGGGTA
CCATCTGCCTATAACAGATTTATCAAGGACGAAATTCAACGAATCAAAGCCGGAAATCCTGATATTA
GCCACAGGGAGGCCTTCAGTGCAGCTGCCAAGAATTGGGCCCACTTCCCACACATTCATTTCGGCCT
TATGCCTGACCATCAACCCGTGAAGAAGGCTAACGTGCGCCAGCAGCAGGAAGGAGAGCACGATCA
AGTTATGATGAAAGAAGGGTTCTTAGCTCCTCAAGCCAATGTGAATGTGGGTGTAGGTCCGTACTAA

>*CqYAB3*

ATGTCATCAAGCTGTATGGATCAAGCTGCTGTTGCTCCTCCATCATCTGAGCAACTTTGCTACAT
ACCTTGCAACTATTGCAATATTGTTCTTGCGGTGAGTGTTCCATGCAACAACTTGTTCGATATAG
TAACCGTCCGTTGCGGGCACTGCACTAATCTATGGTCAGTTAACATGGCCGCCGCCTTCCATTCG
CTCTCCGCATCCTGGCAGCAACACCAGCAACAAAACTTTCATCAAGCACCAAACAATGGCAAT
ATGGGTGAATATAGGATTGACAATTTGGGTTCATCCTCCAAGTGCAACTACACTAACAAAGCAG
CTACAACTATGCGAATTTCGCCTCCTATTAGCAATAATTCTGCTGAGGAAAGGATTATTAATCGC
CCACCTGAGAAGAGGCAACGCGTACCATCTGTCTACAACCAATTCATAAAAGAAGAAATTCAG
AGGATCAAGGCTAATAATCCTGATATTAGTCATAGGGAAGCATTCAGTACTGCTGCCAAAAATT
GGGCACATTTTCCTCACATTCATTTTGGGCTGATGCTGGAGACCAACAATCAACCTAAGCTAGA
TGAGGGCTCGCAAAAGCATCTCATGCCAAGGACTGCTTTACTAAACAATTGA

>*CqYAB4*

ATGGCAACACTTAATCGTTTATTTGATACTCAAGAACAAATTTGCTACGTCCAATGCAGTTTTTG
TACCACCATCTTACTGGTGAGTGTGCCATATAGTAGTATGACAATGGTGGTGACAGTGAGATGT
GGTCATTGCACTGGTCTTCTCTCAGTCAGCATGTTGAAAGCCTCCTTTGTTCCCCTCCATCTTTTTT
CTGCCCTTAACCAAGATCAGGTGAATTTTAAGCATCAAGAGGAAGTGGATACATCAAAGGCGC
ATATGGACAGGCATAGTACTACTTTGTCCATGTTACCCTCTTCAGAAGAAGATAATGACGATGA
GGAGGAGGATGAAGAAGAGGATGAAGATAACATTGCGCTTGAACAAATTGTTAATAAACCTCC
AGAGAAGAAAAGAAGGGCACCTTCAGCTTATAACAAATTCATCAAAGAAGAAATCAGGAGGT
TGAAGGCTAGGAATCCTAACATGACTCATAAGGAAGCCTTTAGCACTGCTGCTAAAAATTGGG
CTCATTTCCCGTCGGTCCAACATGAAGTTGATGAAGATAATGATAGCTATGAGAACAGCATGAC
ACAAAACTTAGACGACGAAGATGAAGCAAATTGA

>CqYAB5

ATGAACACCAACACGATGGAAGACAAAGTGGGCTCGGAGTTGGCTCCACCACCTCCGGAACAT
CTTTGCTATGTTCGGTGCAACTTTTGCAACACTGTCCTCGCGGTTGTGATTCCATGCAAGAGGTTG
TTGGACACGATAACAGTGAAATGTGGGCATTGTAGTAATGTATCTTTTCTGAGCACTAGGCCTCCT
CTCCAAGGGCAATGTCTTGACCACCAAATCACCCTTCAGGGGTTCAATTTCTTGGAGAAACCAG
GGGGTTTTTGCAGCACCATTGATCATCACAGCAACAAGAAAAGTGAACCATCTCCTTCGTCGTC
ATCCACCTTAACCGAGCCTGTTTCTCCAAGGCCATTTGTTTGTAAACCTCCTGAGAAGAAGCATA
GGCTTCCATCCGCTTATAATAGATTCATGAAGGAGGAGATTCAGCGCATCAAAGCAGCAAATCCT
GAGATACCTCATAGAGAGGCTTTTAGCACAGCCGCAAAGAACTGGGCAAGGTTTCTTCCGCACA
CTCCAGCTGGGTCACTTGCGGAGAGCAGCAACCCCAATTAA

>CqYAB6

ATGGAGTTCAGTACATCAGCAGAGCGAGTCTGCTATGTCCACTGCACCTTCTGCAAGACCATTT
TAGCGGTAAGTGTACCATGCTGCAGCATGTATAACATGGTAACAGTCAGATGTGGGCATTGTGC
CAATCTTCTCTCTGTTAACATTGGACTTTCACCTCCATCTATGCCTCATCAAGATAATTTCCAGTT
GCTGAGGCAGCACTGTAATTATCAAGATGTGAGCAAGGATAGTAGTAGTACTATAACAGGTGG
CTCATCTTCTTCAACTATTACAACAATGGCTTCTGATGATCATGATGTTCAACAGACTCGTCCCC
CACCCATTCGTCCCCCAGAAAAGAGGCAACGCGTTCCTTCGGCTTATAATAAATTTATCAAGGA
GGAAATCCAAAGGATAAAAGCCAGCAATCCTGAAATTAGCCATAGAGAGGCCTTTAGTGCAGC
AGCTAAGAATTGGGCACATTTTCCTCATATTCACTTTGGCCTAAATCTGGATGGGCAAAGGCAA
GAAAGGTTGGACCAACCAGTTTCTGGAGAAGGAACAAACAAGTCTGATGGATTTTACTGA

>CqYAB7

ATGTCGTCCTCTTCCTCGTCAACTACGACAGGAAGTGGTGGTGGTTGCGGCGGAGGTGGTAGTTT
AGATAATAACTACCATCATCAAACTCCTAATTATAATAACAATGATAATGTAGAACATGTTATTTC
GCCGTCGGACCAACTTTGTTATGTTCAGTGCAATTGCTGTGATACTGTTCTTGCTGTGAGTGTGCC
GAGTAGCAGCTTGTTTAAGACGGTGACGGTGAGATGTGGACACTGTACTAGCTTGTTGTCTGTCC
ATATGAGGGCTCATCTTTTGCCTGCCGTGCCTTCGCCTTCGCCTCCATCTCCGCTTCCTCCGCCTCC
GCCTCTTTCGCCGAATCACCATCATCTTTCTCCTCCCTCTTTCTTTACCTCTCCTCACAGTCTTCTG
GAGGAGATTCGGAGCTCAGCACCAAATATACTTATCAACAATCATCATCATCAGCCTATGTTCAA
TGACCCAATGATGTCCGTTCGAGGAGTTGATCATCTTCATCACCACCATCAAGAGATCCCTAAAC
CCCCACCCGTCAATCGCCCTCCAGAGAAGAGACAGAGGGTACCATCTGCCTACAACAGATTTAT
CAAGGACGAAATTCAACGAATCAAAGCCGGAAATCCTGATATTAGCCACAGGGAGGCCTTCAG

TGCAGCTGCCAAGAATTGGGCCCACTTCCCACACATTCATTTCGGCCTTATGCCTGACCATCAAC
CCGTGAAGAAGGCTAACGTGCGCCAGCAGCAGGAAGGAGAGCACGATCAAGTTATGATGAAA
GAAGGGTTCTTAGCTCCTCAAGCCAATGTGAATGTGGGTGTAGGTCCGTACTAA

>*CqYAB8*
ATGTCAAGCTCTAACACTGCTGCCTCTTCAACAACAACAAGCTTGTCATTGGACCACTTCTCACC
TTCTGAGCAACTCTGTTATCTCCAATGCAGTCGCTGTGAAACCGTCCTAGCGGTAAGTGTGCCAT
CAAGCAGCTTGTACAGGACGGTGACGGTCCGCTGTGGGCACTGCACCCATCTCCTGCCGGCGAA
CACACGATCTTTACTACTTCAGCCACCGCCGGCTAGTCAGTATCACTTGCCTCATCATCATAACTA
CTACTCTCCCAACTCCCATTATCGTCTGGGGGAGATGCCAAATCAAGCACCAAATTTCTCACTAA
CACAACCAAATGGTGCATCTAGCTACATGAATCCATCATCAAGCAGAGGCGGTCCAAACGAGCT
TCCGAGGGCTCCTACCACTAACAGACCTCCGGAAAAAAGACAGAGAGTGCCTTCAGCTTACAA
CCGATTCATCAAAGAGGAAATCCAGCGTATTAAGGCTGAAAATCCTGATATTTCTCATAGAGAGG
CTTTCAGTGCTGCTGCCAAGAATTGGGCCCACTTTCCCCACATCCAATTTGGGTTGATGCCGGAA
CGACGGTGA

>*CqYAB9*
ATGATGAACACCAACATGATGGAAGACAAAGTGGGCTCGGAGTTGGCTCCACCACCTCCGGAA
CATCTTTGCTATGTTCGGTGCAACTTTTGCAACACTGTCCTCGCGGTTGTGATTCCGTGCAAGAG
GTTGTTGGACACGATAACAGTGAAATGTGGGCATTGTAGTAATGTATCTTTTCTGAGCACCAGG
CCTCCTCTCCAAGGGCAATGTCTTGACCACCAAATTACCCTTCAGGGGTTCAATTTCTTGGAGAA
ACCAGGGGGTTTTTGCAGCACCATTGATCATCACAGCAACAAGAAAAGCGAACCATCTCCTTC
GATGTCATCCACCTTAACCGAGCCTGTTTCTCCAAGGCCATTTGTTTGTAAACCTCCTGAGAAGA
AGCATAGGCTTCCATCCGCTTATAATAGATTCATGAAGGAGGAGATTCAGCGCATCAAAGCAG
CAAATCCTGAGATACCTCATAGAGAGGCTTTTAGCACAGCCGCAAAGAACTGGGCAAGGTTTC
TTCCGCACACCCCAGCTGGGTCACTTTCGGAGAGCAGCAACACCAATTAA

>*CqYAB10*
ATGGCAACACTTAACCGTTTATTTGATACTCAAGAACAAATATGTTACGTTCAATGCAGTTTTTG
TACCACCATCTTACTGGTGAGTGTGCCATATAGTAGCATGACAATGGTGGTGACAGTGAGGTGT
GGTCATTGCACTGGTCTTCTCTCAGTCAACATGTTGAAAGCTTCCTTTGTTCCCCTCCATCTTTTTT
CTGCCCTTAACCAAGATCAGGTAAATTTTAAGCACCAAGAGGAAGTAGATACATCAAAGGCTA
TGGACAGGCACAGTACTACTTTGTCCATGTTACCCTCTTCGGAAGAAGATAATGACGATGAGGA
AGAGGATGATGAAGATAACATTGCAATTGAGCAAATTGTTAATAAACCTCCAGAAAAGAAAA
GAAGGGCACCATCAGCTTACAACAAATTCATCAAAGAAGAAATCAGGAGGTTGAAGGCTAGG
AATCCTAATATGACTCATAAGGAAGCCTTTAGCACTGCTGCTAAAAACTGGGCTCATTTCCCGT
CGGTCCAACATGAAGTTGATGAAGATAATGGTAGCCATGAGAACAGCATGACACAAAACTTAG
ACGACGAAGATTAA

>*CqYAB11*
ATGTCAAGCTCTAATATTGCCGCCTCTTCAACAACAACAACAACAACAACAACAACAACAAGC
TTGTCATTGGACCACTTCCATCCTTCTGAACAACTCTGTTATCTCCAATGCACTCGCTGTGAGACC
GTCCTTGCGGTAAGTGTGCCATCAAGCAGCTTGTACAAGACGGTGACGGTTCGATGTGGGCACT
GCACGCATCTCCTGCCGGTGAACACACGATCTCTACTACTTCAGCCGCCGCCGGCTAGTCAGTAT
CACTTGCCTCATCATCATAACTACTACTCTCCAAACTCCCATTATCGTCTGGGGGAGATGCCAAAT

CAAGCACCAAATTTCTCACTAACACAATCAAATGGTGCATCTAGCTACATGAATCCATCATCAAG
CCGAGGCGGTTCAAACGAGCTTCCAAGGGCTCCTACCACTAACAGACCACCGGAAAAAAGACA
AAGAGTGCCTTCAGCTTACAACCGATTCATCAAAGAGGAAATCCAGCGTATTAAGGCTGAAAAT
CCTGATATTTCTCATAGAGAGGCTTTCAGTGCTGCTGCCAAGAATTGGGCCCACTTTCCCCACATC
CATTTTGGGTTGATGCCGGAACGACGGTGA

>*CqYAB12*
ATGGAGTTCAGTACATCAGCAGAGCGAGTCTGCTATGTCCACTGCACCTTCTGCAACACCATTT
TAGCGGTAAGCGTACCATGCTGCAGCATGTATAATATGGTAACAGTCAGATGTGGGCATTGTGC
CAATCTTCTCTCTGTTAACATTGGACTTTCACCTCCATCTATGCCTTATCAAGATAATTTCCAGTT
GCTGAGGCAGCACTATAATTATCAAGATGTGAGCAGAGATAGTAGTAGTACTATAACAGGCGG
CTCATCGTCTTCAACTATTACTACAATCGCTTCTGATGATCATGATGTTCAACAGACTCGTCCCC
CACCCATTCGTCCCCCGGAAAAGAGGCAACGCGTGCCTTCGGCTTATAATAAATTTATCAAGGA
GGAAATCCAAAGGATAAAAGCCAGCAATCCTGAAATTAGCCATAGAGAGGCCTTCAGTGCAG
CAGCTAAGAATTGGGCACATTTTCCTCATATTCACTTTGGCCTAAATCTGGACGGGCAAAGCCA
AGCAAGGTTGGACCAACCAGTTTCTGGAGAAGGGACAAACAAGTCTCATGGATTTTACTGA

Genomic sequences
>*CqYAB1*
ATGTCATCAACAAGCTGTGTGGATCAAGCTGCTGTTGCTCCTCCTTCATCTGAGCAACTTTGCTAC
ATACCTTGCAATTATTGCAATATTGTTCTTGCGGTTGTTATTATTACTATAATCTCTTCATTTTCTACC
TATATTTTCACTCATTAATTTTCTTTTATCTATTTATTACTTTTTTTTAAAAAAAAATTTATTAGATTTG
GGTTTTAGGAAAATTTCTAATCTTTAAATATATTTTAAAATTGTTGTACGGTATAAATATAGTCAAG
TCTCAATCGTTTATGAGACCATTTTGGTTCTCCCTTCTTTTGATTTCTTCTTCTTTTACAGTCAAAGA
AAAGTCACTCTTTATTCAGAGCTCTTTTCTTCTACCCTAATCACCATAAATATTTGCAAATTACAA
ACATATTCTTTCGATTTTCAAATGTGCCCTAAAAAACCCGGTTTAACCTTTCAGGGATAGATAGGA
TAAGACTGTATACATCGCAATAACTGTAAACTCGATTTTAATTATCGTGTTGTCATTAGTGTATTTC
TAAAGAGTTCAATTTATTTTTCATAGTAATGAATTGTATCTAGTTGAGTTTTATTATTTTATTTTATTT
TTTAATTTTTTTTAATGTTGATGGATCATGTGTGTACATTAAACAGGTGAGTGTTCCATGCAACAAC
TTGTTCGATATAGTAACCGTCCGTTGCGGGCACTGCACTAATCTATGGTCAGTTAACATGGCCGCG
GCCTTCCATTCGCTCTCCGCATCCTGGCAGCAACACCAACAACAAAACTTTCATCAGGTATTACA
TATACTCACTCTGTCTCTAAAAGTTCATCGGTCTCTTTTTAATTTTTTTCATCATATAACCATTGTCT
AAACATAATTTTGTTATTAATCTACTTATACACCTAGGGTTTTCAAATTAACGTTGAGTAACACATT
TCTCAAACTTGTCTTAATTTTATTTTTATTATTATTATTATTATAAAATTATTCTATGTGTATATTTTTG
GGAAGTGGGTGGATTATTAGATAATTTTATTAAAAATTATAATAGATAAATAATTTAATTTGGGAA
AGTCTAGGTCTCTTGGGATTTGTATTTATAGGTAAGACCTTTTTGTTGATTTGATTGGTAAAATTTG
TTAGATCCTGCAGGTGTTTTTTTCTTCAATTGTAGGCTTAGCACACTCTACACATATATAGGTCTAT
TTTTATTAATTAATAGTACTCCTTATTTACCTAAGCTAGTCTAGCTCTATGTCTTATAAAAAGTCTAG
TATGCTACAATTAGATTACAAAAAAATACTGTAACAATAATAATAACATAAATGTTAATTTATAGG
AGATCTCTCTATAATTTTCTTCTTGATCTATTGTAATAATTTTCCTTCTTTGAGTTACCTTGTTTAATT
TGTGTCTCTTCTCGTCTCAAACGCCAAGATAACACATCATATATTCTGCAACGATGATGACTAGTA
TGCTCGATAATTACTTGAGCGGGTTAAGCCCTATTTTGTTTTTTATAAAGAAATTGTTGCGGATAAT
GTTTTTATCATATTGATTTGATTATACAAAGAGCGTTTACACTTGTCGATTCCTAAACTCTCTACTA
CGCTGACATGGATTATCAATTAACATCTTATTAAACATATACTAGTCATATGTTAAGCATATGTTAA

ACATTGATGATTAGTCTATTATGTTATTAATGTTTTTTTTTTAAAAATTAATCATAACATTTATCTTTC
ATTAAACTTCATTCATTTATGTTTTAGGCACCAAACAATGGCAATATGGGTGAATATAGGATTGAC
AATTTGGGTTCATCCTCCAAGTGCAACTACACTAGCAAAGCAGCAACAACTATGCGAATTTCGC
CTCCTATTAGCAATAATTCTTCTGAGGAAAGGGTTATTAATCGCCGTAAGATTAATTTTCTTCCATT
TAATTCAACATTAATACAACTATTAGTTTTTACAATCTTATGTTTGATGATTCAAATCATTAATTAAT
TTGATTGCAACGTTTTTGTTCATGTATTTAGCACCTGAGAAGAGGCAACGCGTACCATCTGCCTAC
AACCAGTTCATAAAGTAATTAATCGTACACTTAAGATCAAAGCTAATATATCAATATATGTATATC
ATAAATACAGTACAATGCATTTAATTTAGTACTAGTACTCTTAAATTAATGAATTTGTTATTTGTGA
AATTTATGCAGAGAAGAAATTCAGAGGATCAAGGCTAATAATCCTGATATTAGTCATAGGGAAGC
ATTCAGTACTGCTGCCAAAAATGTGAGTCCACCCAACATTGTCAACTTATGTGCACCATTTTTCA
GTGTCGGTCCAACTTTGAACATTAATATCTCGATCTCATAACATATCCAATAATTAAAATAAAATG
ATATTTTTAATTATAGGTTAAATTTCGATCAAGAATGTATATATAAAGTGTGAGAAATATACTTTGA
ATAATTATAACTTAATTTTATTTTGTTAATTGTAGTGGGCACATTTCCCTCACATTCATTTTGGGCTG
ATGCTGGAGACCAACAATCAACCTAAGCTAGATGAGGTAATTTTTCACAGCTATTTACTTATTTAG
TCCTGCATTTTTCATTAAATTAAGAACATTATACACGTATAATTTTAAGCAACACCATAATCATATA
CGGAGTAGTATTTTAATTTTAATTGAAACAATCTTAATAATATAATTCACGCATATAGTACAACATA
GAATCCCTAATATTCTCTAATATGGAGTATAATTTATCTTAGAGAATGTAAAATGCAATATTGGTAA
AGGAAGAATGGGAGATGAATTTGAACATAAAAAAATAAAAATAAAATTAGACAATGGATAAAG
AAACTTAAATTATGTAATAGAAAAATGTTCTAGGAGCATGTCATACATTTATTGTCAAAAAATGTT
TAGTTAAGCTGAAAAATAACAACAAAAACATGGATCATTTATTGTCAATTGTTTAGTAAAGATGC
ATGATTATTTTTCTTTTAATTTGCTCTTCATTTAATACTTGTATCACTCTTCTTTTGTTGAATGTTTGT
GGCATTTATAATTAATTTGATTAAGTTTCTTATAATTCAGTGAAATCGTTCAATTCACTAAAACTCT
TGTGGTTGATCGCGATTTTAGGGCTCGCAAAAGCATCTCATGCCAAGGACTGCTTTACTAAACAA
TTGA

>*CqYAB2*

ATGTCATCCTCTTCCTCGTCAACTACGACAGGAAGTGGTGGTGGTGGTTGCGGCGGAGGTGGTA
GCTTAGATAATAACTACCATCATCAAACACCTAATAACAACAATAATGTAGAACATGTTATTTCGC
CGTCGGATCAACTTTGTTATGTTCAATGCAATTGCTGTGAGACTGTTCTTGCTGTAAGTATAATTGT
TATCATTATTTAATTAGTTGTTATTAATAATTTTATCTATTAAATGATGTGTTGATTGTAGGTGAGTGT
GCCGAGTAGCAGCTTGTTTAAGACGGTGACGGTGAGATGTGGACACTGTACTAGCTTGTTGTCTG
TCAATATGAGGGCTCATCTTTTGCCTTCGCCTTCACCTCCGTCTCCGCTTCCTCCGCCTTCGCCTAA
TCACCTTCATCTTTCTCCTCCCTCTTTCTTTACCTCTCCTCACAATCTTCTGGTATGTTTTTTTTTTTT
TATTATTATTAATATTAGGGTTCTTCTTTGGTTATTAAGAGACTAAAAAATTAAACATAATATTTTCT
ACTTTCTTCGTTGGTACAAACTAATTTAATAGTTAAGCTATGCCTTCATTTCCTATGAAATTGATGG
AGCGAGCTAATAATGTGACAACTAAATCTGGATAAAGGTAGGCACACTCTAATTCGTTAGTCCTT
TTTTGTGTGGGTGGTGATGGATTGTGAGGAAAACTTGGGGTTGTTATGATATGATTAATTGGTAAC
AAAAAATGCGAATTTTAACCTACAATCAACTAGAAGTTGTCAGTGTAGATACTCTTGAGATTAAG
ACACATTAGTAGACAGTACTTGCTATTAAAATGAACAAATTTTTTATAACTAATGTTGAAAAATGG
ACTTAGTAAAATTTTATTTGAGTTTTGAGGCTTATAAAAAAGTTGACATTTGTGTGTTCCATGTGTA
TGTATTAATAGGAGGAGATTCGGAGCTCAACACCAAATATACTTATCAACAATCATCATCAGCCT
ATGTTCAATGACCCGATGATGTCGGTTCGAGGTGTTGATCATCTTCATCACCACCATCAAGAGAT
CCCTAAACCCCCTCCTGTCAATCGCCGTAAGTCGTCCATGCTCGAATTGACCATTCATATATAAAA
TCCTTTCTACTCTAACCTTATAATTCTTCCAATTTTTGTATTTACATAACAACGAACAATAATAATAT

CAAGTCCATAATCCCAATGAAGTATTTGTATTTTCTAGTAACACTTAAAAAAGACAACATTTTATT
CCGCAGTTGTGCTTTTAGCCCTAAATTTGATTGATCACCCCAACAGTTTTTGGTACATCACTTTACT
TACAAAATTTGAGAAAAGAAGTGTAAAACAAAATAAAGTGTTGTTTCTTTTTCTCTTCATTCATC
ATTCATCACTTTCAACTAACTGTTGTACTTGAACTCTTTTTCATTTAGACTTTGTATCATACACGTTT
TTCTCTGCACTCAAATTCTCCTCTATATTACATGTACTCGTTAACTTATGTACTTTTAAATACTCGTG
TAAATAATCAGAATTAACTACGAAATAACTTATACTCGTAGTATATTGTTATGTACTAATCATCTTT
TACTAGCCTTCTCAATATTGTGGAGGAAATATTAACCTCAAAGGCATAGTATTCCAAAAGACCTAT
TTAGTAATACCATTGTCTAAATTTTTTTTTTTTTAGTTAGTTACTCCTTAATTAATAAGATTAGTGTTA
AGGTGTAATTAAGTTTCAACCTAAATTATAATATTCCTCGGAGTAATATAAAATTCTAGCCAAATT
GAGCTACTTGCCCATCACTTAGAAGGAAAAGAAAAAAAAATATTATACTGTTTGATTAGTTAATT
AGTAAAATTTCTCACATAATAAAGAGAAATAGGCCTGTAATTAAGGGACGGGGAGTGTATTACTT
TATAATGATGATAGACCAGTGAAATTTTGATTGATTTAGGAGTAAACTGAGCGAGCCAAAAGAC
AAAAGGGCAATTATAAAGTCATCATTATTTTTATAGTTAGGTTAGCCAAGTGTATGTCCCCCACAA
CCCCTTGAGCAGGGACATATAAAATCCAAGATAGAAGAAGCGGTTGATGATGGGATAGATTTGA
TAGATACAATGTTAACAGAATGGTATGATGAGGTTATGATTTTGAATTGATAGAGAAAGGGATTG
GAATGAATGAATTTAATGCAATTTAATTTTATAAAAAATGAAAATAAAATAAATTTAAGTTGTCAG
AAGAAAAAAGGTGAGTTTAATTAATGTGGTTTTGTTTGTCTTGTTTGTTTATATGTGTATATTTTTTT
TGCATGTTTATACAATGAACAGCTCCAGAGAAGAGACAGAGGGTACCATCTGCCTATAACAGAT
TTATCAAGTTAGTACCCTTTTTTCCTACCTTCTTATTTATTTCATTAATTTAATTATGTTAGTACTTTAT
GTCCAATTTCGCAAATTGTAGATGAAAATAATTAATTGATATTGTGTCCGTCCAAAATGTATAGCC
AGGTTACTATTTTATTTAAGCTAAGTTATATAAAAAAAAAAAATGTATTAACATGATTCATCCGGAT
AAAAGAATAATTAACGCTGATTACTAATGAAAAGACATGTAATGGTTTTATAGGGACGAAATTCA
ACGAATCAAAGCCGGAAATCCTGATATTAGCCACAGGGAGGCCTTCAGTGCAGCTGCCAAGAAT
GTAATGAAATCTTTGAAGTTTCTATGCCTGTTGGTTTTAAGTTTTATTATATAAATGACTAATTTTAG
AATTTGGGTATTATTCAAAAATTGCTCAAAGATTATTGATTTTCTATTTGTTTTGCATGTAAAAAAG
TGGGCCCACTTCCCACACATTCATTTCGGCCTTATGCCTGACCATCAACCCGTGAAGAAGGCTAA
CGTGCGCCAGCAGCAGGTACTACTCCCTCTATGTAGCCCTAACCCTAGCAAATTGTACTATGGAG
TTTTATTTTTTTCTTCAAAATAAATAACTTGTAATAAAAAGAAAAAATAATTTCCATTTGCATAAAC
ATTAACAAATGTTCATCTTCTAAGATATTAGTATGATATCTATCAATAATTTGTTAAACATATAACTC
GTCAATGTTAATTACTACTCAAGTGTATGTGCTATAAACTCTTTCTGTCTGAAATTATTTAGTCATAT
TCATGTTATATATCGTGAATTTGATTGGAATGCAGGAAGGAGAGCACGATCAAGTTATGATGAAA
GAAGGGTTCTTAGCTCCTCAAGCCAATGTGAATGTGGGTGTAGGTCCGTACTAA

>*CqYAB3*

ATGTCATCAAGCTGTATGGATCAAGCTGCTGTTGCTCCTCCATCATCTGAGCAACTTTGCTACATA
CCTTGCAACTATTGCAATATTGTTCTTGCGGTTGTTATTTATTATTACTATAATCTCTTCATTTCTTAG
CTATATTTTCACTCATTAATTTTCTTTTATCTAGTCCTTTTTTTTAAAAAAAAAAATAAATATTATATTT
GAATTAGATTTGGGTTTTTGGAAGATTGTAAATTTTAATTTTATTTTTAAACTGTTGTACGGTATAA
ATATAGTCAAGTCTCAATCGTTTTATGAGACCATTTTGGTTCTCCCTTCTTTTGATTTCTTCTTCTTT
TGCAGTCAAAGAAAAGTCACTCTTTATTTAGAGGTCTTTTCTTCTACCCTAATCACCAAAATATTT
ACAAATTATAAATATTTTCTTTCGATTTTCAAATGTGCCCTAAAAAACCCGGTTTAAACATTCAAG
GATAAGACTGTATACATCTCGACAACTGTAAACTCGATTGTAATTATCATATTGTCATTAGTGTTTT
TCAAGGGAGTTTAATTTTATTTTTTCAAAAAAAAAAAAAAAGTAATGAATTGTATTTAGTTGAATTG
TTAATTTATTTAGTTTTTGTTTTTTTGATGTTGATGATGATGGATCATGTGTTTACATTAAACAGGTG

AGTGTTCCATGCAACAACTTGTTCGATATAGTAACCGTCCGTTGCGGGCACTGCACTAATCTATG
GTCAGTTAACATGGCCGCCGCCTTCCATTCGCTCTCCGCATCCTGGCAGCAACACCAGCAACAA
AACTTTCATCAAGTATTACATACTCTGTCTCTGTCTCTAAAAGTTGATCGGTCTCGTTTTAATTTTTT
TTAATCACATAACCATTCTAAACATAATTTTGTTATTAATCTACTTATATACCTAGAGTTTTCAAATT
AATAGTGTACTCAGTAATAATAACACATTTCTCAAGCTTGTCTTAATTTTTTTTTTTTTTTTTTTAAA
TATAAAATTATTCTACTGTATATGTATATTTTGGGAAGTGGGTGGATTATTAGATAATTTTATTAAAA
ATAAAAAAAGATAAATAATTTGATTTGGGAAGTCTAGGTCTCTTGGGATTTGTTTTTATAGGTATG
ACCTTTTTGTTGATTTGATTGGTAAAATTTGTTAGATCCTGCAGGTGTTTTTTTTTTTTTTTTCCTTCA
ATTGTAGGCTATAGCACACTCTACATATATATAGGTCTATTTATTTTAATTAATACTCCTTATTTACCT
AAGCTAGTCTAGCTCTATGTCTTATAAACAGTATGCTAAAATCAAATAACAAAAAAAGTTAAAAA
TTAATTACATAAACCTTAATTTATAGGGGTGTTTAAGGCTTTAAGCTTTGCTTAGTTACCCATTTCT
CTTTAACTTTCTTGATCTTTCCTAATCATTTTTCTTCTTTTAGCTACCTTGTTTGATTTGTGTCTCTCC
TCGTCTTAAACTTCGATGATGACTAGTATGCTCGATAATTACTTGAGCTGGTTGAGCCCTATTTTGT
TTTTTGTTTTTTTTTTTTTTATAAAGAAATTGTTGCGAACAATGTTTTTTATGATTATACAAAGAGTG
TTTACACTTGTCGATTCCTAAACTCTACTACGCCGACATCGATTATCAATTAACATCTTATGTTAAA
CATATACTAGTCAAATAGTTAAGCATATATTAAACATTGATGATTAGTCTATTATGTTGTTAATGTTT
TTTAAATTTATCATAACATTTAATTAATTTCTTTCATTAAACTTCATTCACATATGTTTTAGGCACCA
AACAATGGCAATATGGGTGAATATAGGATTGACAATTTGGGTTCATCCTCCAAGTGCAACTACAC
TAACAAAGCAGCTACAACTATGCGAATTTCGCCTCCTATTAGCAATAATTCTGCTGAGGAAAGGA
TTATTAATCGCCGTAAGATTAATTTTCTTCTATTTAATTCAACATTAATACAACTATTAGTTTCTTTTC
TAGGTATCTAATTAGTTTTACAATCTTATGTTTGATGATTCAAATCATTAACTTTGATTACAACGTTT
TTGTTCATGTATTTAGCACCTGAGAAGAGGCAACGCGTACCATCTGTCTACAACCAATTCATAAA
GTAATTAATCGTACACTTAAGATCGAGTTAATATATCAATATATGTACTTGTATATGATAAATACAA
TGCATTTAATTCAGTAATTAATGAATTTGTTATTTGTGAAATTTATGCAGAGAAGAAATTCAGAGG
ATCAAGGCTAATAATCCTGATATTAGTCATAGGGAAGCATTCAGTACTGCTGCCAAAAATGTGAG
TCCACCAACATTGTCAATTTATCTGCACCATTATTCAGTGTCGGCCCAACTTTGAACATTAATATCT
CTTGTAACATATACAATAATTAAATAAAACGATACTTTTATAGGTCAAAGTTCGATCAAGAAAGTA
TATATGACAGTGTGAGAAATAAATATACTTTGAATAATTATAACTTAATTTGCTTAATTATTTTTTTT
TTGTTAATAACAGTGGGCACATTTTCCTCACATTCATTTTGGGCTGATGCTGGAGACCAACAATCA
ACCTAAGCTAGATGAGGTAAATTTTCACAGCTATTTACTTATTTAGTCCTGCATTTTTCGTTAAATT
TAATTTACTAAGAACATTATACACGTACTAATTTTAACCAACACCATAATCATATACGGAGTAGTA
TTATAATTTTAATTGAAACAACCTTAATAATATATTTTAAGCATAGTGCAACATAGAACCCTTAATT
TTTACTTAAACGGAGTATAATTTATCTTAGACAATGTAAAATGCAATATTGGTAAAGGAAGAATG
GGAGATGAATTTGAACATACAAAAATAAAAATTAGATAATGTATAAAGCAACTTAAATTATGTAA
TAGAAAAATGTTCTAGGAGCATGTCATTCATTTATTGTCAAAAAATGTTTAGTTAAGCTGAAAATT
AACAACAAAAACATGGATCATATATTGTCAATTGTTTAGTTAAGTTGCATGATAATTTTGCTTTTA
ATTTGATCTTCATTTAATACGTGTATAACTCTTCTTTTGTTGAATGATGGCGGCATTTATAATTAGTT
TGATTAAGTTTCTTATTATTCAGTGAAATCCTTCAATTCACTAAAAGTCTTGTGGTTGATCGCGATT
TTAGGGCTCGCAAAAGCATCTCATGCCAAGGACTGCTTTACTAAACAATTGA

>*CqYAB4*

ATGGCAACACTTAATCGTTTATTTGATACTCAAGAACAAATTTGCTACGTCCAATGCAGTTTTTGT
ACCACCATCTTACTGGTAATCTTATGTTTTAATAATGTTACTATGTACCAATCTTTTTGTATACATGC
GTGATTAATTACTCCCTCTATCTATCCTGAATAAAGATATTGTGACGTCTGTTGATAGAATAATTTG

AACTACCAATGTAACAATGTACTCAACCTGATTATCAGGTCCGGATTAGTTTGTATATCTGTGTTTT
AATTATTATTTTTTTCTTTTTTATCGTTTTGTGAAGGTGAGTGTGCCATATAGTAGTATGACAATGGT
GGTGACAGTGAGATGTGGTCATTGCACTGGTCTTCTCTCAGTCAGCATGTTGAAAGCCTCCTTTG
TTCCCCTCCATCTTTTTTCTGCCCTTAACCAAGATCAGGTAATACTAATTAAGTTTACATGATTAAT
TCTTCCCATATAGTTATCTTAATAACCACATTTTTAATGTTTTTACCTTTTTATTTACCATTCTTATATA
ATTTAATCTACTTTTATTTCCTTTTGTCATAAATAACAATGGAAATTTGTCCTTCCTAAAAGGAATC
ATAAAATGTACATCATTTAATAATTATTAGCAATATATATTATGAAATACAATACTACTGAATTAAC
CTGATATCTTGGATATTTTGTTATTAAGGAACCTCAACTATATTTGTACACAATAAAATTATGAATA
ATTGTAATCTAAAGTTTATTTTGGACAATTTAACCTAGCTTAGTGAACTTAACTATTAATTTAGAAA
TGCTCTTGTTTTATTTATATATAGAAAAAGTTTATACATATCATGATTTAATTTTACATAGGTGAATT
TTAAGCATCAAGAGGAAGTGGATACATCAAAGGCGCATATGGACAGGCATAGTACTACTTTGTC
CATGTTACCCTCTTCAGAAGAAGATAATGACGATGAGGAGGAGGATGAAGAAGAGGATGAAGA
TAACATTGCGCTTGAACAAATTGTTAATAAACGTATACACTTCAAATTTGATGTTCATGTATATGTT
ATTGAATTTTTTCGCTTTAGTATCATGACCTAACATGTTCATAATATACAAATATTGTTGTTGTTTTA
ATTAAATGGCAGCTCCAGAGAAGAAAAGAAGGGCACCTTCAGCTTATAACAAATTCATCAAGTA
CGTACGTAAATTTGACATTATTATTTATCCATAATTTTTCATTCAAGCGTGTAGTAATTAATAATATA
CAATGTATAGAAAAATAAATAAATAAATAAATATATAATAAAACCTGTTTCGGTTGAAAACAATG
GGACCTTGCCGAAGGTCCACTAAAATGGAGAAGACTGGATTCGATCCAAGCTGTGTTGGGCCGA
AGGTTTCCTGCACAGTGAACACACGTAAACTAGCCTCGGGGGTGTTTCCGAGGAAGGCCCCTCC
GATGCTAAAGTCAGTACAATGTGTAAAAAGGAAGACACTATGCAAGAGAGTTTGTAGCTTGAGG
CTTTTGTGGAGTATTTCTCAATGCAAGAATGAGCATATGAGTTGGAATAATCCGTGTATGAGATAA
TGTATGTGATGATGCATGTGTTGTTGTGTCTCGTATGTGATGTTATGAGATGTCTTATGTAAGAATA
TGACTCTCTCCTTGAATGTGTTAATATGAGGGCTTTTATACCTCATCATATTTGGAGAGGTTGGGCT
TTAGCCCAATTGGCTTACTTTGTGGGCTGACTTTTGACTTTTGAGCAATTGGAGAGTTGAACCTTG
ACCTTGGGCTTTTAATTGGATCATTTAGTGCTCAGGCCCAATTTTGACTAAATTGGACCCAAACA
ACTTGCCCCCCAGACCCGATTCGGAAGAATGACGGGTCGGGTTTGATTTAACTCAGAAAAGGAG
GGAAGTAAATGATTTAATTGATCCAAAAATTTGAATTTAAACCGTAATTAATGAGATAACAACGG
TTAATGATTTGCGGAAGTTGGACGGATAATGGGCGGTTTAATCACAAGACATGTGTTTAATGGTT
ACTTAGTGGGTACTTAAGAGAAATTAAAGCACAGACTCTTCGTATCCCGTACCTGGAGATATAAA
CAGAGGCGCGTCATTCTCCTCACTGATTCACCAACACAAACCCAGAATTCTTCGAGAGAGAATC
CAAGAATCCGTATCAAAACTTCGCAAATTCCCCCGAGCATTGAGCTTAGAAGCTGAGACTTGCC
ACTACGAACTCGTAAATCGGCAACTTTCGCTTCAGATTAGGTAAATTCCTTTGATTTCAGTTTATTT
TTCTTCGAATTCGAACTCCTTCCCCTCTTCTTTCGACTTTTCTCTTCGAAATAGTCCATGGCAAGA
GGAGTTAGAGGTAGAGCCCGTGCTCCAAGAAGAGCAAGGCCGATTCCCGATCCTAGGAATAGG
ATTGGGACCTGGAATGGTCGAAGGCCGGAGTCTGACCCTGCTGATGGGATTAGGGCGACTCCCC
TTTCCTCGGCTCCTCCTCAAAGAACGGATGAGCCGAGTGCTTCTGGACGGGACGATGAAATTCC
CGCTGTCCTTTGGGATATCCTCCCTGGGTTTTCAGAGTGGGAGAATGCCGAAGTCGCTTCGGCCA
TGATAAAGAACCGCCCCTACTCATGGTGGGTAGAGAACGCCGATCAATATCGATTTGCCGAGCA
AATTGAGTTGCACTACGGCATTTTTGAAGCTGATGGATATTGGTGCCGAATGCCCTCAGCATCCG
ATAGAATCCATCGTCCACCCCCGGGGTATGTGGGGGTGTACACGAGACAGCTTCAGTATGGGCT
GAGGTTCCCTTTGGACGTTTTCGTCCGAGACTTGCTGATCCATTACAACATTTCCCTATGTCAGCT
AACCCCCACCAGTATCCGAAGGGTGATGACATACTTATGGGTCTGCAAGTTCTTCAGATACGTGC
CGAACCTGCACTGCTTCAGAAAGCTCCATCGGCTAGTGCCAAATATTCAGAGTGGGCACGGAAG
CCATGGTTCTGGGTGGTATCGGATTGAGTCCCGGCCCCAATACTTGACCATGTGGCCAAATGACT

CTTCAGACAAGGACTGGAGAGGTCAATGGATTTGGGTTAGGGCACCCATAGATCCGGGCCACCC
TTGTGTCTTCAGCGCTCCCCGGTACTTAGCTGAGCCTGATCCCGAAATGGGAAGTCTAGGTCCCC
CCGCAGATAACGGGGCGGATCCGAACCTGGATCAGCAAAATATCTTCGGCAAGCTGCCGAACC
CGGGGGGGAGTGGTCGAATGCAGCATATCCCGGAGACTTGGCTCCCCGATACGCATTACGTTTTC
CAAGAGAATATGCTAGCAGCTGTTGGCCTCAGCCGAAAGTTTAAGGATGGTAAGCTTCTTCGGCT
GGGGCCGAGGCTTTTAACTACCTCTTTTTCTTCTCTCGCTTTGCTTTTTTATGCTGTGCCAACATAT
CTGAGCTAAACTTTTTCCCTTTCCTTTTTTCAGAGCGGGACGAGACCAACGTTGGTATTAACTACG
CTCGTCTTGGTGTTAACCGAGAGCGAGTTGTAACTGAACGGGCCCCGTCTATCACTGAGCCGAG
GCATAGGGAAGTTCATCTGGACTCTTCCCACTTCCAAATTGAGGCTGTTGCTCTGTATCAGAGGC
AGAAGCCTTGGTGGAAGAAGAGAGACCCTTGGTTTCAGCTGCTGAACGAGGAAGCTGAAGCTG
CCCGCACAGCCGAAGAGGTTGCACGAGTACTTGAGGCTTGTGAAGGAGGAGAAGGGTCCTCCA
GAGCTGCTGAAGCCGAAGCTGCAGCTAGAGCAGCTGAAGAAGACGAGGCTTTGCTGAAAACCT
CGGTGAGCTTTCAGACAACTTTTTTCTTTTCTTCTTTTTTTTTTTTTTTTTTTTTTTTTTTTTTTTTTT
TTTTTTACTCACACTCTTCTTCCCCCCTGTCTGTTACAGTCTGAAGAGAGTAACAGACACAGAGA
AGGCAAAGCTCCTGTTCGTCCCGAAGATCAAATACAAGAGTCTTCAGACTCAGATATGGACGAG
CAGCCCCTTCTGAAAAGGCGCCGAAGGATGGCCATCATCGGCCCTCATGACTTTCAGCAGAGGC
CGATGACCATCGGCGTCGCAATAAGGGAACCTGAGGAAGGCAATGGATCGCCGAGGGTTCCCC
AGCCAACACCCCAATCTCCTCAAAAGACCCCTCCGCCACCTCCTGAGATGGAGGTGGATCAGA
GAACTCCCGAACCTAAGGGAGGGGTCCAAGAAGAAATCAAGGAGAGTGCTGCTGAGCACGAA
AATAAAGATGCCGTGATGTCAAGTGCTGAAGGCTTCACGACAGATATCGCAAGAGAGGTTGAG
GCTTTCGTCCTCACCCCTGAAGAAGAGGAGGTCATAGTACTTGACTCCTCTGAGAAGGACGATG
CTGAGACAACCAAAGCTAATCAAGCTCCATCGTCGTCCTTCCCTTCTTTCCAGACTCTGCATTCT
GGAGAAATGGGTTTCTCGGCCCCCCGAATTCCAATCCCAGATCACATCTGGGACTTCATATGGGG
TCAAAACCGAGAAGGAGCTGCAGCCTACTTCGGCAATTTTGCTGGCATGTCCGAAGTAGACAGG
GCAAGGATCTGGTCGCTGAAGGCCACTGCTGGGATGGGCGAATCCAGTAGATCAGTTCCTGACC
CAGCCAGGTCTAACCAACTGCTGATGATGCAGGTAATTTTCCTTCATTTCCTCCGAAATTCAGCTC
TATTTTTTTTTCTTATTACTTATTTTTTTTGTCTTCCTGTTTTCAGTTCGTGCACAATCAGACTCTCAC
TGTAGAGCTGATTGATACCCTACAAGATAGGGTAGACGCTGCTGTGCACGAAAGGAAGGTGGCT
GAAGAGGAAGTTAAGCTTGCTAAGCTGAAGCAAGCAGCAGCTGAAGAGAAGCAGCAGGAGGT
TGAGGAGTACGGTAAAAAAGCTGCCGAAGCTCTGAAAACCTTCTGCGATGCTATGGAGATGGA
GGTTTTTCCCCCACTGATGGAAGGGGAAAAATTCCTCCAGGAGCATATGGGAGTGGACGTCTCC
AAATTCAAGGCTGCTATTGAGAGCCGAATTCAAGAAGGAACTGCTGCACAAGCTGAAGTGCAG
CAGCGTCTAGAGGCTCTTGAAGCCAGCCGAATGGCTGCTGAAGAGTTGCTCTTGGCCAAAGACG
TCCAACTCTCAGAGGCTGCTGCTGCTGCTGAAGCTCAAAGAGCTAAATTCCAAGAGGCTGAGG
ATCAACTGCTGATCCAGAAAGTTGAAGTGGCCCAAGCTCAGACAAAGGCTTTCCAAATTCAGCA
AGAGCTGAATGAGCTGAAGGGGTCCTCCTCCAAAGTCGCTGAACAGCTGAAGAAATCCAAGGA
AGACTTGGAGGAGACCCAGAAGAAGCTGGCTGATGCTGAGGCTTTACTCAAAGTCCGTGTTTAC
ACCCAAGAGGAATATGAGATGGGTTTCAGAAACGGATTCCGAGTATGCCGAAGACTCTGCCTTC
ATGCTGAGCCGAAGCTGGACTGGTCTAAATGTGCTGAGTGGGTCCAGAATCCCGAAGACCCTCA
CATGAAGTATGCAACTCCAGCTGAAGCTGAAATTCTTAAGGCTGAGGAAGCCGAAGAGGAAGC
TGAAAGACTGGAGTTGGAAGCTGAGCAGAGGGAAAGGGAGGCTCGGCAAAGTGCAAAGCCCG
CCTCTTCTGCTGCTGGGGCTGAGGCCACCATGGGACTCAACGCTGATCAGTCTGATCCTCCGGCT
GAAGCATAGGAACTCGGGCAGTCGTCTTCAGAATCTTGCGGTTCATCATGTGCTTAACTTTAGAC
TTGCCGCTTTCTGAGTTTTTTGCCTTGTTTTTTGCTTCAGCTTGCCTTGTCGTACAGCAGTTTTATTT

TTTTGCTTATGTACGTATTTCGGCAGCTGTTATTTTCCGCTTGTGGGCGGCTGCCGATACTGAATTA
TTGAACTTGTAGCTCAAAAATTTTTTCTTCAGTTGCTTTATTTTTGAATGCTTTACTGTTTCTTTTCT
TCAACTCTGCCGAACATAGCTGCACTGCGTGCTTCTTGCCCCCACAGAAATTGAAGTATGACTTT
AGTTGAATAAGCAATTTCTGAACTTCTTCAAAATAGATATCCGAAACACAGGGCCAACTTAAGG
CAATGCTTCTGGGATACAAGTCTTACTGAAGATTGACTGTCTGAAAGTTGGATATCCGAAACACA
GGGCCAACTGAAGGCAATGCTTCTGGGATACAAGTCTTACTGAAGATTGTCTGAAAGTTGGATAT
CCGAAACACAGGGCCAACTGAAGGCAATGCTTGGGATATTAGCTTTCTGGGACTAACCTCTTTA
GAACAAGTCTTACTGAAATTTGACTTCAGCTGAAAGACCAGCGTTCTTAAAGAAGACGTATATC
CGAAACACAGGGCCAACTTAAGGCAATGTTTCGGATAATGGCTCTCTGGGATACAAGGCTCAGT
TAAGGCAATATCCCAGGATTGTCCATGGACTCATGTCTTTGGAGAACAGGTCTTACTGATGTTTG
ATTTCAGCTGAAAGACCAGTGTTCTTTAAAAAGACTGAGTTTTGTAAGCCATCTGAAACCCAGTT
ACCCGAAGGCGAAGGTTTCAGACGAGGCTGATTTAAAAAGGCTGTTATCCTTGATTTTCTGTCTA
TCTTTGAAAAATGGATACCCGAAACACAGGGCCAACTTAAGGCAATGTTTCGGATAATGGCTCT
CTGGGATACAAGGCTCAGTTAAGGCAATATCCCAGAATTGTCCATGGACTCATGTCTTTGGAGAA
CAAGTCTTACTGAAGTTTGACTTCAGCTGAAAGACCAGTGTTCTTTAAAAAGACTGAGTTTTGTA
AGCCATCTGAAACCCAGTTACCCGAAGGCGAAGGTTTCAGACGAGGCTGATTTAAAAATTTTCA
ACTGTTGCTGTTATCCTTGATTTTCTGGCTATCTTTGAAAAATGGATACCCGAAACACAGGGCCA
ACTTAAGGCAATGTTTCGGATAATGGCTCTCTGGGATACAAGGCTCAGTTAAGGCAATATCCCAG
AATTGTCCATGGACTCATGTCTTTGGAGAACAAGTCTTACTGAAGTTTGACTTCAGCTGAAAGAC
CAGTGTTCTTTAAAAAGACAGGTTTTGTCAGCAATCTGAAACCCAGTTACCCGAAGGCGAAGGC
TTCAGACGAGGCTGAATAAAAAAACCCTCAAAGAGTAGATAGAACAAAGAAAATTTTGAAAGGA
TAAACAGTTGTAAATTTTATATAAATGGCGGCTCCAAGGCCTGTCATTAGTTCAAAATGTACTTAC
AAAGATTGTTCTGCCCCGAAGGGTTTACACATGGTATTTTTTCAGTACATCAGCATTCCAGTGATT
CTTCAACTCAGTTCCATCCAACTGCCGAAGTCGGTATGTCCCTGGCTGAAGCTCATCATAAATTT
CATAAGGTCCCTCCCATGTAGCTGAGAGCTTCCCATGGATTCGGCCTTTCTGAACTGCAGCAGCA
TTCCGAAGGACAAGATCCCCAATTTTGAGAGGCCTTGCATGGACCCTTCAGTTATGGGAGATGA
GGTCTGAACTTATCCCGGGCATATCAGCTGCCGAGAAAGTAAACACATCTCTGTTATCCCGCAGG
AGCTGAATAAGTTCACAACGGGTGTTAGGATCAAGGTCCATCCCGATTGTTACACATTTCTCAGC
ATCTCCTTCGGCTAACTCAACTGTTTCAGTCTGTTGGTCCATGTCCATTGCCGATGGACTGGACTC
TTCAGAATCTGGTCTGCTGCCAATGTCAGCAGGTGTCCCAGTCTTTGTGTTCTTCGGCTGGCTCTC
TGAGTCTTTCGGCTCAGATGAAGAGTCAACCTTTTCCCTTTCGGGACCTTTTTCCCTGGGCACTG
ATCCTTGGGCCGAAGCACTTAGGCCTTTCGGCTTTTTTGTGCTTGGCTCTCTTTCTTTTTCTTTCTCC
TCCTCATCCTTGTCGTCATCTGGGCGACTACTGGATGGCTTGAGGTAGTTGCACCTTCGGGCGTCC
TTTTGGTTGCCCTGTATCCTTTCGGGGAAGCCGGCGTCTGAGACGTAAATCATCAGCTGATGATA
AGTTGAGACGACCCCTTGGATCTCGTGAATTATGGGTCTCCCCAAAATCACGTTGTACACTGAAG
GAGCGTCGATGACCAAAAACTCAATCATGACGTTCCGGGCTGCCTCATCGCAACCGATGGTGAC
AGGAAGTGTCACCTTCCCATCTGGATAAGTTGAAGCCCCATTGAACCCGGATACCGGATAGGGT
ACCGGCGTAAGGTGCTTGGGCTTCAGCTTAATTTGGTTGTAGGCCTTTCGGAACAGAATGTTTGC
TCCGCTGCCACCGTCAACAAGGACCTTACTGACGTCGTGGTTGGCCACCGTGGCGATGATCACC
AACGGATCCTCGTGAGGGAACATAATCCCCCGGCAGTCGGCGGCTGTGAACGTCATGTTCGGCA
CCGGCGACTGGGGAGATTGAATGTCAGCGTTGTTCACCTGGTGAGTCAAAGCTCTAAGGTGACG
CTTGTGCTCCCTCCGAGAGTATATTCCTCCCGAGATAACATTGATCGGAGGGAATTTCCCCTCCGG
TTTTTTACTGTTTTTCCGGGGGAAATCAAGGTCCTGCCGAGAGTCCTTTGCTAACCTCTCGGATTC
TTTTTTCTCGCGGTGATCCGCGATGTATTTTTTCAAGTATCCCTTCCGGACCATGTCTTCTATCTGGT

CCTTCAGCTGGATACACTCCTCAGTGTTGTGGCCCGAAGACTCGTGGAAGTCACACCACTGATTT
TTATTTTTTTGCTGATACCACTCTGGGAGTGGTCTCGGCTTTCGGTAACTCTGCTCCTCCTTCGTCA
CACTGTAAATTTTGGCGCGTGGAAGAGAGAGCGGAGTGTAGGACTCGTATCTGCCTGTGTATGA
GGCAAACCTGTCCTTCTTTGGGTCGCTGGCCGGAGATGGCTCCGGAGCCTTTGATTTCCCTTTGC
CTTATGGCCTTGGCCTTCAGACAAGGGTATTTGCCTCTGTTTCCCTCCCCCGGATTGAGGTTGGGT
TTTTGCTTTATTCCATTCTTCGGCTCGGATGAACTTATCCGAAGCACTAAGAGCTTCCTCCAAGGT
CTGCGGGGTATGCATGACCATGAAGTCTAGGAATTTTCCTGGCTGAAGGCCATTCTGCATTGCAA
AAACAGCTAGCCCCTGGTCCAGCTGAGAGACGCTTGATGACTCGTTACTGAAACGGGTCAGGTA
GTCTCGGAGGCTTTCGGTTTCCCCCTGTTGCAGACCCATGAGCTCTGCGGTAGTCTTTTCTCGGCG
GTTTGCCGTGGAAAACTTGCTCCTGAACTTGGAGGACAGATCATCCCAACCTGTTATGGAGCCTT
GCCTTACTCCCTTGGAGATCCACGCTTGCGCGACCCCCTTGAGAGTGGTTGGGAAATATTTGCAC
CAAGTTGCCTCGGATCTGGGGCAAAGATGCATATGAGCCTCATACGAGGCCACGTGGTCCTTCG
GATCCGTGGTCCCGTCATATGTGATATGCGACGGTATTTTAACCGTGGGTACGCGCTCTTCTAGTA
TCCATTGCTCGAATGGTGAACTAGCGGGAAGCATAATGGCCTTGCCCTTGTTCCTGGCCGAAGGA
ACCGCGCTCCGCCCTCTCAGAGAGGTAGGAGTGGTTTTCGTCCCTGGCTGGGAAGTCTGGGCCT
CGGCTGCAGGGAACGTGAGGCGACTACGCACGCTCGGTTCCTGGCCGCCGAGTCGGTCAAGGA
CGCTTCTGCGAGCAGTCTCAGGTTCCCGGCGAGTAGATTCTGGCCGAGAAGCTGAGCTGCTGCT
CTTATGCGCTGCTGGTCTTGAGGATGAGGATCCTTTAAATAATTGAGAGTTCTTCTTCCTCTGTTTT
TCATTTTTCCAAGAATCCTCCTCCCACTCTTCCAGTTCCCGGAAGGAAGGAAGCTTCCCACCGTC
ATGGTCTAACTTCCTCCGCTGATGCCGGGTATGCGGAGGAGTTCGATCACGAGGCGGTGGCATAT
TCTGTGCCCTCATTGCCTCCATGAGTACTGCCAGCGCTCTTGCCACCTCTTCCGAACTGGGCCCC
GTCAAAGGGGTCTCCGGCCGAGTGTGGGCATGAGGCCGTGCAGCCGATGGTTGGCTTCGGGAC
ACTGAATATGTCCTATGATCAGACTGACCCCTGCTAGACCGAGAGTGGTCCGGCCTATATTGAGA
GTCATGCATTATAGGAGATAGGGAATCCTCCTTGTGTGATCCTCCCCCCGAGCCGGCACCCTTGG
GAGAGTTCCGGTCCGGCGAGGGCGATGGTGTAGGGGTTTAGGCTTTTCGCCCGCTCCTTGACGG
AGGCTTTTGCTCTTGCGGTTCTTAGCCATGGCTGCTAAGACAATTGCAAGATGATCTTGGTTAGGA
ATTTGGCTTAAAGATTTTTGAAAGTCTTCCCCACAGACGGCGCCAAATTGTTTCGGTTGAAAACA
ATGGGACCTTGCCGAAGGTCCACTAAAATGGAGAAGACTGGATTCGATCCAAGCTGTGTTGGGC
CGAAGGTTTCCTGCACAGTGAACACACGTAAACTAGCCTCGGGGGTGTTTCCGAGGAAGGCCC
CTCCGATGCTAAAGTCAGTACAATGTGTAAAAAGGAAGACACTATGCAAGAGAGTTTGTAGCTT
GAGGCTTTTGTGGAGTATTTCTCAATGCAAGAATGAGCATATGAGTTGGAATAATCCGTGTATGA
GATAATGTATGTGATGATGCATGTGTTGTTGTGTCTCGTATGTGATGTTATGAGATGTCTTATGTAA
GAATATGACTCTCTCCTTGAATGTGTTAATATGAGGGCTTTTATACCTCATCATATTTGGAGAGGTT
GGGCTTTAGCCCAATTGGCTTACTTTGTGGGCTGACTTTTGACTTTTGAGCAATTGGAGAGTTGAA
CCTTGACCTTGGGCTTTTAATTGGATCATTTAGTGCTCAGGCCCAATTTTGACTAAATTGGACCCA
AACAAAACCTTAATAATTTACATGTGTATTTTGTATTATAGAGAAGAAATCAGGAGGTTGAAGGC
TAGGAATCCTAACATGACTCATAAGGAAGCCTTTAGCACTGCTGCTAAAAATGTAAGCTTAAATT
TGTCACCTTTTTTATTGGATTTAATGATAAAACGTACAAATTAAAATTTTAAATGTCTATAGTACTT
TTATTTGATTTTTTTTTTCTTTGTTAATAATTTTGCAGTGGGCTCATTTCCCGTCGGTCCAACATGAA
GTTGATGAAGATAATGATAGCTATGAGAACAGCATGACACAAAACTTAGACGACGAAGATGAA
GCAAATTGA

>*CqYAB5*

ATGAACACCAACACGATGGAAGACAAAGTGGGCTCGGAGTTGGCTCCACCACCTCCGGAACAT

CTTTGCTATGTTCGGTGCAACTTTTGCAACACTGTCCTCGCGGTATATATATATATATATGACCAGA
GGCGGGGCTATAAATGCTAACACTGAAAGTGGCCCGGTGCAATTTTGTTGAATTATGACCTAAAA
TCAATTTTTTAAGGAAAAGTATGTTACTAAATTAAAAAGTCGGGCCCTTTTATAGTAGTTTCACTT
AGGGGCCCTGTGCGGTCGACCACCCTGCACATGCTCAGAACCACCCCTGTATATGACTAACCCT
CACCATTCATATTTTCTACGTCGTTTCATTTAGCTTAGTATTTAATTAACTACCTAGCTACTTAATGA
TTATTATCATGTATGAAAAGAATATGTATGTTTTAAACGAGTCAAAGACTTTCTCATGTTATTAGTT
AGACAATAGTACCATATATAATTGATGCTTACTTTGTCGGACATGAATTTTATCACATTGACTGTAC
AAGTAACGTGACAACTAAAGTTGGACGGAAGATGCTTTGTTACATATACATTATTTAACTAAAGT
GTTATTATAATTTTCGAGTGTAGGTTGTGATTCCATGCAAGAGGTTGTTGGACACGATAACAGTGA
AATGTGGGCATTGTAGTAATGTATCTTTTCTGAGCACTAGGCCTCCTCTCCAAGGGCAATGTCTTG
ACCACCAAATCACCCTTCAGGTAATAATTTTTTTTTTTTTTTTTCACCCTTAATGTTAATCAATCTA
TCTAGATAATTACTTTGTAGTTCAGTTATAATTTATATATCTTTGTTCATGATTTATGAAAATTGACA
TACAAACTTTGATTTGTAAACTAATATTAACTCCAAGTTGAAATTAATTGCACAATTAGCCTAAGA
TGCATGCATCATGCATGTATAAATATTGTTATATTGATGGAAATAAAATTATTGTACGTGTTGTTCG
TTGTTTGTGCTAGGGGTTCAATTTCTTGGAGAAACCAGGGGGTTTTTGCAGCACCATTGATCATC
ACAGCAACAAGAAAAGTGAACCATCTCCTTCGTCGTCATCCACCTTAACCGAGCCTGTTTCTCC
AAGGCCATTTGTTTGTAAACGTATGTTTTTATTTCCCAATCTATTTACATTTATTTATTCAACTTTTTA
TATAGCCTAATTGTGTGTTTTATATAGCTTATTTGTCGGTTAAGGGTTACTTGGTATGTTTTAGCTTG
GCCACACAGTGTGGTCAGTCGCACACAAAAGTTACTTGCTTAGAAATTGATTCTTTTAGTTTTTAC
TAGAATGAAAATTATCTTTAAGATTTTATAAGCTCTTTTGATCATTAGCCAGAAGATTTTGTTTAGA
TCTACGTCTTTTATACTAATTGAAATTATTACTTTTAATTTGTCAATGAATGAGTTTATATTATTATCT
AATTAAGTTATTATAGCGTGATAACTGATTTATGGAATTGAATTTTTATTTATTGCAGCTCCTGAGA
AGAAGCATAGGCTTCCATCCGCTTATAATAGATTCATGAAGTAAGCATCAAAATTGTACACTAAA
ACTTCATTATCTGTAATAATTCATAATTCGTTCATGCATATCCGGAATTAAATGTCATATTGAGGTC
GAGATTTGATCTAGTAATGTGACAAATAAACCACGTACTTCTTCTGATTTGTTAGGGAGGAGATTC
AGCGCATCAAAGCAGCAAATCCTGAGATACCTCATAGAGAGGCTTTTAGCACAGCCGCAAAGA
ACGTAAGTTAACTACTTTTCCATCTCTTTTAACATTTTTAGGATGTTTTTGAATAATCTCATCCCAA
ATCCTTGAATCCAATTAAACATTTTTAAGATGTCAAAAAAAAAAAAAACATATATATATATATATA
TATATATATATATATATATATATATATATAGGACTATCTATCTAAGGGATTAAATACGACTTTTAACTA
CTGATTTTCTCCATTTCTTTGCTAAAATTAGATGATTATGAATTTCTTTAAGAAGTTCTTGTACGATA
CTAGATAAGAATTTCTATTTGCATGCTATAGACTATAGCATATAATTTAGCATTATTGTTATTATCAT
ACAAAGTTTATTTTAAAACTAGCTAGCAAGTAGCAAAGATTGTTATTCTGTACTTTTAATGTTCCT
AATTATATCAAAACTAGATGAAGCCCGTGCGATGCACGGTTTGTTTGATTTTTTTTTTTTTTTTTTT
TTTTTTTTTTTTTTTTTTTGTATTTATGAGCCTTCAATTTGTTATATATTTAGATAAAATTCCTAATATT
AAAAGATAATATATAATATTTAATTAATTTAAACTTAAAAATCTCATTCGCCAATTTTGCTTACATG
ACATTTGAATTATTTAATTCTAAAAGAAATTAATAATCTCCTATTTAATTGGCTACTACTAAGATTT
ATAATCTAACATGGCGCACTAAGAAAACTAGATCCTGTGGCTCTCTAAAAACTCGGGAAAAAAC
TCCCTTTTATATATATATATATATATATATATTAGATTATTATTTTAAAGAATTTATCCACTCTTTTTATA
TACTATATACTATGTACTGAGTACTAATTCAAAGTGTGAAGATTACTACGTAATGGATAGGTTTATG
AGTTTATAGAATTTATTTAATAAGACGGTCAAATTGTTTATGGAAGAAGCGTTTATTTTGTTGTTTC
TTGAACAATAACATGATAAATAAAAAAAAAAAATACCACCTCCATTACATATTATGTGCAACTTTG
AAATATTTTATATCTCACAAATAATACCTAAAAGTTGCATATAATTTGTAACAGAGGTAGTACTGT
AGTAGTACAATAATTATTACTTCGTAATTGTTTGTATGTGATAATGTTCAGTGGGCAAGGTTTCTTC
CGCACACTCCAGCTGGGTCACTTGCGGAGAGCAGCAACCCCAATTAA

>*CqYAB6*

ATGGAGTTCAGTACATCAGCAGAGCGAGTCTGCTATGTCCACTGCACCTTCTGCAAGACCATTTT
AGCGGTACTCTATTCTCTCTCTCCTCTTTATTTTTTTAATTTTATTTTATTTAAGGAAATAAATTACCT
ATATATTTATTCTTTATCTTTGAGTCAGATCAAAAGTTCAAAGCCATTAATGATTTTTTTTTTCCTGA
TCGAGTTTAGGTTTTGATAAACAAATTAAAATTTTGTTTTGATTAAAAAATTATGCCGTATATAACA
AAAAGAAACGGAGGAAGTATAATCTACTTTTAATTTTTCGAATTATGAGTTATTAAGCAGCCTTTG
TATAAAAGTAGGGCTGCACAGTTTAATTAATGAAGTATATATAAATTAGGGTTCATCTTCAGTGAA
ACAGGTAGCTAGTCTAGCTAAAAATTTATTATTAATTATACTTTGTACAATTTTCAGAGTATGTTTA
ATCCAATGAAATTTCATATGTACTTCAACATTATTACTGAGTATGATATCGCTTCAGAAACTTAATC
GATTTATATAAAATCTAGTACAACAAATAAAACACTTTTTTTAACAATTTTTTTTTTGGGTTTGGTA
TGATGATCACATCTGAGTGACTTATGATTCTTCATCTGTACCAAAGTATTGCATTGGTACAGTCTCT
TTTTTACTTGGCCTGTAAAAAATTGAGTCACTTTTTTTTTAAGGAAAAAAAAAAAAAAAATTCCCT
ACTTTGCTCAAGTTAATAACAAAAATTTTTAATTCTTCTATGATTTATTTCTTTGTATTAGGAGTATA
ATACAATATTAGTTAGTTTTTCTTTCTTTATATTAGAGGTCATACAAAATTTATTACTTCATAAAAGA
ATGATGCTTCGTATTTCATATGATCTGGACCGATCTTATAAGTTAAAATGAGTGAGATCATTTTCAA
TTTAACTTTTTTTTTTCCCTCTTGACTTTTATGTTGGGGAGAGAAAGGGGGTTGTCAGGGTTTATGA
ACCCAAGCCAGAAGTTGCAGCAAAAATAGAAAGAGATTTCTCATTTAGGGTTGTCTTTTTCATAT
TGTGTACCACACCTCATGTAACTTTATACTCTACGGAGTATATTCTTTTGATCGCGTTGTTATTTGTT
AACTACTCGTAAATCTTTTATTCTTAATACAAACATAGACAATCTAATACCAAAAATTCCGTTATC
ACTCTGTTCATACTATTAATTTTTGAGAGTACCAATCAAATTATTTTTCAAAACCTAATCTAAGTTA
TGAATTTAATTCATCAGTGAAGTGTATTTTAGCCTATTGATGTGTGGTGTGAGTGATAAATCGAAT
CTGAGTCCTTGTTTAATATCGATCTGTAGGGCTTTTATATATGAAATGTCAAATGTTCAAGATCTTC
TTCCACATGGGGTTAGGATTGGTTTTTGGTGGTACTATTATTGATTTTCTACTCCTTAGTGCAAGGG
TATTAAATTTGAATTAAAGTTAAAGGTTTCATAAACATAAAATTATACTTTATTAAGTTCAAGTGTG
AATATAAATCAATTCTCCTAATTGCTTTTTTCCTTTATAATTTGGTCCAATCTGTCGTAGCTAGGTTT
AACTTAATAATTCCTCAACAAATCTAGAGCATGAGATAGACCAGCACGTTGATAATGTAATTAAA
CTAATAACATGACAACTAATTCTAGATGGACATATATAGTAGTTACGATAATTTTCAATTCATATAC
TTCCTCCGTTTTTTTTTATCGCATCACTTTCTACTTTAGGAAGTTTACTTATTATTGCATCATTTCCTT
TTTAGAATAAAAAATATCTATGTTCACACTGCAAATACCCAACATACCCTTACTTTTTCACACTTA
CTTATTATTTTATCTTACATTACATGGACAATTTTGTATTTTTACGTTTCTCTCTCTTACTTTTTTCCAC
TACCAAATTTTGCCTCCAAAACCCTATGATGCAATAAAAAAAAACGGAGGAAGTATATTATCATA
TGATTTACATGATTCCTTAGGATTTTGCTGATTAGCTATTAATATATGGCAGGTAAGTGTACCATGC
TGCAGCATGTATAACATGGTAACAGTCAGATGTGGGCATTGTGCCAATCTTCTCTCTGTTAACATT
GGACTTTCACCTCCATCTATGCCTCATCAAGATAATTTCCAGGTATAATTAACAATTGATTTTGTTT
TCTTTCTTTCTTTACAAATAATTGAAGAAGATATTAAGAAAATCTATATATTACTATACGTATGTATA
TATAGTTGCTGAGGCAGCACTGTAATTATCAAGATGTGAGCAAGGATAGTAGTAGTACTATAACA
GGTGGCTCATCTTCTTCAACTATTACAACAATGGCTTCTGATGATCATGATGTTCAACAGACTCGT
CCCCCACCCATTCGTCGTAAGTACTTAATTTTTTCAGCTAACCTTAATTCGAATTATATATGAAATG
TTTAGATTTTCTGGTTTGTTAGTTGTTACTCTTCGTAGCGAATCATATATAGTAAGGTCTCTTTTCGT
TTTTGAGAATTTAAAGTCGTATAATGAATTTAAAATTATAGATACGTTTATATATGTATGTATGTTAC
GGAGTAATATAAAACCTGACAACCGAACTTCAATAGATCTTAATGACAACAAACAAGCCCTAAT
ACATAGGATTTTATGTTCATTATCATGCTCTCCTATAAATTCTTTTCCGGATTATGTAGTTTTGTAGA
ACCTTAGTTTCTGATAGAGTATAAAACATATTCCCGAGTCTCAACCATCAGCTTAAACTTTTGGTT

GAGTTGGTCCTATAACAGTTTCCTTAAGACTTTATCTAGGTTAATAAGCACATTCAATTTGAAGAA
GACTAGCAGCAAAGATTAATAGTTCAAAAGTTCAATATAATATTAAAGAAGCATTTGTTTTACTCC
TCAAAATTAAAGTCACTTCTTATATTGTTTAGACGACTAAACTAAGTTGTAAATTCTATTAGACAA
TGGATTAAAGAGGTGAGCCATTAGACAATGATATATAGTAAAGAGTAGATGATGATTACTACTTTT
AGTCGCGTTTTTATTATGTTTTGATTTCATGAAATTAACCTAATGAAGACTTGAACATCTAACTTAA
TTACATGTTCAACTTAAACATAGAAGCATTACTTCGATTTGATCTTATCACAAAACCAAATTCAAA
TGGTAACGCACAGCATTACATGCATACACACACACACAATAGCTTTAACCATGTTATTTGTTGCTT
TCTAAGGCATTTAAGGTAAACTCTAATTGTCTTATATGTGTATTTGGTGAGCAGCCCCAGAAAAG
AGGCAACGCGTTCCTTCGGCTTATAATAAATTTATCAAGTAAGGATTACTTAATCTCCATTGCAAG
TTCTAGTTTATTATGGTTATGAACATAGCTACAGTTAGATATGAAATGAACAATATTTTTTTCAGAA
TAGAGAAACTTGTAGTTATAATTGTTTGATTTTTTTCATGCTCGTTCTGAATTAAGCCATATATATCT
TGTTAAGAATGAAGCATTTGCTTTTGATTAAATACGTACTACTAATATGCTTTTGTAGAATTGTTCT
TAAACTGGGAATCTCACCCCCAATTTTGGTTTTTCTTTTTTATTTCTTTTCCCTTTCTTAATTTGGTTC
TCTATTGAAGGGAGGAAATCCAAAGGATAAAAGCCAGCAATCCTGAAATTAGCCATAGAGAGG
CCTTTAGTGCAGCAGCTAAGAATGTGAGTCTTTTTATGCTCCAATATTAATTTACATGTTATGCCAC
CTCATCTGATATGATTCCATCTAATCCGATCTAATCGTGTGTATCATATACGTATATATAGTGCAAAT
GATTATTTTATAAAATTCGTTGATTTTACTGCCTGCATGTCTATTACACAATTATACACCCGTGTGTA
CGTACACTGATGCTCATGTTAGTTTTACTAAGTACGAGTAAACCTCTTTTTTTTTTTGTTTTTTTTTT
TTTAAATAATGGTACCACGTACGAGTAAACTGTAGAAGCTAATAGTAAACAAAATGTATGTTGGC
TTGGCACGCAGTGGGCACATTTTCCTCATATTCACTTTGGCCTAAATCTGGATGGGCAAAGGCAA
GAAAGGTTGGACCAACCAGTTTCTGGAGAAGGAACAAACAAGTCTGATGGATTTTACTGA

>*CqYAB7*

ATGTCGTCCTCTTCCTCGTCAACTACGACAGGAAGTGGTGGTGGTTGCGGCGGAGGTGGTAGTTT
AGATAATAACTACCATCATCAAACTCCTAATTATAATAACAATGATAATGTAGAACATGTTATTTC
GCCGTCGGACCAACTTTGTTATGTTCAGTGCAATTGCTGTGATACTGTTCTTGCTGTAAGTATATAT
TATTCTAATTCTTATTATTTGTATAATTTTATCTATCAACTTGTAAAAAAAAATGATGTGGTGTAGGT
GAGTGTGCCGAGTAGCAGCTTGTTTAAGACGGTGACGGTGAGATGTGGACACTGTACTAGCTTG
TTGTCTGTCCATATGAGGGCTCATCTTTTGCCTGCCGTGCCTTCGCCTTCGCCTCCATCTCCGCTTC
CTCCGCCTCCGCCTCTTTCGCCGAATCACCATCATCTTTCTCCTCCCTCTTTCTTTACCTCTCCTCA
CAGTCTTCTGGTATGTTTTTTTAATTTTAGGGTTCTTCTTTGGTTAAAAGGAGACTACAAAATTAAA
CATAATATTGTACCTCTTTGGTTAGGATAAAGAAGGCCTTCATACTAGAGAACTTTGGACGAACA
ATGTGATAATTAAATCTAGACGGATGTTGTATAGGTTACCCGATGTTGTATCTCTCTTTGAAACTAA
GAGTGTGAACTAATAATGTGACAACTAAATTCCGATAAAAGTAGGCAAACTCTAACTTGTTAATA
CTTTATGTGGGTGGTGATGGGTTTTTGGGGAAGGAGTTGTAATAAGTAGTTGAATTGGTAACAAA
AGATGTGAATTGTAACCTAAAATCAATCAACGATTCTCTTATCTTCCTGGTCTTCTTCTGCATCATT
AATTGTGCTCGAAATTAAGGCTATTAGTAGACAGTATAATTAAATGTTTATGTGTTTGTGTAATGTG
TGTGTACTAGGAGGAGATTCGGAGCTCAGCACCAAATATACTTATCAACAATCATCATCATCAGC
CTATGTTCAATGACCCAATGATGTCCGTTCGAGGAGTTGATCATCTTCATCACCACCATCAAGAG
ATCCCTAAACCCCCACCCGTCAATCGCCGTAAGTCCCTTTTCATTTCCTTCTTCGTTAAGCAGGCT
TATGGGTAGTGTTGTAAGGTATAATAATTAAAAAAAGAGTGCTCGAATCGACCATTTATAAAACC
CATCCTATATTCCTATATAATCTAACTAGTTTTTGTTCTTAACTAACCCTGATTATTCTTCCAATTTTT
GTATTTACATAACAACGAACAATAATATCAAATCCGTAATCCCGATGAAATATTTGTATTTTCTAGT
AACACTTAAAAAAGACAACATTTTATTCTGCGGTTGTGCTTTTAGCCCTAAATAGACATGTCAAC

AGATTTTGGTTCACTTTACTTACAAAATTTGAGAAAAGAAGTGTAAAACAAAATAAAGTGTTGTT
TCTTTTTCTCTTCATTCATCATTCATCACTTTCAACTCACTCTTGTACTTGAACTCTTTTTCAATTGG
ACTTTAATTTGTATCATACACGTTTTCTCTGCACTCAAATTCTCCTCTATATTACAAGTACTTGTTAA
CTTATGTACTTTTTAACACTCTTAAATAATCAAAACTAACTACGGAGTAACTTATAGTATATTGTTT
TGTACATACTCCGTAATCATCTTTCTCTAGCCTTCTCAATATTGTGGAGGAAATATTAACCTCAAAC
ACGTAACTATTCGAAAAGACCTACCTAGTAATATCGTCATCTCAATTTCTTTTTAGTTGATAAGATT
CGTGTTAAGGTGTAATTATGTTTCAACCTAAATTATAATATTCCTTGGAGTAAATTCTAGCCAAATT
GAGCTACTTGCCATCACTTAGAAGAAAAAAAATAAATATAATTTCTTATTAGTAATTAGTTATTACG
AGTACCTTATAAAGTACAGTAGAATGATAGACTAGTGAAATTTTGATTGATTTAGGAGTAAACCG
AGTAAAAAAGGAGAAAGGCAATTATAAAGTGATGATTATTATTTCTAAGTTAGGTTGGCCAAGTG
TATGTCCCCCACAACCCCTTGAGCAGGGACATATAAAATCCAAGAAGAAGCGGTTGATGATGGG
ATAGATTTGATAGATACAATGCTAACAGAATGCTATGATGAGGTTATGATTTAGAATTGATAGAGA
AAGGGATTGGAATGAATGAATTTAATGCAAATTTATTTTTTTTAAAAATGAAAATGAAATTAATTT
AAGTTGTCAGAAGAAAATAGTGAGTTTAGTTAATGTGGTTTTGTTTGTCTTGTTTGTTTATATGTTT
GTTTTTTTTTTTTTTTTTTTTTTTTTTTGCATGTTTATACAATGAACAGCTCCAGAGAAGAGACAGA
GGGTACCATCTGCCTACAACAGATTTATCAAGTTAGTACCCTTTTTGTCGTACCTTATTTATTTCTT
AAATTTGATTATTGTTAGTACTTTATGTCCAATTTTGCAAATTGTAGATGAAATTAATTTAATGATC
ATGTGTCCGTCTAAAATATATAGTCACGTTACTATTTTAATCAAACTAGTTAAGTTAATAAAAAGTT
TATTAACATAACTTTATCAGGGTAAAAGAATAATTAACGCTAATTACTAATGAAAAGACATGCAA
TTGTTTTATAGGGACGAAATTCAACGAATCAAAGCCGGAAATCCTGATATTAGCCACAGGGAGG
CCTTCAGTGCAGCTGCCAAGAATGTAATAAAATCTTTGAAGTTTCTATGCTTGTTGGTTTTAACTT
TTAAATGACTAATTTTAGAATTTGAGTACTTTTCAAAAATTGCTCAAACATTATTGATTTTCTATTT
GTTTTGCGTGTAAAAAAGTGGGCCCACTTCCCACACATTCATTTCGGCCTTATGCCTGACCATCA
ACCCGTGAAGAAGGCTAACGTGCGCCAGCAGCAGGTACTACTCCCTCTATGTAGCCCTAACCCT
AGCAAATTGTACTACTTTTTTTTCTTCAAAATAAATAATTTGTTCTAAAAAGAAAAAAGAATTTCC
ATTTGCATAAACATTAACAAATGTTCATCTTCTAAGACGTTTGTTAAACAAACAATTCGCCAATTT
TAATTACTCAGTATATGTGCTATAAACTCTTTATGTCCAAAATTAAGTCATGTTATATATCGTGAATT
TGATTGGAATGCAGGAAGGAGAGCACGATCAAGTTATGATGAAAGAAGGGTTCTTAGCTCCTCA
AGCCAATGTGAATGTGGGTGTAGGTCCGTACTAA

>*CqYAB8*

ATGTCAAGCTCTAACACTGCTGCCTCTTCAACAACAACAAGCTTGTCATTGGACCACTTCTCACC
TTCTGAGCAACTCTGTTATCTCCAATGCAGTCGCTGTGAAACCGTCCTAGCGGTATTCTTTCTCGC
TCTTTTCATCATCTCCTTCTCACTACCACTTTATTATTTATTCACTCAATTTCTTCGTCACCTCAATAT
CTACATACTTGATATGATTCATACCGGCTTGTTGCTAGCGTTCACGGTCCGGTTCAATACTGGGTT
GTTGTTACACGACTTGTAGTGTGCTCAGGGTTTATATACATACTGGGTTGTTATTGTTGTAACCATC
CCTAATCTAGTTTCTACTTCTATGAATTTATTTTAGGGTTTTAATTTATTATTTTTTTGGGACAGGTAA
GTGTGCCATCAAGCAGCTTGTACAGGACGGTGACGGTCCGCTGTGGGCACTGCACCCATCTCCT
GCCGGCGAACACACGATCTTTACTACTTCAGCCACCGCCGGCTAGTCAGTATCACTTGCCTCATC
ATCATAACTACTACTCTCCCAACTCCCATTATCGTCTGGTATTACTACTATCTCTATGATATCAAATT
TATTAGCTAGGGTTTCTATTTTAGGAATTCAGAATAAAACTATCCTGATTGAAGGTATCATCTTGAT
GCAAGTTAAATGCTCATTTTAGAGCATAATAATAAGATTCTAATTCTGTATTGATTATAATTTTATTA
TTTTTTTTCCATTAAATTAGACCTTGTTAATGAATCATTTCATTTATAGGGGGAGATGCCAAATCAA
GCACCAAATTTCTCACTAACACAACCAAATGGTGCATCTAGCTACATGAATCCATCATCAAGCAG

AGGCGGTCCAAACGAGCTTCCGAGGGCTCCTACCACTAACAGACGTAAGTGAGCATATTATAAT
TCATCCCAAGTTGTAGTACTCCATGTTAATTAAGTTTTATTATTACTAATTTTAATGGGTTTATAGTT
TAAGGAAGAGAAAAGGGGGGTGGGGATGGATGTTCTTTTAATGCCTATATATGTCAACTTTATGC
AGCAACTTTGTCATTGATTCAGTGTTTGTTTCAGCACTGGATCAAGACTTTCTTACTCTCTACTTTG
GCATAGCTAGCTATAGGTGCCTATTAATCAATTTATTGAAATTTAAAGCAACAACGAACTAGTCTA
CTTTGGTCAATTTGATTTCTATGCATATAAGCTACATCAAATCAATTTTTTTGCCATTAAGTACATA
ACTTACATCCGCGGTTGCGGTCATATTTTTAATAATTTTTTTTTTTTTTTATATAAATGCCACGAAA
TATATACTAGTAGTCGCTGATAGGCAATACATACGAGTCACAATTTTTTTTGAAAGAGTATTGAAT
TTTTGCGAAAATTCAAAAGAAATCGCTTATTTTCTCTTAAATTGTACTCTGTAGTGAAATGCGTGA
AAAGTCTAGGCAAGGTGTTGATGTCTAATCTATTTTTGTTTACATAGATGAACTGTCTTTTTTTCAA
AATCGGAACTCTTAATTGATTGCTTCAATAAATGAATTGGTTAAGGCACATGTAAATTATTTGTTT
GTTCATATTGATCGGATCAAATGAAGAAGACATATGAAAGAACAAATAAACATGGATGTTTTTTA
GAGATAGAGGGGTTTGTTTGAGGTTCATAACAAATGAGTTAACAATGGACTTTACTGTAATTAAC
CATGGACAGTGCAGGTTATAAAGATAATACTGTTTTTGTCTTTATATGTCGATTAGTGAGTGAATG
AAAGAGATATTACTATCATTGTCATCCGGAAGTTTAGGGGAAAGTAATAATCATTTACTTGTACAG
TTGTATGTGTCTATATAATCATCATCATTGCATAAAATTAAAATAAAAAAGTTTGTTGTAGTGTTTA
TTTTTTAGGAAATTTATAGGAATATTGTAAAATTAAATTTTCTTGTGGTGTGAATACGGATTATATTG
AAAAATTATGCATGATTTGTTACTATTATTAACAACTTTTATACTCTAATCATGTTATGCATAATCTT
TAAAAAAAAAAAATCCATATGTTTTGAACTCTAGAAATTAGCTAGTTTTTTCTTTTTATTTTTTTGGT
TAACTATCTTGTCAAATTTAAGGTGTTTCTATTATTATTCATGTGTAAACTTATTAATTGAATATAAA
AAAAAAAAAAAAAAAAGTTTGTAAATATTATTTGCTAAAATTTGCATTTGGTGTGCCGCTGTATAA
CAGCTCCGGAAAAAAGACAGAGAGTGCCTTCAGCTTACAACCGATTCATCAAGTGAGTGATCAT
ATATATATATATATTCCCTTACATTCAATTATATATATGTATGTCTACAACAACCTTAAAATGAACTG
TATGTGCAGAGAGGAAATCCAGCGTATTAAGGCTGAAAATCCTGATATTTCTCATAGAGAGGCTT
TCAGTGCTGCTGCCAAGAATGTAAGTTTACTTATTATATTCACCTCTAATCATAGGAGTGGAGCAT
TATATTCATTTTTGCTGTAATTAGTGATCCCTAGTTTGATAGATCTTTAATTTTTTATGGTTATCTCAT
GCATGCGCTGATGCATGTTGTATACAAGTCATTCTGAGTCACCGGGACGATTTTGCTAATCTTACG
GGATCCTAATTCCTAACAATTTTTAATAGAGCTCTATAACGAAACTAATACAACCCCTCCTTAGCT
TCGCTAAATAGTAATTAAAGAATAAATGATGTAAATGAGATTTGATAACAAATCTTGATGCCAAC
CTGTGACTTAAATTTATGAATAAAAAATGCCGGATTACAACCGTCTACTTAATTCTATTATTATGTT
CCTATGTCAAATGTTAGGGTTTCATGTCAATTTGCAATGTAAAAGGATATTGCAATGAAAAAAAT
ATTCTAAATATGTTTAAATCAACAGTGGGCCCACTTTCCCCACATCCAATTTGGGTTGATGCCGGA
ACGACGGTGA

>*CqYAB9*

ATGATGAACACCAACATGATGGAAGACAAAGTGGGCTCGGAGTTGGCTCCACCACCTCCGGAA
CATCTTTGCTATGTTCGGTGCAACTTTTGCAACACTGTCCTCGCGGTATATAACTAACCCTCAGTC
CCTCACAATTCATATTTTCTACAACGTTTCTTTTAGCTTAGTAATTAACTACCTAGCTACTTGATTAT
TATCTAATGTGAAAAGAATATGTATGTTTTCAACGAGTCAAAGACTTTCTCATGTTATTAGTTAGA
CAATAGTACCATACATAATTGATGCTCACTTTGTCGGACATGAATTTTATCAAATTGTTTGTATGAG
TAACGTGACAACTAAAGTTGGACGGAGGAAGCTTCGTTACATATACACTTAACAAAAGTGTTATT
ATCATTTTCGAGTGTAGGTTGTGATTCCGTGCAAGAGGTTGTTGGACACGATAACAGTGAAATGT
GGGCATTGTAGTAATGTATCTTTTCTGAGCACCAGGCCTCCTCTCCAAGGGCAATGTCTTGACCA
CCAAATTACCCTTCAGGTAAGGATTTTTTTTCACCCTTAATCATTATCTCTATCTAGATCTTTACTTC

GAATTTTCGTTACAATTTACATTATCTCTGTTAATGATTTATGAAAATTGACATACACACTTTGATTT
GTAAAATAATACGAACTACAAGTTGAAAAAATTGCACAATAAGCCTAAGATGCATGCATCATGC
ATGTATGAATATTGTTATATTGATGGAAGTAAAATTATTGTACGTGTTGTATGTGCTAGGGGTTCAA
TTTCTTGGAGAAACCAGGGGGTTTTTGCAGCACCATTGATCATCACAGCAACAAGAAAAGCGAA
CCATCTCCTTCGATGTCATCCACCTTAACCGAGCCTGTTTCTCCAAGGCCATTTGTTTGTAAACGT
ATGCTCTTTTTTTTCCCATTATATTTAGGATGCTCAACATTTCATCGATTCGTATTTTCCCACATATAT
ATATATATAGCCTGATTATTAAGTGAATGATATTTAGAAATTGATCCTTTTAGTTTTTACTAGCTAGG
AAATTATCTTTAAGAAGTAGCTAGAAGAGTTTGTTTAGATCTACGTCTAATTATATTTTTAATTATT
GCTTTTTTCAAATGAGTTTTATCTAAGTTAAAACAGTGTGATTATTGATTTATGAAACTGAATTTTT
ATTGCAGCTCCTGAGAAGAAGCATAGGCTTCCATCCGCTTATAATAGATTCATGAAGTAAGCATC
AGAATTATTGTATACTAAAACTTCATTATTTTTAATAATTTATAATTCGTTCATGCATGTAGTCCTCC
ATCCGGAATTAAAATGTCATATTTAGGTCGAGATTTGAGCTAGTAATGTGACGAGCATCTCTGGA
CGAATAAAATGGTCGTAGTTTTAAGGAATTTAACAAACCGTAATTCTTCTGATATGTTAGGGAGG
AGATTCAGCGCATCAAAGCAGCAAATCCTGAGATACCTCATAGAGAGGCTTTTAGCACAGCCGC
AAAGAACGTAAGTTAGCTACTTTTGGATCTTTTTAACATTTTTGAGAATGTGTTTGAATAATCTTAT
CAAATCCTTAAATCTACCTACACACTCAAAATGTCAAAAAACTATTAAAAATACGTGTAATTTTTT
TTTTTTAAAAATAAATATATAATCGCATTACTAAACTAGATAAGTCCGATCCATATTTATTTTGCAA
TATAAGTTGTACTAATGCATGGGGCATTAAGAATGTAGCTAGAGAGCTATAATTTTGTTACTAAAC
TAGATGCTTAAGGGAATATCTAAGGGATTAAATATGACTTTTACAACTGATTTTCTCCATTTCTGAT
CTTTGCTAAAAGTAGATGATTATGAATTTCTTTAAGAAGTACTCGTACGATACTAGATAAGAATTT
CTATTTGCATGCTATAGCATATAAATTAGCATTATTGTTATTATCATACAAAGTATATTTTAAAACTA
GCTAGCAAGTAACAAAGATTGTTATTCCGTACTTTTAACGTCCCTTAATAATGTATCAAAACTATT
ATTTTCGAGAAGTTTCAAAATAGAAATCACACTTCTTTTTCCTGTTTTTACTATCTATATTATCCACT
GTTTATATATACTCTGTACTAAGTACTAATTCAAAGTGTGACGATTACTACGGGATGGATCGGAGG
GTGAAGGAACGAGAAAGGTTTATAAGTTTATAGAGTTTATTTACTTGAGACGGTCAAATTATTCA
GGGAAGAAACGTTTATTTTGTTGTTTCTTGAACGTTAACATGATAAAGAAAAATTGTACAATAATT
ATAAATTCTTCGTATGTGATAATGTTCAGTGGGCAAGGTTTCTTCCGCACACCCCAGCTGGGTCA
CTTTCGGAGAGCAGCAACACCAATTAA

>*CqYAB10*

ATGGCAACACTTAACCGTTTATTTGATACTCAAGAACAAATATGTTACGTTCAATGCAGTTTTTGT
ACCACCATCTTACTGGTAATCTTATGGCTCCAATGTTTTTACGTACCAATATTTTTGTATACATACAT
TCGTAGTTAATTAAGTACTCCTCCATATATACATCCTGAACAAATGTATGGTGACGACGTTTGTTG
ATAGTATAATTTGAAGTATTAATGTAACAACGTACATACTCATCCTGATTAATTAGGTCCGGATTAG
TCTATATATCTGTGCTTTAATTATTATTTCCTTTTTGTCGTTTTGTACTGGGAAGGTGAGTGTGCCAT
ATAGTAGCATGACAATGGTGGTGACAGTGAGGTGTGGTCATTGCACTGGTCTTCTCTCAGTCAAC
ATGTTGAAAGCTTCCTTTGTTCCCCTCCATCTTTTTTCTGCCCTTAACCAAGATCAGGTAATACTAA
TTAAGTTTACGAAATTAATTCTTCCCATATAGTTATCTAAATAGCCACATTTTTAATGTTTTTCCCTA
TTATTTATCATTCTTATAATATAATCTACTTTGGTTTCCTTTTATCACAAATAATAATGGAAAATTGT
CCTCACTTAAAGAAATCATAAAATGTTCATCTTTTAATTATTAGCAATATAATGAAATACAATACTA
AGTAAACTCGATATTCTTGTATATATTTGTTATTAAGAGACCTCAAGTACTTTTGTACACCACAAA
ATTATGAATAATTGTAATCTAAAGTTTTTTTCGGACAATATAACCTAGCTTATTGAACTTAATTATTG
ATTTAGAAATACATGTGTTTTTTATAAAGAAAAAGTTTACCCGTATCATGATTTAATTAATTTTGCA
TAGGTAAATTTTAAGCACCAAGAGGAAGTAGATACATCAAAGGCTATGGACAGGCACAGTACTA

CTTTGTCCATGTTACCCTCTTCGGAAGAAGATAATGACGATGAGGAAGAGGATGATGAAGATAA
CATTGCAATTGAGCAAATTGTTAATAAACGTATACACTTCAAATTTGATGTTCATGTATATATTATT
GAATTTTTTTCGCTGTAGTATCATGACCTAACGTGATTACAAATATTGTTGTTGTTTTAATTAATTGG
CAGCTCCAGAAAAGAAAAGAAGGGCACCATCAGCTTACAACAAATTCATCAAGTATGTAAATTC
GACGTTATTATTTATTCTTAAATTTTCATTCAAGCATCTACTTATACTATATGATATATAGAAATAAA
TTAATTCATTAGTATATATAAATAAATAATAAACCCTTAATAATTAACATGTGTGTTTTGAATAATAG
AGAAGAAATCAGGAGGTTGAAGGCTAGGAATCCTAATATGACTCATAAGGAAGCCTTTAGCACT
GCTGCTAAAAACGTAAGCTTAAATTTGTCACCTTTTTTATTTGATCTATAAATTGATGATAAAGAG
TAAAATTAAAATTTTAAATGTCTAGCTATAGTTTTATTTGATTTGTTTTTTTTTTTTTTTTTTTTTGTC
TAGCTATATTTTAAAGTGTAAAATTAAAATTTTAAATGTCTAGCTATATTAATTTTTTTTGCTTTATTT
GATTTGTTTTTTCTTTGTTAATGATTTTGCAGTGGGCTCATTTCCCGTCGGTCCAACATGAAGTTGA
TGAAGATAATGGTAGCCATGAGAACAGCATGACACAAAACTTAGACGACGAAGATTAA

>*CqYAB11*

ATGTCAAGCTCTAATATTGCCGCCTCTTCAACAACAACAACAACAACAACAACAACAACAAGC
TTGTCATTGGACCACTTCCATCCTTCTGAACAACTCTGTTATCTCCAATGCACTCGCTGTGAGACC
GTCCTTGCGGTATTCTTTCTCCTCGCTCTTTTCATCATTCATCATTCTCTTCTCACTACATACATACC
ACTCTCTTATTTATTCATTCAATTCGATCGATTTCTTCGTCATCTCAATATCTATACTACTTGATGTAT
ATGATTAATACCGGGCCGAGATACTCGGTTCATTACTTAATTAATTGGTTTTTATTGTTGTAACTAT
CCCTAATCTACTTCTATGTTTGTTATTGTTGTAGGTATATGATTTTATTTTAGGGTTTTAATTTATGAA
AACTAACTATTGGAAAAAAAAAATAAATAATGTAATAATTTGGACAGGTAAGTGTGCCATCAAGC
AGCTTGTACAAGACGGTGACGGTTCGATGTGGGCACTGCACGCATCTCCTGCCGGTGAACACAC
GATCTCTACTACTTCAGCCGCCGCCGGCTAGTCAGTATCACTTGCCTCATCATCATAACTACTACT
CTCCAAACTCCCATTATCGTCTGGTATTACTACTATCCAATTTATTAGCTAGGGTTTCTATTTTTAGC
AATTCAGAAAGCCCACAATTTGTTTATACTAGTATGTTAATTGAATATAACCTGATTTAATTGGTGT
AAGTTAAATGATCATATTCAAGCATAATAACAAGATTCTAATTCTTCATTGATTATTATTATTATAG
GGGGAGATGCCAAATCAAGCACCAAATTTCTCACTAACACAATCAAATGGTGCATCTAGCTACA
TGAATCCATCATCAAGCCGAGGCGGTTCAAACGAGCTTCCAAGGGCTCCTACCACTAACAGACG
TAATTCATCTATCTCAACTCTCAAGTAGTACATACTCCATGTTAGTTAATTAATTAATTAATTGAAA
TTTGTTTAAGGAGGAGCTGAGAAAAGGGGGGTGGGGATATGGAGGGATGTTCCTTTAATGCCTAT
GTCAACTTTTTGCAGCAACTTTGTCATTGATTCAGTGTTTGTTTCAGCACTGGATCAAGACTTTCT
TACTCTCTACTTTGGCCTAGCTATTGCTCCTTAATCAATTTTTTTTTTTTTTTTTTTTTTTTTTTTTTA
AATTTAAACCAACAACGAACTTGTTTTTGGTCAGTTTCTTTCTGTACATATAATTTTTTTGTACATA
TAAATGTCACAAACTACTATATACTAGCTAGTGGTCGCTGGTATGCAATATATATAAGTCACAATT
TTTTTTTTATTTTAAGGAGTATTGAATTTTTCTTATGTTAAATTGTACTCGGTAGTGAAATGTGTGAA
AAGTCTAGGCAATATACAAACGTTTAATCTATTTTTATTTACATAGATGAAGTCCTTTTTTTTTCAA
AATCGGAATTCTTAATTGATTGCTTCAATAAATGAATTGGTTAGGGCACATGTCAATTATTAATTTG
TTCATATTGATCGGATCGAATGAAGAAGACATATGAAAGAGCAAAGAAACATGGATGTTTCTTTT
AGAGATGATAGAGGGGTTTGTTTGAGGTTCATAACAAATGGGTTAACATGGTAGAAAAGGATAA
TACTGTAATTAATACCTTCTTTAACCAATAAACCAAGGACAGGTTATAAAGATGATATTGTTTTTGT
GTTTTTGTCTTTATATGTCGATCAGTGAGTGAATGAAAGAGATATTACTATCATTGTCTTCCGGAAG
TTTAGGGTAAAGTATTAATCATTTACTTGTACAGTTGTTTGTGTCTATACAATCATCATTGCATAAA
ATTAATATAAAAAAGTTAGTTTTGCAATATTCCTATAAGTTTAGGAAGTTTATACGAAAATTGAAA
AAAAAAAATATAGATTTCCTAAAGGTGTCAATATGGAGCATATTGAAAATTATGCATGATTTGTTA

TTAATAACACTTTTATACTTTAAACATGTTATGCACTTATGCATATGACATTAAGAAAATATCCATA
AGTTTGAACTCTTGAATTTAATTTTTTATTTTTATCTTTTGGTTAACTATTTTTTCATATTTAAAGTGT
TTCTAATTTTCTATTAGTATTCATATATAAACTTATTTAAATATTTTTTGCTAAATTTGCATTTGGTTTA
CAACAGCACCGGAAAAAAGACAAAGAGTGCCTTCAGCTTACAACCGATTCATCAAGTGAGCAA
TCGTTCTCTTTCAATTTAACTCTATAATACGAGTAATTTGTTTTTCGAATAAATATGTGTATGTCTGA
AACCTCAAAATGAATTGTACTTCATATGTGCAGAGAGGAAATCCAGCGTATTAAGGCTGAAAAT
CCTGATATTTCTCATAGAGAGGCTTTCAGTGCTGCTGCCAAGAATGTAAGTTTACTTATTGTACTC
TTCACTAATCATCTATGTTGCTCTTGGCTTAGTATAACATGTTTCAAGCATTGTAATTTGTGAAAAA
AAGTTCCCAAATTTCATAGATTTTTATGGTTATCGATTTTGCTAGCTAGTCTTATTGGATCCTAATTC
CTATTTTTTTATATATACAACTCAATTATGTGATGAAATTAATACAGTACAACGAACTCCTTTTTGG
CTTCATTTACATGCTAAATAAGTAAATAGTAATTGAAAATAAATGATGTAAATGAGATTTGACAAT
GAATTCTCGGAACCAACCCTGTGACTTTTACTGCCAAATCAAAAATGCTGGATTATTACTAGCAT
CTACTTAATCGTGTTAAGTTCCAAAGTCAAATGTTAGGGTTTCATGACAGTTTACTGTATGTATGA
GTAAAAAAATTTTGAAAAATGGCTGAAAAAAATATTCTAAATATGTTAAATCAAACAGTGGGCC
CACTTTCCCCACATCCATTTTGGGTTGATGCCGGAACGACGGTGA

>*CqYAB12*
ATGGAGTTCAGTACATCAGCAGAGCGAGTCTGCTATGTCCACTGCACCTTCTGCAACACCATTTT
AGCGGTACTCTTTTCTCTCTCTCCTCTTTTTTTTATTTATTTCTTTATTTAAGAAAGAAAAAATCTCC
CACATATTCTGTATCTTTGAGTTTATTAGATCAGATTTTATTAATTTTTTTTTTCTATTTCTGATCAAG
TTTTAGGTTTTGCTAAGCAAATTAAAAGTTTGTACTAATAAATATATTTTTTCCGTAGATAACAAAA
AGAAATGGAGGGTGTATAAGCTACCTTTAACTTTCTGAAAAAAAAATTATGAGTTGTTAATTAAG
CAGCCTATGTATGTATAAAAGTAGGGTTGCAAACCTTAATTAATAAAGTATATATAAATTAGGGTT
CATCATCAATGAAACAGGTAGCTAGTCTAGCTAATTATTAGTATAATTTAATTTGTTTAGTGTATGT
TTAATCCAATAAAATTTCATATGTACTTCAACATTATTATGATATTACCTTACAAACTTATACTTTTT
TTTTTTTTGAATGATGACCACATCTCACTGATCTCAGTCACTTATGATTCTTCATCTGTACCAAAGT
GTTGCATTGGTACAGTCTCTCTTTTTTCCTTGGCATGTAAAAAATTGAGTTTTTTTTTTTTATTTATTT
TTAGAAAATAAAAATAAAAATCCCTCCTTTGCTTAAGTTAATAACAAAAAACTTTATTTAATGATT
TATTACTTTGTACTAAGAGTATAATACAACATTAGTTTTTCTTTCTTGGTATACAAAAATTATTACTT
CGTAAATGAATGATACTCCCTATTTCATATGATCTGGATCGATTTATAACTTAAAATGAGTGAGATC
GTTTTTACTTTAACTTTTTTTTTTTTTTTTTTTTTCTCTCTTGACTTTTATGTTGGGGAGAAAAAGGG
GATGTCAGGGTTTGTGAACCCAAGCCAGAAACTGCAGCAAAAATGGAAAGAGATTTCTCATTTA
GGGTTGCCTTTTTCATGTTGTGTACCACACTTCATGTAACTTTATACTCTACCAAGTATATTCTTTTG
ATCGCGTTGTTATTCGTTAACTACTCGTAAATCTATTATTCTTAGTACAAACATAGATAACAATTAC
GGTGACAATTCAATACGAAAAAAATCTGTTATCCACTCTGTTCACACTATTAATTTTATGAGAGTA
TCATCAATCGAATTATTTTTCGAAACCTAATCTAAGTTATGAATTTAATTCATTGGTAAAGTGTATT
TTAGCCTATTGATGTGGTGTGTGGGATTAAATCGACTCGATCCTCATTTAAGATCGATATGTAGGA
CTTGTATACTACCTCCGGATTATATTATATGCAACTTTGGAATTTTTTATATCTCACAAATAATACCC
CAAAGTTGCATATAATACAATCCGGAGGTTGTATATGAAATGTCAAATGTTCAAGATCTTCTCCCA
CATGGGATTAGGATTGGTTTTGGTGGTACTATTTATTTTCTAGTCCTTAAAATAATACTTTTAAATCA
ATAAAGTATTTAGTTGAAGTGTAAATATAAATCAATTCTCCTTATTGCTTTTTTTTTTTTTCCATTTG
GTCCAATCTTAGTAGCTAGGTTTTACTTGATGATATGTTTCTCAACAAATCTAGATCATCATGAGAT
TGACGGTAGGTCATGTTGATAATGTAATTTGAACTAATAATATGATAGCTAAATCTACGGGACTAT
AAATTAGTATTTACGACATATTTCATTAAATCTAATTGATTAGCTATTAAAACGCGGCAGGTAAGC

GTACCATGCTGCAGCATGTATAATATGGTAACAGTCAGATGTGGGCATTGTGCCAATCTTCTCTCT
GTTAACATTGGACTTTCACCTCCATCTATGCCTTATCAAGATAATTTCCAGGTATAATTTACTTACA
ATTGATCTCATTTTATTATTTTATTTTTTCTTTCTTTGCAATTATTTGAACAAAGTGTATGTTAAGAA
ATTAAAATCTATATATAGTTGCTGAGGCAGCACTATAATTATCAAGATGTGAGCAGAGATAGTAGT
AGTACTATAACAGGCGGCTCATCGTCTTCAACTATTACTACAATCGCTTCTGATGATCATGATGTT
CAACAGACTCGTCCCCCACCCATTCGTCGTAAGTACTTAATTTTTTCAGCTAATCTTAATTCAATT
GATATATTTATACTACCTCCGTTAAATGGTATGTGTAACTTTGGGGAATTATTTGTGAGATATAAAA
TATTCCAAAGTTGCACATAACATTTAACGGAAGTAGTATATGAAATGTTTAGATATTATGGTTTGTT
AGTTGTTACTCTTCATAGTTGATCATAATTAGGTCCCTTTTTGTTTTTGAGGATTTAAAGTCGTATA
ATGAACTTAAAATTATAGATACATTTATATATGACTATATGTATGTTACGGAGTAATATAATAAACC
TGAAAACCGAACTTCAATAGATCTTTAACAGCAACAAACAAGCCCTAATTCTTAGGATTTTTGTT
CAACAAACAAGCTAGCCCTATAAAAGAAAGTGTTTTGATTTCATGAAATTAACCTAATGAAGATT
TGAACATCCAACTTAATTACATATTTGTTCAAATTAAACATAAAAGCATTACTTCGACTTGATCAA
AGGGTAACGCGAAGCATTAAAACACACACAAACACGCAAACACACAAGAGCTTTAACCATGTT
ATTTGTTGGCTTGTTGCTTTCTAAGGCATTTAGGTAAACTCTAATTGTCTTATATGTTTATTGTTGAG
CAGCCCCGGAAAAGAGGCAACGCGTGCCTTCGGCTTATAATAAATTTATCAAGTAAGGATTACTA
AATCCCCATTGCAAGTTCTAGTTAATCTCTCTATTTGTCTACTTATAACATACTTATTCATCTGTTAG
TATGAACTTATAATTGTTTGGTTTTCTCATGCTCACTCTTAAATTTTTAATTAAATATGTTGTTAAGC
ATGAAGCATTTGCTTTTGAAAACATAAAAAATAAACTTTTAATACTAATTAATTTGTTACATGTTG
AATAATACTGTAAAAATACTAATATACTAATTTGTTACCCCCAATTTTTTATTTTTTTGTTTCCCCTTT
CTTAATAATTTGGTTCTCTATTGAAGGGAGGAAATCCAAAGGATAAAAGCCAGCAATCCTGAAAT
TAGCCATAGAGAGGCCTTCAGTGCAGCAGCTAAGAATGTAAGTCTTTTTATGTTCTAAAAGTAAC
TTTAATTTTATGTTATTTCACCTAATCTGCTATGATCCCATCTAATCCGACTTGATTGTGTCTATCAT
ACATACATTAAATGTATACATGATTATTACAGAGTACTTGATAAATTCGTCGATTTTACTGCATGTC
TATTACACAATTATTATACACTCGTATGTACGTAAAATAATAATTAGTTACACCGATGCTGACGTTA
GTAAACTGTAGAAGCTAATAGTAAACATAATGTTGGCTTGGCAGTGGGCACATTTTCCTCATATTC
ACTTTGGCCTAAATCTGGACGGGCAAAGCCAAGCAAGGTTGGACCAACCAGTTTCTGGAGAAG
GGACAAACAAGTCTCATGGATTTTACTGA

Supplementary File S4

YABBY protein sequences in *C. pallidicaule* (A-genome)

>AAA12895-RA
MEFSTSAERVCYVHCTFCNTILAVSVPCCSMYNMVTVRCGHCANLLSVNIGLSPPSMPHQDNFQLLR
QHCNYQDVSKDSSSTITGGSSSSTITTMASDDHDVQQTRPPPIRPPEKRQRVPSAYNKFIKEEIQRIKASN
PEISHREAFSAAAKNWAHFPHIHFGLNLDGQRQARLDQPVSGEETNKSHGFY

>AAA11906-RA
MSTLNRLFDTQEQICYVQCSFCTTILLVSVPYSSMTMVVTVRCGHCTGLLSVNMLKASFVPLHLFSALN
QDQVNFKHLEEVDTSKAMDRHTPEKKRRAPSAYNKFIKEEIRRLKARNPNMTHKEAFSTAAKNWAH
FPSVQHEVDEDNGSHENSMTQNLDDED

>AAA01885-RA
MMNTNMMEDKVGSELAPPPPEHLCYVRCNFCNTVLAVVIPCKRLLDTITVKCGHCSNVSFLSTRPPL
QGQCLDHQITLQGFNFFEKPGGFCSTIDHHSNKKIEPSPSMSSTLTEPVSPRPFVCKPPEKKHRLPSAYN
RFMKEEIQRIKAANPEIPHREAFSTAAKNWARFLPHTPAGSLAESSNIN

YABBY proteins sequences in *C. suecicum* (B-genome)

>BBB02023-RA
MSSTSCVDQAAVAPPSSEQLCYIPCNYCNIVLAVSVPCNNLFDIVTVRCGHCTNLWSVNMAAAFHSLS
ASWQQHQQQNFHQAPNNGNMGEYRIDNLGSSSKCNYTSKAATTMRISPPINNNSAEERIINRPPEKR
QRVPSAYNQFIKEEIQRIKANNPDISHREAFSTAAKNWAHFPHIHFGLMLETNNQPKLDE

>BBB05628-RA
MRLTVGHANNVSVPCCSMYNMVTVRCGHCANLLSVNIGLSPPSMPYQDNFQLLRQHYNYQDVSRD
SSSTITGGSSSSTITTIASDDHDVQQTRPPPIRPPEKRQRVPSAYNKFIKEEIQRIKASNPEISHREAFSAAA
KNWAHFPHIHFGLNLDGQSQARLDQPVSGEGTNKSHGFY

>BBB04920-RA
MNTNTMEDKVGSELAPPPPEHLCYVRCNFCNTVVIPCKRLLDTITVKCGHCSNVSFLSTRPPLQGQCL
DHQITLQGFNFLEKPGGFCSTIDHHSNKKSEPSPSSSSTLTEPVSPRPFVCKPPEKKHRLPSAYNRFMKEE
IQRIKAANPEIPHREAFSTAAKNWARFLPHTPVAESSNTN

Supplementary File S5

YABBY-related sequences from clubmosses and Micromonas species

>ATG86193.1 transcription factor YABBY [*Huperzia selago*]
MSSCSGMMNSPPADEQSSCCDESLVDDRCTKCHVHCGHCHVILTVDVPHNLLDKNSVLVRCGDCRS
LISVNIQSLAENQSGLVSRVRKNHEEGGERTNDESSIISSSEHFASSGKSLQSILSSPPAEAVKPPKPKRRN
QSSRGDDSSILASRGKKPRTPSAYNMFVRDEILRIKAKDPTISHKEAFIAAAKNWATQPHINLGTRSEHR
DKKIEEKCLNRTV

>KAJ7562872.1 hypothetical protein O6H91_03G087300 [*Diphasiastrum complanatum*]
MSSYHGMMHSLPADEQSSCNDESSVDDRYIKCHVHCGHCHVILTVDVPHSLMNRSSVLVRCGDCRSL
ISVNIQSLAEHHSSLENDGEGGQRTNEESSMVSSSDRVTSSGKNLRSMPSAPVVAVKPPKPKRRNHSSSS
DDVSIAASRGKKPRTPSAYNIFVREEILRIKAKDPTISHKEAFIAAAKNWATHPHFHLGTRSEYRDKKSD
EKDTEEVRYAESVIYKGTTKGCRTRVVGKSAW

>tr|C1DY70|C1DY70_MICCC Yabby-like protein OS=*Micromonas commoda* (strain RCC299 / NOUM17 / CCMP2709) OX=296587 GN=MICPUN_112717 PE=3 SV=1
MGPNLQAASGSNMSDAAAAEARWARCDAPTDVDAVARGDVDRARDDGSKTVHVDCQRCRSRLE
VRVPAALLAEGSATVRCGACGVHLKIAVPPALAPVHPPRPAFSAMTKPAERLPAASAPRPTQQRPAT
GASLQLSAGALASFLDPAVCVAMGANPTDPQLRKAAEEFWRSCDGDANAVDPNATYDTDLAPARP
AKRAKKTRKPRDPSPYNVFIREEIPRLKAENPAMTHKDAFKAAARNWAGSSLNMRSAAYVPDPVLA
AAAAAAHANVLDAHANVTDAATRAAILQKLKPHLLRGRVEDARAEAAAARRWEMDLTTTNGVD
GPTGAKGVVDRYAGDAFASVHEGRDEPTHRGPVRPKTSWSSYRSTHEAFEPID

>tr|C1MQU4|C1MQU4_MICPC Yabby-like transcription factor OS=*Micromonas pusilla* (strain CCMP1545) OX=564608 GN=MICPUCDRAFT_57415 PE=3 SV=1
MTADASADVAAAVAGLVDDDRGTLVHVDCDRCRSRLEVRVPSSLRVDARGVAVRCGACETLLQVA
VPPLLSPSTPGLFARGEPGDGIGLAPPGSLLSGDRVSGGGDDDDGGAGAGVAGCGPGRSRASNPEH
ERHLRLARYHMDMAAQHSNPSAGHVSFPAMPATSPLAQLSLPPCEHESDERVDRVLRAAAHEFWW
NPSEIPHRGRRDDDDDYYDANPRHQKILKRERKPRDPSPYNVFIREEIPRLKEKDPGLNHRDAFKAAA

KNWAHSPLNMRSPAFVPGPTDRPAERDDDDDERRAGEVDEDDSAAARAEIMRKLQPILQSTPTREE
GTDTAAAAAGRGDGDRANEAHESTPRRDSDRDEDADKTSAGERRGTSSHRTKSSEHSTECKEYTSC

>XP_002499636.1 yabby-like protein [*Micromonas commoda*]
MGPNLQAASGSNMSDAAAAEARWARCDAPTDVDAVARGDVDRARDDGSKTVHVDCQRCRSRLEV
RVPAALLAEGSATVRCGACGVHLKIAVPPALAPVHPPRPAFSAMTKPAERLPAASAPRPTQQRPATGA
SLQLSAGALASFLDPAVCVAMGANPTDPQLRKAAEEFWRSCDGDANAVDPNATYDTDLAPARPAKR
AKKTRKPRDPSPYNVFIREEIPRLKAENPAMTHKDAFKAAARNWAGSSLNMRSAAYVPDPVLAAAA
AAAHANVLDAHANVTDAATRAAILQKLKPHLLRGRVEDARAEAAAARRWEMDLTTTNGVDGPTG
AKGVVDRYAGDAFASVHEGRDEPTHRGPVRPKTSWSSYRSTHEAFEPID

Table S1. List of *YABBY* genes in *Spinacia oleracea, Beta vulgaris,Arabidopsis thaliana* and *Oryza sativa.*

Gene name	Gene ID	Chromosome location	Species
SoYAB1	Spov3_chr5.01174	chr5:13785462-13789119	*Spinacia oleracea*
SoYAB2	Spov3_chr6.01719	chr6:25016034-25019047	*Spinacia oleracea*
SoYAB3	Spov3_chr2.00033	chr2:438633-442032	*Spinacia oleracea*
SoYAB4	Spov3_chr4.04704	chr4:118332792-118334653	*Spinacia oleracea*
SoYAB5	Spov3_chr5.02270	chr5:36618488-36621731	*Spinacia oleracea*
SoYAB6	Spov3_chr5.03629	chr5:81326671-81327963	*Spinacia oleracea*
BvYAB1	EL10Ac1g00847.1	Chr1_EL10_PGA_scaffold3: 10903605-10908609	*Beta vulgaris*
BvYAB2	EL10Ac7g16274.1	Chr7_EL10_PGA_scaffold5: 5624126-5632125	*Beta vulgaris*
BvYAB3	EL10Ac5g13007.1	Chr5_EL10_PGA_scaffold2: 58218134..58224183	*Beta vulgaris*
BvYAB4	EL10Ac6g13704.1	Chr6_EL10_PGA_scaffold0: 9155588-9157688	*Beta vulgaris*
BvYAB5	EL10Ac8g18683.1	Chr8_EL10_PGA_scaffold4: 5786571-5791258	*Beta vulgaris*
BvYAB6	EL10Ac6g15025.1	Chr6_EL10_PGA_scaffold0: 54569157-54572302	*Beta vulgaris*
AtFIL	At2g45190	Chr2:18628251-18630779	*Arabidopsis thaliana*
AtYAB2	At1g08465	Chr1:2675812-2679824	*Arabidopsis thaliana*
AtYAB3	At4g00180	Chr4:72544-75576	*Arabidopsis thaliana*
AtINO	At1g23420	Chr1:8317296-8319491	*Arabidopsis thaliana*
AtYAB5	At2g26580	Chr2:11303454-11307010	*Arabidopsis thaliana*
AtCRC	At1g69180	Chr1:26007349-26009141	*Arabidopsis thaliana*
OsYABBY1	LOC_Os07g06620.1	Chr7:3221591-3229297	*Oryza sativa*
OsYABBY2	LOC_Os03g44710.1	Chr3:25197056-25206961	*Oryza sativa*
OsYABBY3	LOC_Os10g36420.1	Chr10:19471067-19475406	*Oryza sativa*
OsYABBY4	LOC_Os02g42950.1	Chr2:25839310-25842729	*Oryza sativa*
OsYABBY5	LOC_Os04g45330.1	Chr4:26797649-26800253	*Oryza sativa*
OsYABBY6	LOC_Os12g42610.1	Chr12:26477632-26487388	*Oryza sativa*

OsYABBY7	LOC_Os07g38410.1	Chr7:23086433-23088524	*Oryza sativa*
DL	LOC_Os03g11600.2	Chr3:6041244-6048687	*Oryza sativa*

Table S2. List of primers used in this study.

Primer name	Gene	Sequence（5'→3'）	Purpose
SX-S-01	*CqYAB1*	ATTAAGAAGTGTAAACTGTTTT	qRT-PCR
SX-A-02		AATAATATTACGTTCTTAACG	
SX-S-03	*CqYAB2*	AAACACCTAATAACAACAATA	
SX-A-04		TGAAGGCGAAGGCAAAAGATG	
SX-S-05	*CqYAB3*	ATTAAGAAGATGAGTAAACTG	
SX-A-06		CAATAATATTACGTTCTTAGCA	
SX-S-07	*CqYAB4*	ATGAGGAGGAGGATGAAGAAG	
SX-A-08		AAGGCTTCCTTATGAGTCATG	
SX-S-09	*CqYAB5*	AAGTGAACCATCTCCTTCGTC	
SX-A-10		TCCGCAAGTGACCCAGCTGGAG	
SX-S-11	*CqYAB6*	TGTAATTATCAAGATGTGAGCAA	
SX-A-12		AGGAACGCGTTGCCTCTTTTCT	
SX-S-13	*CqYAB7*	CCGCCTCTTTCGCCGAATCACCA	
SX-A-14		ACTCCTCGAACGGACATCATT	
SX-S-15	*CqYAB8*	TGGACCACTTCTCACCTTCTGAG	
SX-A-16		AGTAGTAAAGATCGTGTGTTCG	
SX-S-17	*CqYAB9*	TAATGTATCTTTTCTGAGCACC	
SX-A-18		TGACATCGAAGGAGATGGTTCG	
SX-S-19	*CqYAB10*	AGAAGGGCACCATCAGCTTAC	
SX-A-20		TCATGCTGTTCTCATGGCTAC	
SX-S-21	*CqYAB11*	TGGACCACTTCCATCCTTCTGAA	
SX-A-22		AGTAGTAGAGATCGTGTGTTCA	
SX-S-23	*CqYAB12*	TATAATTATCAAGATGTGAGCAG	
SX-A-24		AGGCACGCGTTGCCTCTTTTCC	
LT-S-01	*CqACT2*	CCCCTGCTATGTATGTTGCAATTC	
LT-A-02		AGTGGTCTGTTAGGTCACGACCAG	
LT-S-03	*CqRAN3*	TTGGTGTCGAAGTTCATCCATTGG	
LT-A-04		GTAAGTCAATCGAGCAGTCACATC	
SX-S-25	*INO*	ACTGACGCGTTGAAGAGTAGGTTATGTCTTTTTG	promoter
SX-A-26		TGCACCCGGGAGAGAGTGTGTGTGTACGATG	
SX-S-27	*INO*	TGCACCCGGGATGAATATTAACAAAACCTATC	CDS
SX-A-28		AGTCTAGATTACTCAAATGGAGATTTTCCC	
SX-S-29	*CqYAB4*	TGCACCCGGGATGGCAACACTTAATCGTTTATTTG	
SX-A-30		AGTCTAGATTTCAATTTGCTTCATCTTCGTCG	
SX-S-31	*CqYAB10*	TGCACCCGGGATGGCAACACTTAACCGTTTATTTG	
SX-A-32		AGTCTAGATTAATCTTCGTCGTCTAAGTTTTG	
